# Reprogramming *Yarrowia lipolytica* metabolism for efficient synthesis of itaconic acid from flask to semipilot scale

**DOI:** 10.1126/sciadv.adn0414

**Published:** 2024-08-09

**Authors:** Jing Fu, Simone Zaghen, Hongzhong Lu, Oliver Konzock, Naghmeh Poorinmohammad, Alexander Kornberg, Rodrigo Ledesma-Amaro, Deni Koseto, Alexander Wentzel, Francesca Di Bartolomeo, Eduard J. Kerkhoven

**Affiliations:** ^1^ Division of Systems and Synthetic Biology, Department of Life Sciences, Chalmers University of Technology, Göteborg 412 96, Sweden.; ^2^ Department of Bioengineering and Centre for Synthetic Biology, Imperial College London, London SW7 2AZ, UK.; ^3^ State Key Laboratory of Microbial Metabolism, School of Life Sciences and Biotechnology, Shanghai Jiao Tong University, Shanghai 200240, China.; ^4^ Department of Biotechnology and Nanomedicine, SINTEF Industry, Trondheim N-7465, Norway.; ^5^ SciLifeLab, Chalmers University of Technology, Göteborg 412 96, Sweden.; ^6^ Novo Nordisk Foundation Center for Biosustainability, Technical University of Denmark, DK-2800 Kgs. Lyngby, Denmark.

## Abstract

Itaconic acid is an emerging platform chemical with extensive applications. Itaconic acid is currently produced by *Aspergillus terreus* through biological fermentation. However, *A. terreus* is a fungal pathogen that needs additional morphology controls, making itaconic acid production on industrial scale problematic. Here, we reprogrammed the Generally Recognized As Safe (GRAS) yeast *Yarrowia lipolytica* for competitive itaconic acid production. After preventing carbon sink into lipid accumulation, we evaluated itaconic acid production both inside and outside the mitochondria while fine-tuning its biosynthetic pathway. We then mimicked the regulation of nitrogen limitation in nitrogen-replete conditions by down-regulating NAD^+^–dependent isocitrate dehydrogenase through weak promoters, RNA interference, or CRISPR interference. Ultimately, we optimized fermentation parameters for fed-batch cultivations and produced itaconic acid titers of 130.1 grams per liter in 1-liter bioreactors and 94.8 grams per liter in a 50-liter bioreactor on semipilot scale. Our findings provide effective approaches to harness the GRAS microorganism *Y. lipolytica* for competitive industrial-scale production of itaconic acid.

## Teaser

Partial mimicking of the regulatory response to nitrogen limitation enables high itaconic acid production in *Y. lipolytica*.

## INTRODUCTION

Itaconic acid (IA) is a promising platform chemical with extensive applications in food, textile, and pharmaceutical industries (*1*). IA ranks among the top 12 building block chemicals (*2*) and can be transformed into various valuable bio-based products. Through cross-linking, IA enables the synthesis of many innovative polymers with remarkable properties (*3*, *4*), e.g., shape memory polymers (*5*), polymeric hydrogels used for targeted drug delivery (*6*, *7*), and material against bacterial infections (*8*, *9*). In addition, IA is secreted by mammalian immune cells as part of the immune response (*10*, *11*). Recently, IA was shown to act as a signaling molecule in immunomodulation (*12*), and it is a crucial anti-inflammatory metabolite that limits inflammation (*13*), exhibiting potential applications in the biomedical field (*14*).

Currently, commercial IA production relies on biological fermentation rather than chemical synthesis (*15*), but the microorganisms used are not ideal for cost-competitive economical IA production at the industrial scale. IA is primarily produced by the filamentous fungus *Aspergillus terreus*, which can produce 160 g/liter IA (*16*). However, IA production by *A. terreus* remains challenging. First, *A. terreus* is a fungal pathogen that can cause lethal infections (*17*). In addition, morphological control has a substantial effect on the IA production (*18*), and maintaining the required growth-form pellet needs careful monitoring of fermentation parameters (*19*). Furthermore, IA production by *A. terreus* is severely inhibited when Mn^2+^ exceeds the extremely low concentration of 3 μg/liter (*16*, *20*). To prevent this and to produce high IA yields, a cation exchange treatment of analytical-grade glucose (*20*) or a careful addition of calcium (*21*, *22*) or copper ions (*23*) is necessary. These factors increase operational costs and increase risks of failed batches (*24*). Another promising microorganism, *Ustilago maydis*, a ubiquitous pathogen of corn (*25*), can produce itaconate (yield of more than 220 g/liter) as solid calcium salt form (*24*). However, that fermentation requires manual addition of solid calcium carbonate as liquid suspension or powder whenever the pH drops below 6.2. Furthermore, the final product calcium itaconate requires in situ precipitation to alleviate product inhibition, posing a notable challenge to scale up. Besides these two native IA producers, numerous heterologous IA producers were engineered, such as *Escherichia coli* (*26*–*28*), *Saccharomyces cerevisiae* (*29*), *Corynebacterium glutamicum* (*30*), *Aspergillus niger* (*31*, *32*), and *Yarrowia lipolytica* (*33*–*35*). However, their IA production was not satisfactory, and production parameters were far from commercialization (*36*).

*Y. lipolytica* was recognized as “recommended biological agent for production purposes” by the European Food Safety Authority (*37*) and has been granted the Generally Recognized As Safe (GRAS) (*36*) status for various commercial scale processes (*38*). *Y. lipolytica* is gaining traction as a biotechnologically relevant cell factory and is arguably regarded as the most promising nonconventional yeast biocatalyst (*38*). The main advantage of *Y. lipolytica* as an IA producer, other than the availability of genetic manipulation tools, is due to its considerable metabolic flux toward citric acid (CA) and isocitric acid (ICA), which can be redirected toward IA via the intermediate *cis*-aconitate. Moreover, pseudo-hyphae formation in *Y. lipolytica* can be completely abolished by deleting *mhy1* (*39*), providing an easier morphological control compared to *A. terreus* (*18*). *Y. lipolytica* can synthesize and accumulate large quantities of CA (>100 g/liter) and lipids (*40*) under nutrient restriction (*41*, *42*), such as nitrogen limitation (NL), phosphate limitation (PL), or sulfur limitation (SL). However, *Y. lipolytica* ordinarily stores low amounts of lipids at nitrogen-replete (NR) conditions (*43*), which lowers the IA yield. IA is mainly synthesized during the nongrowth (*14*, *19*) phase in *A. terreus* and *U. maydis* when carbon source is abundant, but phosphate or nitrogen are limiting (*14*). However, in *Y. lipolytica*, there have been conflicting reports about whether IA production is growth associated or not (*33*–*35*).

**Fig. 1.**
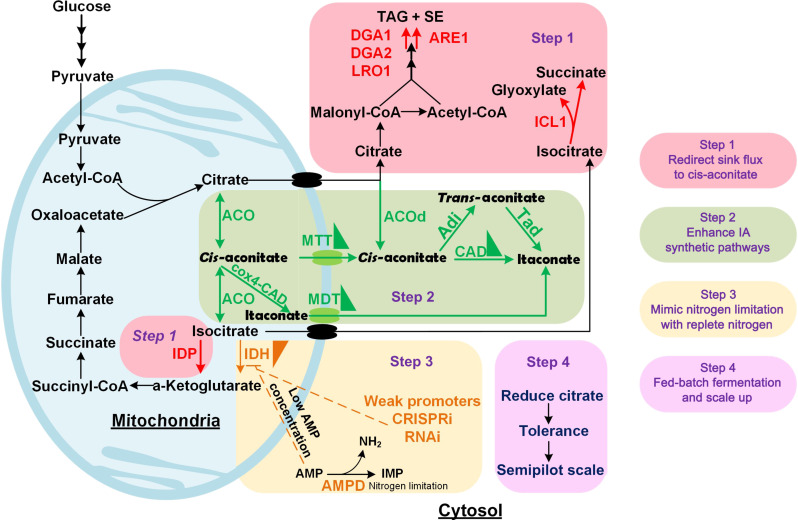
Reprogramming *Y. lipolytica* metabolism for efficient synthesis of IA from flask to semipilot scale. The whole work could be divided into four steps. Step 1: To redirect sink flux, there were mainly two nodes, citrate and isocitrate. *DGA1*, *DGA2*, *LRO1*, and *ARE1* were deleted to block the TAG and SE accumulation. *ICL1* was deleted to block the glyoxylate cycle, and IDP was deleted to reduce the utilization of isocitrate. Step 2: Enhance IA synthetic pathways. Step 3: The mechanism in NL was mimicked by down-regulation of IDH by weak promoter exchange, CRISPRi, and RNAi. Step 4: The scale-up from deep-well plates to benchtop bioreactors and semipilot-scale fermentation. ACO, aconitase; ACOd, aconitase without mitochondrial leading sequence; ADI, aconitate isomerase; AMPD, adenosine monophosphate deaminase; ARE1, Acyl–coenzyme A (CoA):sterol *O*-acyltransferase; CAD, *cis*-aconitate decarboxylase; DGA1, Acyl-CoA diacylglycerol *O*-acyltransferase 1; DGA2, Acyl-CoA diacylglycerol *O*-acyltransferase 2; ICL1, isocitate lyase; LRO1, phospholipid:diacylglycerol acyltransferase; MDT, mitochondrial decarboxlic transporters; MTT, mitochondrial tricarboxlic transporter; TAD, *trans*-aconitate decarboxylase.

In this study, we engineered *Y. lipolytica* for efficient IA production in four steps (Fig. 1). We reprogrammed its metabolism and established a competitive IA producer, testing it from a shake flask to a semipilot-scale bioreactor and addressing the following challenges within 4 steps. In step 1, we enhanced *cis*-aconitate supply by removing carbon flux from the competing lipid storage and by interrupting the glyoxylate cycle and nicotinamide adenine dinucleotide phosphate (NADP^+^)–dependent isocitrate dehydrogenase (IDP). In step 2, we introduced the IA biosynthetic pathway and enhanced IA titer/production by compartmentalizing its production either in the mitochondria or cytosol and by fine-tuning the expression levels of key enzymes. In step 3, we successfully decoupled high flux of *cis*-aconitate from NL condition by mimicking NL regulations without NL. We optimized carbon distribution between cell growth and IA production in NR conditions by down-regulating the expression of nicotinamide adenine dinucleotide (NAD^+^)–dependent isocitrate dehydrogenase (IDH). Last, in step 4, we optimized fermentation parameters and produced 130.1 g/liter IA in 1-liter bioreactors and 94.8 g/liter IA in 50-liter bioreactors. Overall, our work presents a notable leap and offers effective approaches toward harnessing *Y. lipolytica* for competitive biotechnological production of IA.

## RESULTS

### Enhancing *cis*-aconitate supply by reducing flux in sink pathways

Before establishing heterologous IA biosynthesis, it is desirable to enhance accumulation of the IA precursor *cis*-aconitate, by reducing the fluxes of alternative sink pathways through CA and isocitric acid (ICA).

**Fig. 2.**
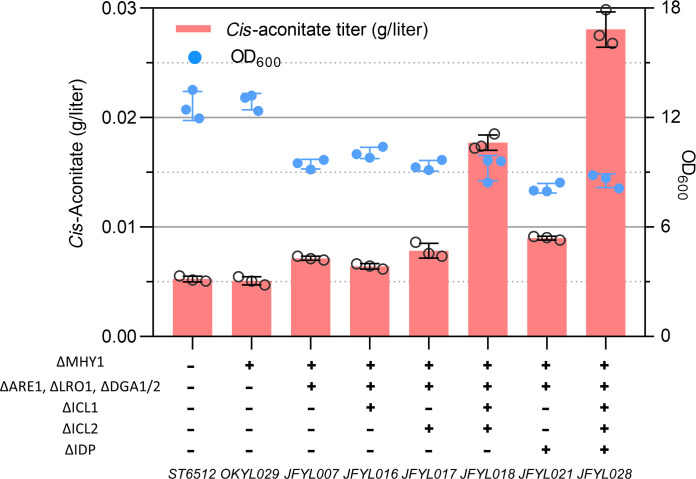
Redirect sink pathways to increase *cis*-aconitate availability. Strains were cultivated in 24–deep well plates after 4 days. Data represent the mean of *n* = 3 biologically independent samples, and error bars show SD.

To decrease CA-derived lipid accumulation, we deleted three acyltransferase coding genes (*DGA1*, *DGA2*, and *LRO1*) involved in triacylglycerol (TAG) accumulation and the *ARE1* gene involved in sterol esterification (SE) in the starting strain OKYL029 (fig. S1). The resulting strain, JFYL007 (also known as Q4 strain (*44*, *45*)), did not form notable lipid droplets (fig. S2), and its lipid content was strongly decreased compared to the wild-type/parental strain OKYL029 under NL or NR conditions, which triggers lipid accumulation (figs. S3 and S4). Because we later found that NR conditions increase IA productivity (step 3), JFYL007 was cultivated in NR medium, and *cis*-aconitate accumulation increased (Fig. 2), indicating that the lipid accumulation flux was redirected to *cis*-aconitate. To reduce flux through the ICA sink pathways, we disrupted ICA utilization by the glyoxylate cycle and isocitrate dehydrogenation. Codeletion of isocitrate lyase coding genes *ICL1* and *ICL2* (JFYL018) resulted in a 1.48-fold increase (Fig. 2). Disruption of isocitrate dehydrogenation involved two isoenzymes, IDP and IDH. The deletion of *IDP* (JFYL021) only increased *cis*-aconitate accumulation by 25.8%, but the final biomass decreased. Codeletion of *ICL1*, *ICL2*, and *IDP* (JFYL028) increased *cis*-aconitate accumulation by 2.93-fold. Contrastingly, five attempts to delete the open reading frames of IDH subunits coding genes *IDH1* or *IDH2* failed, suggesting that the NAD^+^-dependent IDH is essential for *Y. lipolytica* survival on YPD medium plates. In NR conditions, no CA or ICA was detected in any of the above strains.

### Enhancing IA production by optimizing its synthetic pathways

In *A. terreus*, mitochondrial aconitase (ACO) converts CA to ICA in a two-step reaction. *Cis*-aconitate, the intermediate of this reaction, is transported to the cytosol and converted to IA by *cis*-aconitate decarboxylase (CAD) (*46*). Contrastingly in *U. maydis*, aconitate isomerase (ADI) converts cytosolic *cis*-aconitate to *trans*-aconitate, which is then converted to IA by *trans*-aconitate decarboxylase (TAD) (*36*). These two IA synthetic routes, both outside and inside mitochondria, were constructed and evaluated in *Y. lipolytica* (Fig. 1, step 2).

**Fig. 3.**
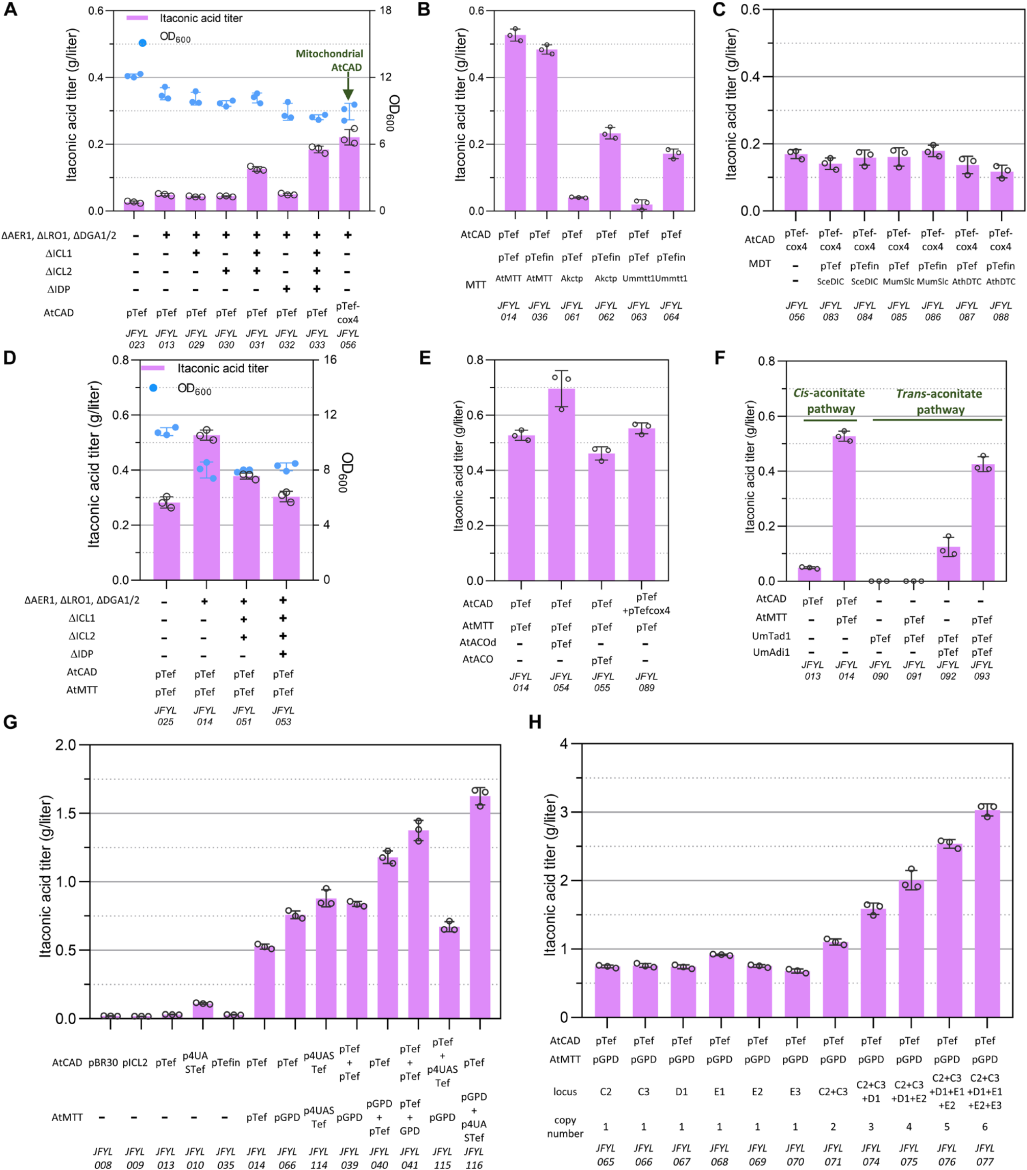
Optimize IA synthetic pathways. Strains were cultivated in 24–deep well plates after 4 days. (**A**) IA production with the introduction of AtCAD in IA sink pathways blocked strains. (**B**) IA production in cytosol with three different MTT transporters under pTef and pTefin. (**C**) IA production in the mitochondria with three different MDT transporters under pTef and pTefin. (**D**) The combination of promising targets from step 1 and the selected AtMTT. (**E**) Introducing the ACOd expressed in cytosol increased the IA production, while combining the two IA synthetic pathways in cytosol and mitochondria resulted in unchanged IA production. (**F**) The comparison of IA production through *cis*-aconitate and *trans-*aconitate. (**G**) Modulate expression of AtCAD and AtMTT under different promoters and their combination. (**H**) Increasing the copy number of the selected combination resulted in increased IA production. Strains were cultivated in 24–deep well plates after 4 days. Strains were cultivated in 24–deep well plates after 4 days. All data represent the mean of *n* = 3 biologically independent samples, and error bars show SD.

To test whether the earlier *cis*-aconitate enhancing strategy (Fig. 2E) could increase IA production, we introduced a codon optimized *A. terreus* CAD gene in the promising strains in step 1. Blocking lipid accumulation increased IA production from 0.027 to 0.049 g/liter (JFYL013), while blocking the glyoxylate cycle increased production of IA to 0.126 g/liter (Fig. 3A). The codeletion of *ICL1/2* and *IDP* resulted in the highest IA accumulation of 0.184 g/liter in JFYL033, which is consistent with their enhanced *cis*-aconitate accumulation (Fig. 2E). Because several reports showed that subcellular localization of metabolic pathways can efficiently increase product conversion (*47*–*50*), CAD was targeted to the mitochondria by fusing it with the mitochondrial leading sequence of the cytochrome c oxidase subunit 4 (cox4). As anticipated, more IA (0.221 g/liter, JFYL058) was produced with mitochondrial CAD, suggesting more efficient transformation to IA (Fig. 3A).

Both the cytosolic and mitochondrial IA production routes require transporters. For cytosolic IA production, *cis*-aconitate [tricarboxylic acid (TCA)] is transported from the mitochondria to the cytosol. Contrastingly, mitochondrial IA production requires IA (dicarboxylic acid) to be transported from the mitochondria to the cytosol (Fig. 1). Therefore, three mitochondrial TCA transporters (MTTs) and three mitochondrial dicarboxylic acid transporters (MDTs) from different species were expressed under two strong promoters, pTef1 and pTefintron, which has a 17 times higher expression level than pTef1 (*51*). The AtMTT transporter from *A. terreus* under pTef1 promoter resulted in 0.527 g/liter (JFYL014, Fig. 3B) and showed a decreased IA titer with pTefintron promoter (JFYL036), suggesting that extremely high expression of AtMTT is not beneficial for IA production. Expressing MTTs from *Aspergillus kawachii* (AkCTP) and *U. maydis* (UmMTT1) under pTef1 resulted in decreased IA titers (Fig. 3B) compared to JFYL014. On the other hand, pTefintron yielded ca. five times higher IA production, suggesting that these two transporters have low activity. As for MDT transporters, none of the candidates, i.e., *S. cerevisiae* (SceDIC), *Mus musculus* (MumDIC), and *Arabidopsis thaliana* (AthDIC), increased IA production (Fig. 3C). Therefore, the cytosolic pathway was selected for the next studies.

To further enhance AtMTT-facilitated cytosolic IA production, we removed sink pathways. Blocking lipid accumulation yielded higher IA production when expressing CAD and MTT (JFYL014, Fig. 3D), consistent with increased *cis*-aconitate production (JFYL007, Fig. 2E). Unexpectedly, deletion of *ICL1* and *ICL2*, which improved *cis*-aconitate (JFYL018 in Fig. 2E) and IA accumulation (JFYL031 in Fig. 3A) without the AtMTT transporter, resulted in decreased IA production (Fig. 3D), which was further worsened by IDP codeletion. Therefore, only disruption of lipid accumulation (i.e., JFYL014) was retained in all later IA strains. Moreover, overexpression of mitochondrial AtACO, which converts CA to *cis*-aconitate, did not improve IA production (Fig. 3E), while cytosolic overexpression of AtACOd without mitochondrial leading sequence increased the IA titer to 0.696 g/liter. This indicates that considerable amounts of cytosolic CA are transformed to *cis*-aconitate for IA production. Attempts to facilitate substrate channeling using a flexible Glycine-Serine-Glycine (GSG) linker in an AtACO-AtCAD fusion protein did not result in improved production (fig. S5). In addition, overexpression of both mitochondrial and cytosolic AtCAD (JFYL089, Fig. 3E) showed unchanged IA titers compared to sole cytosolic AtCAD.

IA production through *trans*-aconitate as an alternative promising pathway (*52*) derived from *U. maydis* was then tested. No detectable IA accumulation was observed with expression of TAD (UmTad1, Fig. 3F) and is consistent with no detectable levels *trans*-aconitate. Expression of UmAdi1 (JFYL092, Fig. 3F) yielded 0.125 g/liter IA, which seemed very promising compared to the *cis*-aconitate pathway, which only resulted in 0.049 g/liter IA without the AtMTT transporter (JFYL013, Fig. 3A). However, after introducing the AtMTT, production through the *trans*-aconitate pathway was 0.426 g/liter, lower than production through the *cis*-aconitate pathway (0.527 g/liter; Fig. 3F). Therefore, cytosolic AtCAD–based IA production with the AtMTT transporter was chosen as the preferred strain.

The promising IA synthetic pathway with *cis*-aconitate was then fine-tuned by using various promoters and copy numbers of AtMTT and AtCAD (Fig. 3G). It is reported that the expression level of the promoters used in this study are as follows: pTefin > p4UASTef > pGAPDH > pTef > pBR30 and pICL1 (*51*). When introducing AtCAD without AtMTT, we observed that increasing its expression level within a range increased the IA titer (JFYL039 > JFYL066, JFYL010 > JFYL013, Fig. 3G). However, as CAD was reported to be toxic to cells (*52*), we observed a decrease in the IA titer when increase the *AtCAD* expression level (JFYL115 versus JFYL039, JFYL035 versus JFYL010, Fig. 3G). Meanwhile, we also observed that increasing the AtMTT’s expression level separately within a certain level could increase the IA titer (JFYL116 > JFYL040 > JFYL066 > JFYL014, Fig. 3G). However, similarly, when increasing the *AtMTT*’s expression level by more than 10 times with pTefin, a small decrease in the IA titer was observed (JFYL036 versus JFYL 014, Fig. 3B). This might be caused by the characteristic of the transporter of MTT. Therefore, the expression of *AtMTT* and *AtCAD* need to be finely tuned and overexpressed together with the best combination (Fig. 3H). Because the promoter p4UASTef has a complex and repeated structure that might result in genetic instability, the combination of pTef-*AtCAD* and pGPD-*AtMTT* was selected for further studies. When integrated in different genetic loci, this promising combination resulted in similar IA production (Fig. 3H), while a notable increase of IA was achieved introducing multiple copy numbers in JFYL077. Specifically, six copies of pTef-AtCAD and pGPD-AtMTT combination yielded the best IA production. However, further overexpression of AtACOd did not show synergistic effect on IA production (fig. S6), indicating a high flux through *cis*-aconitate.

### Mimicking the regulation of NL

**Fig. 4.**
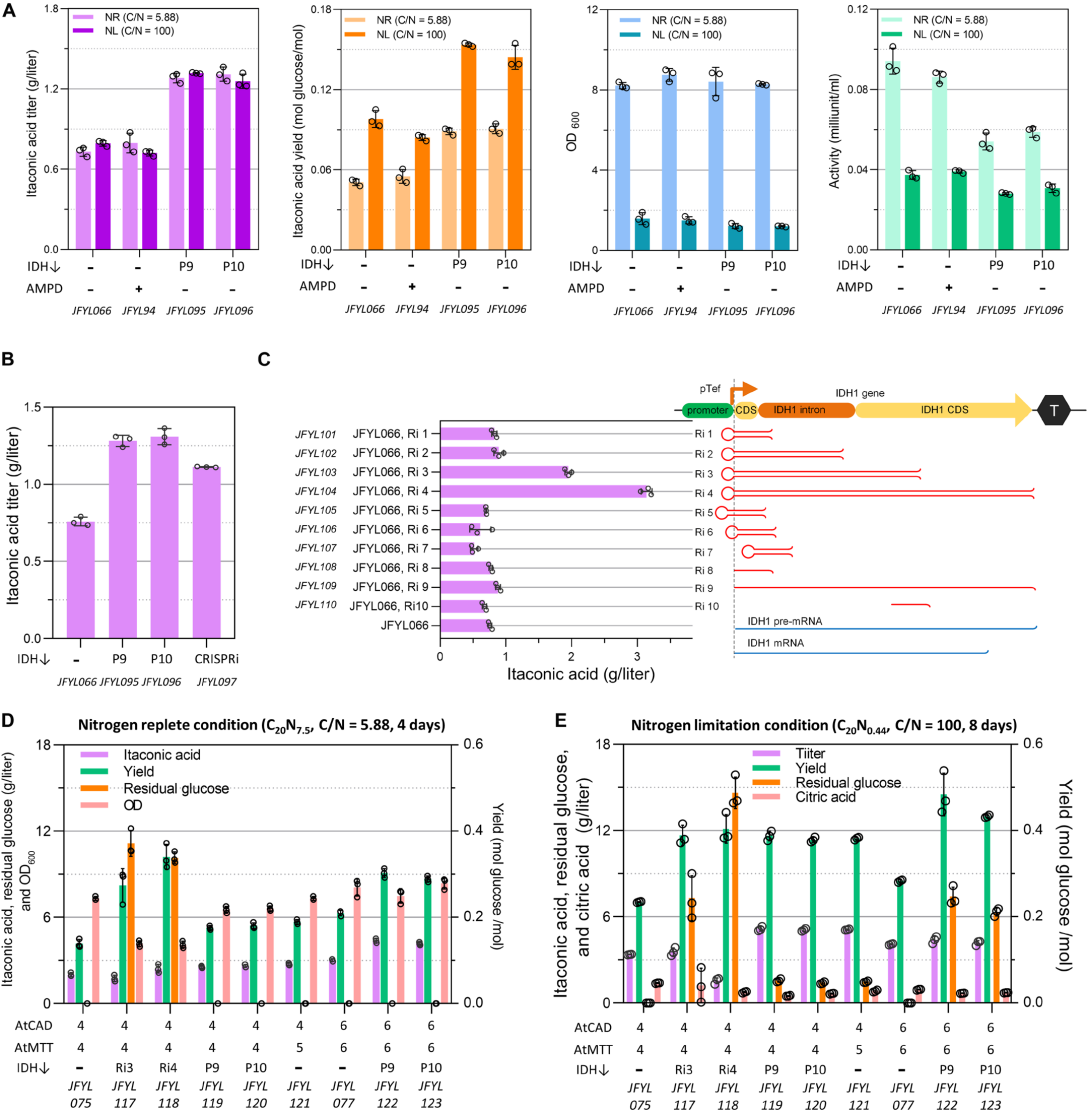
Mimic NL by down-regulating IDH. Strains were cultivated in 24–deep well plates after 4 days. (**A**) The IDH was down-regulated by overexpression of AMPD and changing IDH’s native promoter P9 and P10. The IA titer, yield, OD_600_, and IDH activity were measured. (**B**) Comparison of the effect of IDH down-regulation by promoter changing and CRISPRi. (**C**) The effect of different RNAi types on IA production. (**D**) IA production in an NR condition. (**E**) IA production in an NL condition. All data represent the mean of *n* = 3 biologically independent samples, and error bars show SD.

The IA producer JFYL066 (JFYL007 with one copy number of AtCAD and AtMTT) produced similar IA titer in both the NR and NL conditions (Fig. 4A) when cultivated until glucose depleted (4 and 8 days, respectively). While titers were similar after glucose depletion, the NL condition resulted in higher IA yield but lower productivity, probably due to low biomass formation. It would therefore be convenient to mimic NL condition by imitating part of its regulation in NR conditions, to achieve both high yield (from NL) and productivity (from NR).

NL increases the activity of adenosine monophosphate (AMP) deaminase (AMPD), which converts AMP into inosine monophosphate. IDH, as AMP-dependent dehydrogenase, is inhibited by low AMP levels (*53*, *54*). Inconsistent with previous reports (*33*), overexpression of native AMP deaminase (JFYL094) showed no positive effect on IA production (Fig. 4A). Next, IDH was down-regulated by changing its native promoter to weaker promoters, and 10 candidates were tested on the basis of reported RNA sequencing data (*55*) (fig. S7). Because earlier attempts to delete the IDH open reading frame failed (step 1), obtaining positive transformants proved challenging when the expression level of the promoter was too low. Only two promoters, P9 and P10, were successfully exchanged. The IA titer and yield of these two strains (JFYL095 and JFYL096) increased in both NR and NL conditions compared to JFYL066, while the IDH activity decreased (Fig. 4A). In general, IDH activities and IA yields anticorrelated. However, in the NL conditions, low biomass and IDH activity led to prolonged fermentation time and reduced productivity. However, with down-regulated IDH, IA titer, yield, and productivity all increased in NR conditions (JFYL095 and JFYL096). The IA yields of JFYL095 and JFYL096 in NR condition were similar to JFYL066 in NL condition, indicating that the positive effect of NL on IA production was successfully mimicked in NR condition.

Because down-regulating essential *Y. lipolytica* genes using weak promoters proved challenging, CRISPR interference (CRISPRi) and RNA interference (RNAi) were used to down-regulate IDH. CRISPRi, allowing for sequence-specific gene repression at the DNA level (*56*), was reported to work well in *Y. lipolytica* (*57*, *58*). It is used here by introducing a dCpf1 (FnCpf1 D917A), and four guide RNA (gRNAs) were designed to target the IDH subunit 1 coding gene simultaneously (fig. S8). The resulting strain JFYL097 produced IA (1.12 g/liter) (Fig. 4B), indicating that CRISPRi silencing worked in *Y. lipolyti*ca. However, the IA titer was still lower than those with weak promoter exchanged (Fig. 4B). Alongside, we established RNAi in *Y. lipolytica* (Fig. 4C and fig. S9) by introducing codon optimized *Dcr1* (Dicer) and *Argo1* (Argonaute) of *Saccharomyces castellii* (*59*). After exploring different silencing strengths by varying the length of short hairpin RNA (shRNA) or antisense single-stranded RNA (fig. S9 and table S7), strains with the longest shRNA (Ri3 and Ri4, Fig. 4D), which would be expected to have the strongest silencing, increased IA titer by 157 and 314% (1.95 and 3.14 g/liter), respectively. These results indicate that the RNAi targeting IDH worked and could greatly increase the IA titer. The gene expression level of IDH was decreased (fig. S10) for the IDH down-regulated strains, and the decreased IDH activity was observed by IDH assay (fig. S11).

The IDH down-regulation strategy was then combined with modulation of AtCAD and AtMTT from step 2. JFYL117 and JFYL118 produced higher IA yields than in JFYL075 (Fig. 4E). Unfortunately, because high glucose concentrations were left in the RNAi strains, JFYL118 showed lower IA titer (Fig. 4D). Meanwhile, JFYL119 and JFYL120 produced similar IA titers but lower yields due to higher glucose depletion compared with JFYL118. Moreover, increasing the copy number of AtCAD and AtMTT to six copies could further increase the IA titer and yield, and JFYL122 resulted in the highest titer (4.3 g/liter IA) with a yield of 0.31 mol IA/mol glucose. When NL condition (Fig. 4E) was used, all strains exhibited much higher IA yields but lower productivities. IA with similar titer and yield was produced by JFYL122 in NR condition (4 days) and JFYL077 in NL condition (8 days), indicating that we successfully mimicked the NL and enhanced productivity by down-regulation of IDH and increased the productivity by twofold. Therefore, JFYL122 with six copies of AtCAD and AtMTT and P9 promotor modulated IDH expression was selected for further study because of its superior titer in NR conditions and highest yield in NR conditions, compared with all IDH down-regulated strains.

**Fig. 5.**
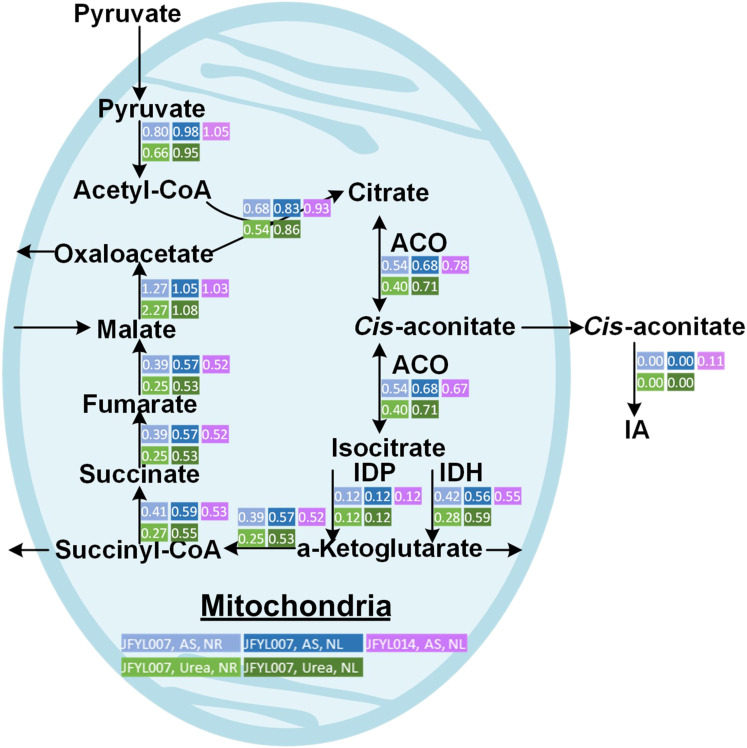
FBA of JFYL007 and JFYL066 in NR and NL conditions. Strains were cultivated in modified Delft medium with ammonia sulfate or urea in NR or NL conditions in chemostat. All data represent the mean of *n* = 3 biologically independent samples.

To present the different flux in NL and NR conditions, we performed flux balance analysis (FBA) by constraining with measured exchange reaction rates and modified biomass compositions from chemostat bioreactor cultivations (Fig. 5). The predicted flux in JFYL007 indicated that the flux of TCA is higher from NL condition than that from NR condition. The higher flux could increase *cis*-aconitate availability, which contributes to high IA yield. Therefore, our strategy to decouple NL and high flux in TCA by mimicking NL regulations, allowing higher biomass, can result in balanced IA production with both high yield and productivity. Notably, this simulation especially contributes to IA production when NL should be avoided in step 4 in the next section.

### Fermentation engineering and scale-up

**Fig. 6.**
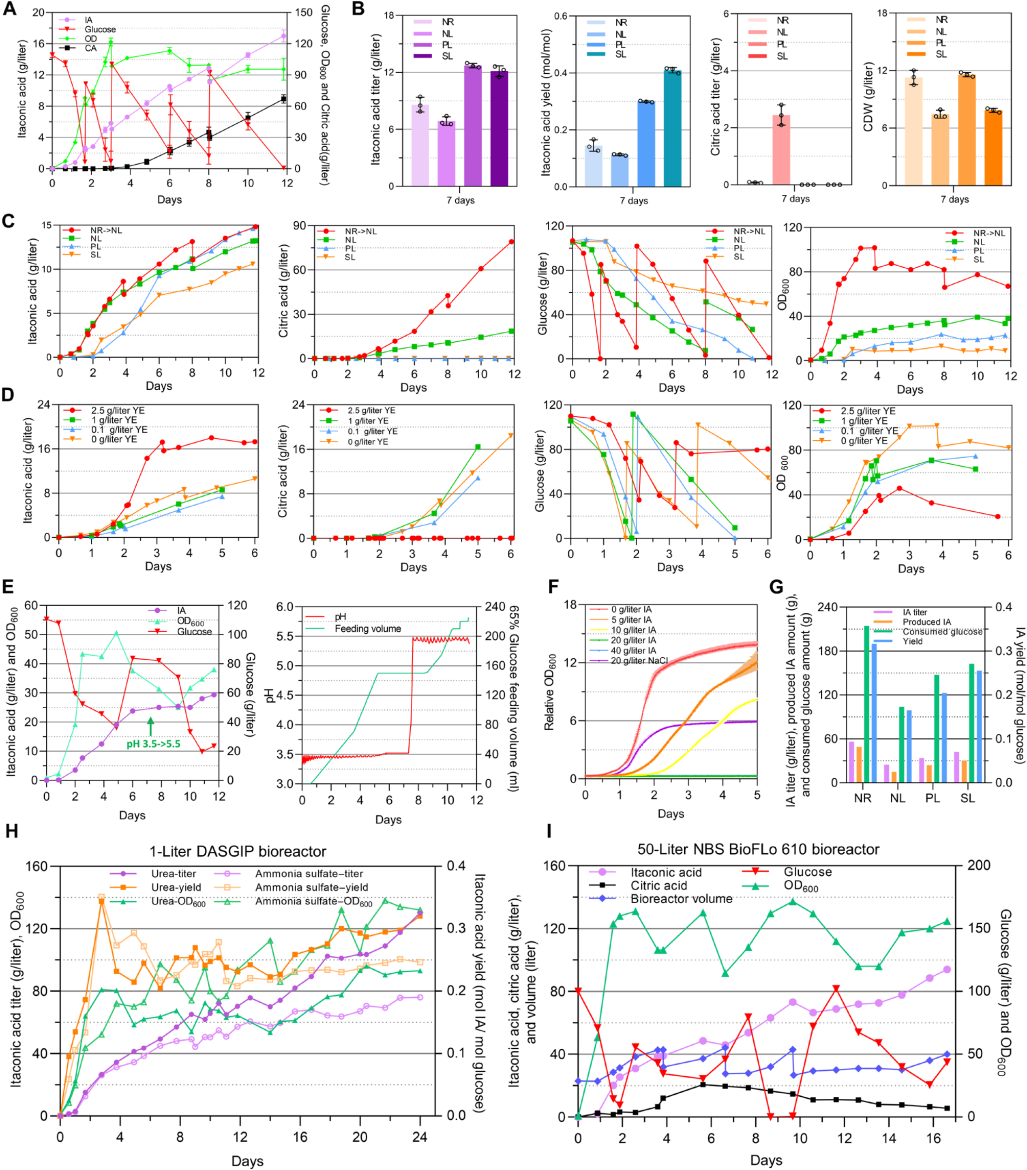
Enhanced IA production. (**A**) JFYL122 was cultivated in 1-liter bioreactors (initial C/N = 22, 65% glucose was fed in pulse form). Data represent the mean of *n* = 2 biologically independent samples, and error bars show SD. (**B**) JFYL121 was cultivated in flasks under NR (C_100_N_10_), NL (C_100_N_2.5_), PL, and SL conditions. All data represent the mean of *n* = 3 independent samples. (**C**) JFYL122 was cultivated in 1-liter bioreactors under nitrogen switch (initial C_100_N_10_, from NR to NL), NL (C_100_N_2.5_), PL, and SL conditions. (**D**) The effect of addition of YE in C_100_N_10_ (C/N = 22) Delft medium. (**E**) Enhanced IA production with continuous feeding of glucose and YE. The pH was changed from 3.5 to 5.5. (**F**) IA tolerance measured in BioLector. (**G**) JFYL122 was cultivated in 1-liter bioreactors under NR, NL, PL, and SL conditions with the continuous feeding of glucose and YE together (pH at 5.5). (**H**) JFYL122 was cultivated in 1-liter bioreactor, and AS and urea were used as the nitrogen source, respectively. (**I**) JFYL122 was cultivated in 50-liter bioreactor at semipilot scale. Data for (B) and (F) represent the mean of *n* = 3 biologically independent samples and error bars show SD. The cultivation in 50 liters was carried out as a single experiment, and all other bioreactor cultivations were carried out in two replicates. The presented cultivations depict a single representative cultivation for (C), (D), (E), (G), (H), and (I).

The most promising strain (JFYL122) was cultivated in 1-liter fed-batch bioreactors. We tested a widely used nitrogen control strategy (NR switch, NR -> NL) in which high biomass is first generated under NR condition (C/N = 22), and NL conditions were then generated by nitrogen source consumption during biomass accumulation. Unexpectedly, large amounts of CA started to accumulate after 3 days (Fig. 6A), and surpassed the IA titer, with a final titer of 67.5 and 17.1 g/liter, respectively. The predominant CA accumulation (Fig. 6A) was possibly due to complex regulation where both absolute nitrogen amount and C/N ratio affect metabolism. While glucose was fed during the cultivation, nitrogen likely became limiting at day 3, when growth halted at optical density at 600 nm (OD_600_) 120. At this point, abundant CA started to be secreted, indicating that it was not being converted to *cis*-aconitate. This result contrasts with what we measured before in deep-well plates, where only ca. 1 g/liter CA was produced in NL condition (Fig. 4E). As scale-up conditions were not directly transferable, further fermentation engineering is required to optimize production in bioreactors.

To reduce CA accumulation, we tested PL, and SL. JFYL122 was grown on different media in shake flasks with higher glucose concentration (100 g/liter) than previously tested (20 g/liter glucose; Fig. 4, D and E). CA only accumulated if nitrogen was the limiting nutrient (NL-bioreactor medium), while both PL and SL gave higher IA titers and yields (Fig. 6B). These conditions were further tested in bioreactors (Fig. 6C), and CA was not detected under PL and SL. These conditions showed higher IA yields but lower glucose consumption rate (fig. S12). These results indicate that NL should be avoided to prevent CA secretion. This can be achieved by feeding nitrogen to support higher OD_600_ and avoiding alternative limiting nutrients.

To increase IA production, we also tested the following conditions. Yeast extract (YE), having low production cost, is frequently used to improve cell growth (*60*) and to increase productivity. Different concentration of YE were tested in NL-bioreactor medium. YE (2.5 g/liter) largely increased the IA titer to 17.3 g/liter (Fig. 6D) within 3 days. With lower YE supplementation, CA accumulated from day 2, indicating depletion of the nitrogen source. Furthermore, high osmotic pressure is reported as a key factor responsible for inhibition of yeast growth and decrease fermentation performance (*61*). Therefore, changing from pulsed to continuous feeding could reduce potential osmotic stress effects. Switching from pulsed to continuous feed increased IA production to 24.5 g/liter on the sixth day (Fig. 6E). However, afterwards, cells arrested glucose consumption and IA production. Cells growth and glucose consumption recovered when the pH was increased from 3.5 to 5.5, and IA production resumed (Fig. 6E). Testing IA tolerance in high-throughput microbioreactors (BioLector) with an initial pH of 3.5 showed that 20 g/liter IA interrupts cell growth (Fig. 6F), while it was reported that, at pH 7, *Y. lipolytica* is tolerant to 60 g/liter IA (*34*). Therefore, the addition of YE, continuous feeding, and pH of 5.5 were the key factors in later cultivations.

These optimized conditions were combined in 1-liter fed-batch cultivation: The pH was controlled at 5.5, and glucose (600 g/liter) and YE (20 g/liter) were continuously fed. No growth disruption was observed when IA titer exceeded 25 g/liter (Fig. 6G). Higher IA production was observed under PL and SL than NL condition (Fig. 6G). Notably, NR condition increased IA productivity, titer, and yield, indicating that the growth-associated production strategy with nitrogen feeding and mimicking IDH down-regulation works well. This condition was further tested in bioreactors, and 68.1 g/liter IA was produced in 16.75 days (Fig. 6H), using ammonium sulfate as nitrogen source. However, to maintain the pH at 5.5, large volumes of base (6 M KOH) were required (fig. S13) because of the acidification. This drastically increased the cultivation volume, lowering the IA titer after 17 days, although the absolute amount of IA still increases (fig. S13). Ammonium sulfate consumption reduces the pH, while using urea, an alternative and cheap nitrogen source (that does not act as carbon source for *Y. lipolytica*), will not acidify the medium (*62*). Thus, using urea as nitrogen source reduced the base feeding volumes and considerably increased IA titer to 130.5 g/liter IA with a yield of 0.320 mol/mol glucose. These results suggested the successful establishment of our engineered strain as a promising candidate for IA production (table S1 and S2).

To verify performance during the scale-up at a semipilot scale, we cultivated the strain JFYL122 in a 50-liter bioreactor using urea as the nitrogen source. Within 2 days, the OD_600_ quickly rose to 160, and we obtained the highest productivity (0.566 g/liter per hour). Because of accidental glucose limitation between days 8 and 9, the cells lost their vitality. After the glucose limitation was removed, the fermentation took an additional 3 days to recover. However, IA production continued afterwards with high productivity. A final titer of 94.1 g/liter IA with a productivity of 0.238 g/liter per hour and a yield of 0.36 mol/mol glucose were reached after day 16.58. The total amount of IA produced during this fermentation was 6161.9 g. These results indicate that our engineered strain JFYL122 exhibited robustness during the scale-up process, making it a promising IA producer at industrial-scale fermentation.

## DISCUSSION

IA enables the synthesis of many valuable bio-based products with remarkable properties and exhibits potential applications in the biomedical field. Until recently, commercial IA bioproduction was limited to *A. terreus* and *U. maydis*, but industrial-scale production remains challenging. In our work, we successfully reprogrammed the GRAS microorganism *Y. lipolytica* for enhanced IA production.

During strain development, some strategies that showed positive effects on IA production were either not beneficial or lacked a synergistic effect when combined. While this is common in strain engineering and can be attributed to the complexity of *Y. lipolytica* metabolism, it posed challenges in enhancing and scaling up IA production. We carefully adjusted three positive strategies to balance growth, titer, yield, and productivity. (i) For the ICA sink node, deletions of both *ICL1* in glyoxylate cycle, *ICL2* in methylcitrate cycle, and NADP^+^-dependent IDP in the TCA cycle increased *cis*-aconitate availability and IA titer at low IA concentrations. However, IA titer decreased after introducing the AtMTT transporter (Fig. 3, A and D), indicating that synergistic effects should be evaluated during strain development. Moreover, there is conflicting information about the function and location of ICL2 (*63*, *64*), so the effect of *ICL2* deletion on IA production needs further study. (ii) IA production inside the mitochondria was promising compared to cytosolic IA production; however, no suitable IA transporter was found to further enhance IA production. The IA synthetic pathway through *trans*-aconitate was likely dominant at low *cis*-aconitate and IA levels. After introducing AtMTT, JFYL093 (trans pathway) produced less IA than JFYL014 (cis pathway), possibly because of a two-step conversion and low-kinetics parameters of TAD, while AtCAD exhibited a high catalytic rate constants (*k*_cat_) and Michaelis constant (*K*_M_) (*65*). It was also previously reported that trans pathway produced lower IA amount at high IA level due to unused substrate (*52*). (iii) RNAi for IDH exhibited a considerable effect on IA production (Fig. 4, C and D). However, the biomass was relatively low, and there was considerable residual glucose (Fig. 4D). As decreased IDH activity resulted in reduced glucose consumption rate, a weaker promoter, P9, was selected as it performed best in balancing cell growth and IA productivity. Notably, weak promoter changing in *Y. lipolytica* sometimes is very time consuming, as it was very hard to obtain positive transformants when the expression level of the promoter was too low. However, RNAi established here could provide much easier and even deeper down-regulation levels. Further study of RNAi by using different lengths of double-stranded RNA of the whole gene or CRISPRi by testing different targeting sites are needed for fine turning of down-regulation of gene expression levels.

NL condition resulted in high IA yield but lower productivity. NR condition not only increased IA titer and productivity but also contributed to IA production during scale-up. To increase IA yield in NR conditions, we successfully mimicked the NL regulation by down-regulating IDH activity with the present of nitrogen source. This approach increased IA yield, retaining high productivity in NR conditions. Furthermore, in NL conditions, down-regulating IDH indeed increased IA yield (Fig. 4, A, D, and E), suggesting a deeper repression than the native regulations in NL. Therefore, the technology developed here can serve as a universal platform for CA or lipid production in *Y. lipolytica* when NL condition is preferred. It is worth noting that, in addition to conventional deletion and promoter replacement, RNAi and CRISPRi were successfully used to enable the fine modulation of gene expression levels, especially for essential genes like IDH. To the best of our knowledge, RNAi in *Y. lipolytica* has not been previously reported. These two gene knockdown technologies developed here are promising approaches for fine modulation of gene expression levels and expands the current genetic toolbox for *Y. lipolytica* engineering.

During the scale-up process we had to address three main challenges that were absent on flask level (excess of CA secretion under NL, IA tolerance, and excess feeding of base). (i) There are two potential reasons for the increased CA during the scale-up from previous reports. First, growth and CA production are all known to be affected by oxygen availability (*66*). The increased dissolved oxygen level by agitation and aeration could result in increased CA accumulation. Second, it was reported that under low iron concentrations, iron is a factor limiting the activity of the iron-dependent enzyme, ACO (aconitate hydratase; EC 4.2.1.3) (*67*). Decreased activity of ACO, caused by the iron consumption by higher biomass in bioreactors, could result in blocked flux form CA to *cis*-aconitate, and therefore, more CA could be detected in the bioreactor run. Further studies of oxygen levels and iron concentrations could be investigated to further increase IA production. In this study, CA secretion can be prevented by avoiding NL condition. It was reported that NL also resulted in decreased IA titer in *A. terreus* (*23*, *68*). However, avoiding NL conditions can decrease yields. This can be mitigated by IDH knockdown (Fig. 4, D and E, JYFL077 and JFYL122). Furthermore, to reduce excess CA secretion, we compared four limiting conditions (NL switch, NL, PL, and SL) in both flasks and bioreactors (Fig. 6, B and C). CA was reported to accumulate in all NL, PL, and SL conditions, while in our study, CA secretion was totally abolished in PL and SL, which increased IA yield. However, because of low productivity, all nutrient limitations were avoided in this study. (ii) Increasing pH to 5.5 and NR condition enhanced the IA tolerance in our engineered *Y. lipolytica* strains. It was reported that *Y. lipolytica* is highly tolerant to IA at pH 7 and can grow with 60 g/liter IA (*34*). The p*K*_a_ values (where *K*_a_ is the acid dissociation constant) of IA for its two dissociation modules, are 3.84 and 5.55 (25°C), and most IA at pH 7 is in the ionic form, which might show less toxicity. In our study, cell growth was inhibited at pH 3.5 and 25 g/liter IA, as most of IA is in the acid form. This can be solved by increasing the pH to 5.5 to increase the ratio of IA ionic type (Fig. 6E). Furthermore, IA toxicity might be alleviated by the presence of nitrogen source. It was previously reported that the growth of *A. niger* was hampered with 10 g/liter IA, but it was shown that the IA toxicity could be alleviated by maintaining nitrogen availability (*32*). This can be explained by the glutamate-dependent acid resistance mechanism (*31*), which is used by acid-tolerant microorganisms to reduce acid toxicity by increasing the alkalinity of the cytoplasm (*31*). Notably, our strategy of mimicking NL regulations to keep the nitrogen source availability might contribute the enhanced IA tolerance. (iii) Switching nitrogen source from ammonium sulfate to urea reduced the fed base volume and increased IA production. To maintain pH at 5.5 while using ammonium sulfate, an excess base (6 M KOH) was fed, and the volume increased greatly. The medium had to be removed to avoid exceeding the maximum working volume. In our previous study, we showed that urea is a drop-in nitrogen source alternative to ammonia sulfate in *Y. lipolytica*, and there are no concerted changes in the transcriptome (*62*). Although the biomass decreased when using urea as the nitrogen source, the IA titer and yield both increased compared to ammonium sulfate (Fig. 6H). Notably, without down-regulation of IDH, the FBA shows that, in NR, the flux through *cis*-aconitate by using urea is lower than that using ammonium sulfate, while in NL, the *cis*-aconitate flux is similar between urea and ammonium sulfate (Fig. 5). However, by mimicking the regulation of NL, the IA titer, yield, and productivity are higher when using urea (Fig. 6H) instead of ammonium sulfate.

In this study, we used a modular approach to gradually reprogram the metabolism of *Y. lipolytica* to improve IA production from 0.027 to 130.5 g/liter. We successfully managed to mimic the NL regulations in NR conditions by down-regulating IDH in *Y. lipolytica*. The NR condition increased IA titer, yield, and productivity, indicating that the growth-associated production strategy with nitrogen feeding and mimicking IDH down-regulation works well. This has interesting implications for lots of other products commonly made in *Y. lipolytica* and other oleaginous species. Beyond offering insights into the metabolism of *Y. lipolytica*, our findings present a wide range of synthetic biology strategies for reconstructing and modulating heterologous pathways in microbial cell factories. With successful optimization during the scale-up process, the engineered strain JFYL122 achieved an unprecedented titer of 130.5 g/liter in 1-liter bioreactors and 94.8 g/liter at a semipilot scale, suggesting our engineered *Y. lipolytica* as a competitive IA producer. Given the increasing interest in harnessing *Y. lipolytica* as a workhorse, the strategies described in this study provide practical approaches to harness the GRAS *Y. lipolytica* for competitive industrial-scale production.

## METHODS

### Strains, media, and cultivation

The *E. coli* strain DH5α was used for plasmid construction, which was grown at 37°C with shaking in Luria-Bertani broth (Sigma) supplemented with ampicillin (50 μg/ml) for plasmid propagation. All the *Y. lipolytica* strains constructed in this study were derived from ST6512, which is a W29 background strain (Y-63746 from the ARS Culture Collection, Peoria, USA; a.k.a. ATCC20460/CBS7504) and harbors *Cas9* at *KU70* locus for marker-free genomic engineering by an EasyClone YALI toolbox (*51*). The complete list of strains used in this study are listed in table S3.

*Y. lipolytica* strains were cultivated in the minimal mineral, a modified Delft medium (*69*) with 0.2 M phosphate buffer, in shake flasks and 24–deep well plates unless stated otherwise. The NR medium contained (NH_4_)_2_SO_4_ (7.5 g/liter), KH_2_PO_4_ (19.2 g/liter), K_2_HPO_4_ (10.2 g/liter), MgSO_4_•7H_2_O (0.5 g/liter), 2 ml of trace metal solution stock, and 1 ml of vitamin solution stock. The trace metals and vitamin solution were added after autoclave. Trace metal solution contained FeSO_4_•7H_2_O (3.0 g/liter), ZnSO_4_•7H_2_O (4.5 g/liter), CaCl_2_•2H_2_O (4.5 g/liter), MnCl_2_•4H_2_O (1 g/liter), CoCl_2_•6H_2_O (300 mg/liter), CuSO_4_•5H_2_O (300 mg/liter), Na_2_MoO_4_•2H_2_O (400 mg/liter), H_3_BO_3_ (1 g/liter), KI (100 mg/liter), and Na_2_EDTA•2H_2_O (19 g/liter). The vitamin solution stock contained d-biotin (50 mg/liter), d-pantothenic acid hemicalcium salt (1.0 g/liter), thiamin-HCl (1.0 g/liter), pyridoxin-HCl (1.0 g/liter), nicotinic acid (1.0 g/liter), 4-aminobenzoic acid (0.2 g/liter), and myo-inositol (25 g/liter). The initial pH was set at 6.5 with KOH. C*_x_*N*_y_* was used here to indicate the glucose and nitrogen (ammonia sulfate or urea) amount (grams per liter) with *x* and *y* values. C/N was used to indicate the carbon and nitrogen molar ratio. In steps 1 to 3, 20 g/liter glucose and 7.5 g/liter ammonia sulfate were used in this medium to set the NR condition (C/N = 5.88), while in step 3, NL condition (C/N = 100) was achieved with 20 g/liter glucose and 0.44 g/liter ammonia sulfate. In step 4, the media with varying C/N ratios, PL, and SL conditions were provided in table S8 to study the carbon flux distribution between CA and IA for optimal IA fermentation.

In chemostat cultivations, all modified Delft media contained KH_2_PO_4_ (3 g/liter), MgSO_4_•7H_2_O (0.5 g/liter), 1 ml of trace metal solution stock, and 1 ml of vitamin solution stock. For the NL condition (C/N ratio 116), the media contained 0.471 g/liter ammonium sulfate or 0.213 g/liter urea (sterile filtered) and 25 g/liter glucose, while for the NR condition (C/N ratio 3), the media contained 5.28 g/liter ammonium sulfate or 2.4 g/liter urea (sterile filtered) and 7.92 g/liter glucose. pH was set at 5.5 with 2 M KOH, and the temperature was set at 28°C.

All *Y. lipolytica* strains were cultivated at 30°C for shake flask and 24–deep well plate fermentation with a shaking speed of 220 rpm. The 24–deep well plates are purchased from Enzyscreen (plates, CR1424a; lips, CR1224f), and the volume of culture is 2.5 ml.

### Cloning and transformation procedures

Plasmids for genome engineering were constructed using a set of vectors from EasyCloneYALI as backbones (*51*). The construction of plasmid for genome engineering was performed according to EasyCloneYALI instructions. The complete list of plasmids used in this study are listed in table S4. All primers are listed in table S5.

For the gene deletion of ARE1, DGA1, DGA2, LRO1, ICL1, ICL2, IDH1, IDH2, and IDP, equal amounts of two single-stranded oligonucleotides (90 to 120 bp) were used to obtain the repair templates. The pair of oligonucleotides were incubated for 5 min at 98°C and allowed to cool down to room temperature. The EasycloneYALI toolbox was used to construct gRNA plasmids for CRISPR-Cas9 aided gene deletion.

For gene overexpression, the integration plasmids were constructed with different promoters and genes. The codon optimized genes are listed in table S6. The marker-free integration cassettes were obtained by digesting the corresponding plasmids. After purification, around 1 μg of repair fragment was used for transformation. For each transformation, around 500 ng of gRNA plasmid was used.

For IDH1 promoter change, the repair fragments were obtained by overlap polymerase chain reaction (PCR) of the upstream of IDH1, promoter candidate, and downstream of IDH1. AKEC8 was used as the gRNA helper plasmid for IDH1 promoter change.

### Establishment of RNAi

The design was illustrated in fig. S9 based on a previous report (*59*). The codon optimized *Dcr1* under pTef and *Ago1* under pGAPDH are listed in table S6. The Ri1 to Ri10 are listed in table S7.

### Chemostat experiments

Chemostat cultivations were conducted at 28°C using DasGip systems with 1-liter stirrer-pro vessels (Eppendorf) as previously reported (*45*). Briefly, the working volume was 500 ml kept constant using an overflow pump, and pH was maintained at 5.0 ± 0.1 by automatic addition of 2 M KOH. The agitation was set at 600 rpm, and aeration was maintained with sterile air at 30 liters per hour (1 vvm) and monitored with dissolved oxygen (DO) probes (Mettler). Delft medium was used in batch, and constant feed with a dilution rate of 0.10 hour^−1^ was initiated after the exponential growth phase to obtain steady-state cultivation. The growth was monitored by the CO_2_ exhaust gas. Samples for lipid analysis from JFYL007 and OKYL029 were taken after five residence times of steady-state growth. Each condition was cultivated at least in triplicate.

### Lipid body visualization

Lipid body visualization was performed by microscopic analysis. The Bodipy Lipid Probe (2.5 mg/ml in ethanol, Invitrogen) was added to the cell suspension (OD_600_ of around 1) with 10-min incubation at room temperature. A Zeiss Axio Imager M2 microscope (Zeiss, Le Pecq, France) with a 100× objective and Zeiss filters 45 and 46 for fluorescence microscopy were used to acquire the images.

### Lipid extraction and quantification

Samples from OKYL029 and JFYL007 chemostat were harvested for measuring intracellular lipid concentration, lipid ratio. Fatty acids were extracted, transformed to fatty acid methyl esters (FAMEs), and quantified by gas chromatography–mass spectrometry (GC-MS) using a published method. GC-MS (Thermo Fisher Scientific Trace 1310 coupled to a Thermo Fisher Scientific ISQ LT) and a ZBFAME column (Phenomenex, 20 m, 0.18-mm inner diameter, 0.15-μm film thickness) were used for the quantification. The fatty acids (C16:0, C16:1, C18:0, C18:1, and C18:2) were considered to calculate intracellular FAME amount, intracellular lipid concentration (total FAME amount per cell dry weight, mg fatty acid/mg dry biomass).

### High-performance liquid chromatography analysis

Glucose and other extracellular metabolites in medium were quantified by high-performance liquid chromatography (HPLC) UltiMate 3000 HPLC system (Dionex). Fermentation samples were harvested (samples with high OD from bioreactors were diluted 10 times) and centrifuged for 2 min at 13000*g*, and the supernatant was used for HPLC analysis. Two HPLC systems were used. Luna 5μ C18 (*2*), 250*4.6 mm (Phenomenex) was used for testing *cis*-aconitate. Its mobile phase was composed of 0.02 mM monopotassium phosphate buffer (pH 2.65) to methanol (a ratio of 95:5) with a flow rate of 1 ml/min. An Aminex HPX-87H ion exclusion column (Bio-Rad, Solna, Sweden) was used for testing glucose, IA, CA and other metabolites. H_2_SO_4_ (5 mM) was used as mobile phase with a flow rate of 0.6 ml/min. Glucose was quantified using a refractive index detector (Shodex, Munich, Germany), and other metabolites were quantified using a UV detector.

### Quantitative PCR experiment

Strains were cultivated in Delft medium [(NH_4_)_2_SO_4_ (7.5 g/liter) and glucose (20 g/liter)] at 30°C, 250 rpm. For RNA purification, RNA was isolated from yeast culture grown to an OD_600_ of 1.5 ± 0.1 using a RiboPure Yeast kit (Invitrogen). RNA was quantified by a NanoDrop spectrophotometer (Thermo Fisher Scientific), and cDNA was generated from each RNA prep using a High-Capacity cDNA Reverse Transcription Kit (Applied Biosystems). cDNA (10 ng) was used for each quantitative PCR (qPCR). qPCR results were normalized to the housekeeping gene glyceraldehyde-3-phosphate dehydrogenase (GAPDH). All qPCR primers were designed manually using Benchling. All qPCRs were performed in a StepOnePlus Real-Time PCR System (Applied Biosystems) using SYBR Green JumpStart Taq ReadyMix (Sigma-Aldrich). The ΔΔCt method is used to calculate the relative fold gene expression (*70*).

### IDH assay

The IDH enzyme activity was determined using the IDH activity detection kit (no. MAK062, Sigma, USA), according to the manufacturer’s instruction of the assay kit. Cells were harvested at 48 hours, and the homogenates obtained after the cell disintegration with the glass beads were used for IDH assay.

### FBA analysis

The *Y. lipolytica* genome-scale model (GEM) used in this work were refined on the basis of a published GEM named iYali v4.1.1 (*71*), during which the flux bounds for reactions y102884, y102948, y000303, y002305, y000659, and y000661 were constrained at zero to reflect the gene deletion and the manual protein compartment annotation. In addition, four reactions were added into GEMs to represent the whole molecular process in synthesis and secretion of itaconate. During the simulation using a python package cobrapy, the mean flux values of the physiological parameters, including the growth rate, production rate and by-product formation rate, were used to constrain GEMs. Afterwards, the minimization of glucose uptake rates was set as the optimizing objective function to solve the fluxes through each reaction based on the Parsimonious FBA procedure (*72*).

### Bioreactor fermentations

Bioreactor fermentations at benchtop level were performed in DasGip 1-L stirrer-pro vessels (Eppendorf, Jülich, Germany) at 30°C. pH was monitored with a pH sensor (Mettler Toledo, Switzerland) and maintained at a set value by automatic addition of 6 M KOH or 2 M HCl. Bioreactor fermentations at semipilot level were performed in New Brunswick BioFlo 610 bioreactor systems. The media are shown in table S8.

### IA tolerance experiment

The modified Delft medium, with the addition of MES to a final concentration of 0.2 M to reduce the influence of pH change, was supplemented with various concentrations of IA (0, 5, 10, 20, and 40 g/liter) or NaCl (20 g/liter) as a negative control to test the IA tolerance. The initial pH was 6.5. The strains were cultured in 48–deep well plates in microbioreactors BioLector (m2p-labs) at 30°C with the initial OD_600_ of 0.2. The OD_600_ was measured every 30 min automatically.

## References

[1] N. Devi, S. Singh, S. Manickam, N. Cruz-Martins, V. Kumar, R. Verma, D. Kumar, Itaconic acid and its applications for textile, pharma and agro-industrial purposes. Sustainability 14, 13777 (2022).

[2] T. Werpy, G. Petersen, “Top value added chemicals from biomass: Volume I--results of screening for potential candidates from sugars and synthesis gas” (National Renewable Energy Laboratory, 2004). https://doi.org/10.2172/15008859.

[3] B. E. Teleky, D. C. Vodnar, Biomass-derived production of itaconic acid as a building block in specialty polymers. Polymers 11, 1035 (2019). 31212656 10.3390/polym11061035 PMC6630286

[4] T. Robert, S. Friebel, Itaconic acid–A versatile building block for renewable polyesters with enhanced functionality. Green Chem. 18, 2922–2934 (2016).

[5] B. Guo, Y. Chen, Y. Lei, L. Zhang, W. Y. Zhou, A. B. Rabie, J. Zhao, Biobased poly(propylene sebacate) as shape memory polymer with tunable switching temperature for potential biomedical applications. Biomacromolecules 12, 1312–1321 (2011). 21381645 10.1021/bm2000378

[6] D. M. Raţă, J.-F. Chailan, C. A. Peptu, M. Costuleanu, M. Popa, Chitosan: Poly(N-vinylpyrrolidone-alt-itaconic anhydride) nanocapsules—A promising alternative for the lung cancer treatment. J. Nanopart. Res. 17, 316 (2015).

[7] M. M. Babić, B. Đ. Božić, B. Đ. Božić, J. M. Filipović, G. S. Ušćumlić, S. L. Tomić, Evaluation of novel antiproliferative controlled drug delivery system based on poly(2-hydroxypropyl acrylate/itaconic acid) hydrogels and nickel complex with Oxaprozin. Mater. Lett. 163, 214–217 (2016).

[8] D. Boschert, A. Schneider-Chaabane, A. Himmelsbach, A. Eickenscheidt, K. Lienkamp, Synthesis and bioactivity of polymer-based synthetic mimics of antimicrobial peptides (SMAMPs) made from asymmetrically disubstituted itaconates. Chemistry 24, 8217–8227 (2018). 29600579 10.1002/chem.201800907 PMC7611503

[9] S. K. Bajpai, P. Jyotishi, M. Bajpai, Synthesis of nanosilver loaded chitosan/poly(acrylamide-co-itaconic acid) based inter-polyelectrolyte complex films for antimicrobial applications. Carbohydr. Polym. 154, 223–230 (2016). 27577913 10.1016/j.carbpol.2016.08.044

[10] A. Michelucci, T. Cordes, J. Ghelfi, A. Pailot, N. Reiling, O. Goldmann, T. Binz, A. Wegner, A. Tallam, A. Rausell, M. Buttini, C. L. Linster, E. Medina, R. Balling, K. Hiller, Immune-responsive gene 1 protein links metabolism to immunity by catalyzing itaconic acid production. Proc. Natl. Acad. Sci. U.S.A. 110, 7820–7825 (2013). 23610393 10.1073/pnas.1218599110 PMC3651434

[11] C. L. Strelko, W. Lu, F. J. Dufort, T. N. Seyfried, T. C. Chiles, J. D. Rabinowitz, M. F. Roberts, Itaconic acid is a mammalian metabolite induced during macrophage activation. J. Am. Chem. Soc. 133, 16386–16389 (2011). 21919507 10.1021/ja2070889 PMC3216473

[12] M. Bambouskova, L. Gorvel, V. Lampropoulou, A. Sergushichev, E. Loginicheva, K. Johnson, D. Korenfeld, M. E. Mathyer, H. Kim, L. H. Huang, D. Duncan, H. Bregman, A. Keskin, A. Santeford, R. S. Apte, R. Sehgal, B. Johnson, G. K. Amarasinghe, M. P. Soares, T. Satoh, S. Akira, T. Hai, C. de Guzman Strong, K. Auclair, T. P. Roddy, S. A. Biller, M. Jovanovic, E. Klechevsky, K. M. Stewart, G. J. Randolph, M. N. Artyomov, Electrophilic properties of itaconate and derivatives regulate the IκBζ–ATF3 inflammatory axis. Nature 556, 501–504 (2018). 29670287 10.1038/s41586-018-0052-z PMC6037913

[13] E. L. Mills, D. G. Ryan, H. A. Prag, D. Dikovskaya, D. Menon, Z. Zaslona, M. P. Jedrychowski, A. S. H. Costa, M. Higgins, E. Hams, J. Szpyt, M. C. Runtsch, M. S. King, J. F. McGouran, R. Fischer, B. M. Kessler, A. F. McGettrick, M. M. Hughes, R. G. Carroll, L. M. Booty, E. V. Knatko, P. J. Meakin, M. L. J. Ashford, L. K. Modis, G. Brunori, D. C. Sevin, P. G. Fallon, S. T. Caldwell, E. R. S. Kunji, E. T. Chouchani, C. Frezza, A. T. Dinkova-Kostova, R. C. Hartley, M. P. Murphy, L. A. O’Neill, Itaconate is an anti-inflammatory metabolite that activates Nrf2 via alkylation of KEAP1. Nature 556, 113–117 (2018). 29590092 10.1038/nature25986 PMC6047741

[14] N. Wierckx, G. Agrimi, P. S. Lubeck, M. G. Steiger, N. P. Mira, P. J. Punt, Metabolic specialization in itaconic acid production: A tale of two fungi. Curr. Opin. Biotechnol. 62, 153–159 (2020). 31689647 10.1016/j.copbio.2019.09.014

[15] R. D. Di Lorenzo, I. Serra, D. Porro, P. Branduardi, State of the art on the microbial production of industrially relevant organic acids. Catalysts 12, 234 (2022).

[16] S. Krull, A. Hevekerl, A. Kuenz, U. Prusse, Process development of itaconic acid production by a natural wild type strain of *Aspergillus* terreus to reach industrially relevant final titers. Appl. Microbiol. Biotechnol. 101, 4063–4072 (2017). 28235991 10.1007/s00253-017-8192-x

[17] T. J. Walsh, V. Petraitis, R. Petraitiene, A. Field-Ridley, D. Sutton, M. Ghannoum, T. Sein, R. Schaufele, J. Peter, J. Bacher, Experimental pulmonary aspergillosis due to *Aspergillus terreus*: Pathogenesis and treatment of an emerging fungal pathogen resistant to amphotericin B. J Infect Dis 188, 305–319 (2003). 12854088 10.1086/377210

[18] Q. Gao, J. Liu, L. Liu, Relationship between morphology and itaconic acid production by *Aspergillus terreus*. J. Microbiol. Biotechnol. 24, 168–176 (2014). 24169454 10.4014/jmb.1303.03093

[19] L. Karaffa, C. P. Kubicek, Citric acid and itaconic acid accumulation: Variations of the same story? Appl. Microbiol. Biotechnol. 103, 2889–2902 (2019). 30758523 10.1007/s00253-018-09607-9 PMC6447509

[20] L. Karaffa, R. Diaz, B. Papp, E. Fekete, E. Sandor, C. P. Kubicek, A deficiency of manganese ions in the presence of high sugar concentrations is the critical parameter for achieving high yields of itaconic acid by *Aspergillus terreus*. Appl. Microbiol. Biotechnol. 99, 7937–7944 (2015). 26078111 10.1007/s00253-015-6735-6

[21] A. Kuenz, Y. Gallenmuller, T. Willke, K. D. Vorlop, Microbial production of itaconic acid: Developing a stable platform for high product concentrations. Appl. Microbiol. Biotechnol. 96, 1209–1216 (2012). 22752264 10.1007/s00253-012-4221-y

[22] B. C. Saha, G. J. Kennedy, M. J. Bowman, N. Qureshi, R. O. Dunn, Factors affecting production of itaconic acid from mixed sugars by *Aspergillus terreus*. Appl. Biochem. Biotechnol. 187, 449–460 (2019). 29974379 10.1007/s12010-018-2831-2

[23] A. Hevekerl, A. Kuenz, K. D. Vorlop, Filamentous fungi in microtiter plates-an easy way to optimize itaconic acid production with *Aspergillus terreus*. Appl. Microbiol. Biotechnol. 98, 6983–6989 (2014). 24737061 10.1007/s00253-014-5743-2

[24] H. Hosseinpour Tehrani, J. Becker, I. Bator, K. Saur, S. Meyer, A. C. Rodrigues Loia, L. M. Blank, N. Wierckx, Integrated strain- and process design enable production of 220 g L^−1^ itaconic acid with *Ustilago maydis*. Biotechnol. Biofuels 12, 263 (2019). 31709012 10.1186/s13068-019-1605-6 PMC6833137

[25] T. Brefort, G. Doehlemann, A. Mendoza-Mendoza, S. Reissmann, A. Djamei, R. Kahmann, *Ustilago maydis* as a pathogen. Annu. Rev. Phytopathol. 47, 423–445 (2009). 19400641 10.1146/annurev-phyto-080508-081923

[26] B. J. Harder, K. Bettenbrock, S. Klamt, Model-based metabolic engineering enables high yield itaconic acid production by *Escherichia coli*. Metab. Eng. 38, 29–37 (2016). 27269589 10.1016/j.ymben.2016.05.008

[27] M. H. Noh, H. G. Lim, S. H. Woo, J. Song, G. Y. Jung, Production of itaconic acid from acetate by engineering acid-tolerant *Escherichia coli* W. Biotechnol. Bioeng. 115, 729–738 (2018). 29197183 10.1002/bit.26508

[28] P. Chang, G. S. Chen, H. Y. Chu, K. W. Lu, C. R. Shen, Engineering efficient production of itaconic acid from diverse substrates in *Escherichia coli*. J. Biotechnol. 249, 73–81 (2017). 28366527 10.1016/j.jbiotec.2017.03.026

[29] J. Blazeck, J. Miller, A. Pan, J. Gengler, C. Holden, M. Jamoussi, H. S. Alper, Metabolic engineering of *Saccharomyces cerevisiae* for itaconic acid production. Appl. Microbiol. Biotechnol. 98, 8155–8164 (2014). 24997118 10.1007/s00253-014-5895-0

[30] A. Otten, M. Brocker, M. Bott, Metabolic engineering of *Corynebacterium glutamicum* for the production of itaconate. Metab. Eng. 30, 156–165 (2015). 26100077 10.1016/j.ymben.2015.06.003

[31] A. H. Hossain, R. van Gerven, K. M. Overkamp, P. S. Lubeck, H. Taspinar, M. Turker, P. J. Punt, Metabolic engineering with ATP-citrate lyase and nitrogen source supplementation improves itaconic acid production in *Aspergillus niger*. Biotechnol. Biofuels 12, 233 (2019). 31583019 10.1186/s13068-019-1577-6 PMC6767652

[32] A. H. Hossain, A. Li, A. Brickwedde, L. Wilms, M. Caspers, K. Overkamp, P. J. Punt, Rewiring a secondary metabolite pathway towards itaconic acid production in *Aspergillus niger*. Microb. Cell Fact. 15, 130 (2016). 27469970 10.1186/s12934-016-0527-2 PMC4965889

[33] J. Blazeck, A. Hill, M. Jamoussi, A. Pan, J. Miller, H. S. Alper, Metabolic engineering of *Yarrowia lipolytica* for itaconic acid production. Metab. Eng. 32, 66–73 (2015). 26384571 10.1016/j.ymben.2015.09.005

[34] C. Zhao, Z. Cui, X. Zhao, J. Zhang, L. Zhang, Y. Tian, Q. Qi, J. Liu, Enhanced itaconic acid production in *Yarrowia lipolytica* via heterologous expression of a mitochondrial transporter MTT. Appl. Microbiol. Biotechnol. 103, 2181–2192 (2019). 30656392 10.1007/s00253-019-09627-z

[35] L. Rong, L. Miao, S. Wang, Y. Wang, S. Liu, Z. Lu, B. Zhao, C. Zhang, D. Xiao, K. Pushpanathan, A. Wong, A. Yu, Engineering *Yarrowia lipolytica* to produce itaconic acid from waste cooking oil. Front. Bioeng. Biotechnol. 10, 888869 (2022). 35547171 10.3389/fbioe.2022.888869 PMC9083544

[36] D. Gopaliya, V. Kumar, S. K. Khare, Recent advances in itaconic acid production from microbial cell factories. Biocatal. Agric. Biotechnol. 36, 102130 (2021).

[37] C. Madzak, *Yarrowia lipolytica* strains and their biotechnological applications: How natural biodiversity and metabolic engineering could contribute to cell factories improvement. J. Fungi 7, 548 (2021). 10.3390/jof7070548 PMC8307478 34356927

[38] M. Groenewald, T. Boekhout, C. Neuveglise, C. Gaillardin, P. W. van Dijck, M. Wyss, *Yarrowia lipolytica*: Safety assessment of an oleaginous yeast with a great industrial potential. Crit. Rev. Microbiol. 40, 187–206 (2014). 23488872 10.3109/1040841X.2013.770386

[39] O. Konzock, J. Norbeck, Deletion of MHY1 abolishes hyphae formation in *Yarrowia lipolytica* without negative effects on stress tolerance. PLOS ONE 15, e0231161 (2020). 32243483 10.1371/journal.pone.0231161 PMC7122783

[40] E. Carsanba, S. Papanikolaou, H. Erten, Production of oils and fats by oleaginous microorganisms with an emphasis given to the potential of the nonconventional yeast *Yarrowia lipolytica*. Crit. Rev. Biotechnol. 38, 1230–1243 (2018). 29764205 10.1080/07388551.2018.1472065

[41] W. Bao, Z. Li, X. Wang, R. Gao, X. Zhou, S. Cheng, Y. Men, L. Zheng, Approaches to improve the lipid synthesis of oleaginous yeast *Yarrowia lipolytica*: A review. Renew. Sustain. Energy Rev. 149, 111386 (2021).

[42] S. V. Kamzolova, I. G. Morgunov, Metabolic peculiarities of the citric acid overproduction from glucose in yeasts *Yarrowia lipolytica*. Bioresour. Technol. 243, 433–440 (2017). 28688326 10.1016/j.biortech.2017.06.146

[43] R. Sestric, G. Munch, N. Cicek, R. Sparling, D. B. J. B. T. Levin, Growth and neutral lipid synthesis by *Yarrowia lipolytica* on various carbon substrates under nutrient-sufficient and nutrient-limited conditions. Bioresour. Technol. 164, 41–46 (2014). 24835917 10.1016/j.biortech.2014.04.016

[44] S. Zaghen, O. Konzock, J. Fu, E. J. J. B. Kerkhoven, Abolishing storage lipids induces protein misfolding and stress responses in *Yarrowia lipolytica*. J. Ind. Microbiol. Biotechnol. 50, kuad031 (2023). 37742215 10.1093/jimb/kuad031 PMC10563384

[45] N. Poorinmohammad, J. Fu, B. Wabeke, E. J. Kerkhoven, Validated growth rate-dependent regulation of lipid metabolism in *Yarrowia lipolytica*. Int. J. Mol. Sci. 23, 8517 (2022). 35955650 10.3390/ijms23158517 PMC9369070

[46] M. Zhao, X. Lu, H. Zong, J. Li, B. Zhuge, Itaconic acid production in microorganisms. Biotechnol. Lett. 40, 455–464 (2018). 29299715 10.1007/s10529-017-2500-5

[47] J. L. Avalos, G. R. Fink, G. Stephanopoulos, Compartmentalization of metabolic pathways in yeast mitochondria improves the production of branched-chain alcohols. Nat. Biotechnol. 31, 335–341 (2013). 23417095 10.1038/nbt.2509 PMC3659820

[48] S. Zhang, F. Guo, Q. Yang, Y. Jiang, S. Yang, J. Ma, F. Xin, T. Hasunuma, A. Kondo, W. Zhang, M. Jiang, Improving methanol assimilation in *Yarrowia lipolytica* via systematic metabolic engineering combined with compartmentalization. Green Chem. 25, 183–195 (2023).

[49] Y. Ma, J. Li, S. Huang, G. Stephanopoulos, Targeting pathway expression to subcellular organelles improves astaxanthin synthesis in *Yarrowia lipolytica*. Metab. Eng. 68, 152–161 (2021). 34634493 10.1016/j.ymben.2021.10.004

[50] K. Yang, Y. Qiao, F. Li, Y. Xu, Y. Yan, C. Madzak, J. Yan, Subcellular engineering of lipase dependent pathways directed towards lipid related organelles for highly effectively compartmentalized biosynthesis of triacylglycerol derived products in *Yarrowia lipolytica*. Metab. Eng. 55, 231–238 (2019). 31382013 10.1016/j.ymben.2019.08.001

[51] C. Holkenbrink, M. I. Dam, K. R. Kildegaard, J. Beder, J. Dahlin, D. Domenech Belda, I. Borodina, EasyCloneYALI: CRISPR/Cas9-based synthetic toolbox for engineering of the yeast yarrowia lipolytica. Biotechnol. J. 13, e1700543 (2018). 29377615 10.1002/biot.201700543

[52] J. R. Elmore, G. N. Dexter, D. Salvachua, J. Martinez-Baird, E. A. Hatmaker, J. D. Huenemann, D. M. Klingeman, G. L. T. Peabody, D. J. Peterson, C. Singer, G. T. Beckham, A. M. Guss, Production of itaconic acid from alkali pretreated lignin by dynamic two stage bioconversion. Nat. Commun. 12, 2261 (2021). 33859194 10.1038/s41467-021-22556-8 PMC8050072

[53] S.-Y. Zeng, H.-H. Liu, T.-Q. Shi, P. Song, L.-J. Ren, H. Huang, X.-J. Ji, Recent advances in metabolic engineering of *Yarrowia lipolytica* for lipid overproduction. Eur. J. Lipid Sci. Technol. 120, 1700352 (2018).

[54] J. Wang, R. Ledesma-Amaro, Y. Wei, B. Ji, X. J. Ji, Metabolic engineering for increased lipid accumulation in *Yarrowia lipolytica*–A review. Bioresour. Technol. 313, 123707 (2020). 32595069 10.1016/j.biortech.2020.123707

[55] E. J. Kerkhoven, Y. M. Kim, S. Wei, C. D. Nicora, T. L. Fillmore, S. O. Purvine, B. J. Webb-Robertson, R. D. Smith, S. E. Baker, T. O. Metz, J. Nielsen, Leucine biosynthesis is involved in regulating high lipid accumulation in *Yarrowia lipolytica*. MBio 8, e00857-17 (2017). 28634240 10.1128/mBio.00857-17 PMC5478895

[56] B. Zetsche, J. S. Gootenberg, O. O. Abudayyeh, I. M. Slaymaker, K. S. Makarova, P. Essletzbichler, S. E. Volz, J. Joung, J. van der Oost, A. Regev, E. V. Koonin, F. Zhang, Cpf1 is a single RNA-guided endonuclease of a class 2 CRISPR-Cas system. Cell 163, 759–771 (2015). 26422227 10.1016/j.cell.2015.09.038 PMC4638220

[57] J. L. Zhang, Y. Z. Peng, D. Liu, H. Liu, Y. X. Cao, B. Z. Li, C. Li, Y. J. Yuan, Gene repression via multiplex gRNA strategy in *Y. lipolytica*. Microb. Cell Fact. 17, 62 (2018). 29678175 10.1186/s12934-018-0909-8 PMC5910576

[58] C. Schwartz, K. Frogue, A. Ramesh, J. Misa, I. Wheeldon, CRISPRi repression of nonhomologous end-joining for enhanced genome engineering via homologous recombination in *Yarrowia lipolytica*. Biotechnol. Bioeng. 114, 2896–2906 (2017). 28832943 10.1002/bit.26404

[59] I. A. Drinnenberg, D. E. Weinberg, K. T. Xie, J. P. Mower, K. H. Wolfe, G. R. Fink, D. P. Bartel, RNAi in budding yeast. Science 326, 544–550 (2009). 19745116 10.1126/science.1176945 PMC3786161

[60] Z. Tao, H. Yuan, M. Liu, Q. Liu, S. Zhang, H. Liu, Y. Jiang, D. Huang, T. Wang, Yeast extract: Characteristics, production, applications and future perspectives. J. Microbiol. Biotechnol. 33, 151–166 (2023). 36474327 10.4014/jmb.2207.07057 PMC9998214

[61] L.-B. Yang, X.-B. Zhan, Z.-Y. Zheng, J.-R. Wu, M.-J. Gao, C.-C. Lin, A novel osmotic pressure control fed-batch fermentation strategy for improvement of erythritol production by *Yarrowia lipolytica* from glycerol. Bioresour. Technol. 151, 120–127 (2014). 24215768 10.1016/j.biortech.2013.10.031

[62] O. Konzock, S. Zaghen, J. Fu, E. J. Kerkhoven, Urea is a drop-in nitrogen source alternative to ammonium sulphate in *Yarrowia lipolytica*. iScience 25, 105703 (2022). 36567708 10.1016/j.isci.2022.105703 PMC9772842

[63] G. Barth, T. Scheuber, Cloning of the isocitrate lyase gene (ICL1) from *Yarrowia lipolytica* and characterization of the deduced protein. Mol. Gen. Genet. 241, 422–430 (1993). 8246896 10.1007/BF00284696

[64] T. Tabuchi, T. Satoh, Distinction between isocitrate lyase and methylisocitrate lyase in Candida lipolytica. Agric. Biol. Chem. 40, 1863–1869 (1976).

[65] F. Chen, P. Lukat, A. A. Iqbal, K. Saile, V. Kaever, J. van den Heuvel, W. Blankenfeldt, K. Büssow, F. Pessler, Crystal structure of cis-aconitate decarboxylase reveals the impact of naturally occurring human mutations on itaconate synthesis. Proc. Natl. Acad. Sci. U.S.A. 116, 20644–20654 (2019). 31548418 10.1073/pnas.1908770116 PMC6789909

[66] W. Sabra, R. R. Bommareddy, G. Maheshwari, S. Papanikolaou, A. P. Zeng, Substrates and oxygen dependent citric acid production by *Yarrowia lipolytica*: Insights through transcriptome and fluxome analyses. Microb. Cell Fact. 16, 78 (2017). 28482902 10.1186/s12934-017-0690-0 PMC5421321

[67] S. V. Kamzolova, N. V. Shishkanova, I. G. Morgunov, T. V. Finogenova, Oxygen requirements for growth and citric acid production of. FEMS Yeast Res. 3, 217–222 (2003). 12702455 10.1016/S1567-1356(02)00188-5

[68] E. Riscaldati, M. Moresi, M. Petruccioli, F. Federici, Effect of pH and stirring rate on itaconate production by *Aspergillus terreus*. J. Biotechnol. 83, 219–230 (2000). 11051419 10.1016/s0168-1656(00)00322-9

[69] C. Verduyn, E. Postma, W. A. Scheffers, J. P. Van Dijken, Effect of benzoic acid on metabolic fluxes in yeasts: A continuous-culture study on the regulation of respiration and alcoholic fermentation. Yeast 8, 501–517 (1992). 1523884 10.1002/yea.320080703

[70] K. J. Livak, T. D. Schmittgen, Analysis of relative gene expression data using real-time quantitative PCR and the 2^−ΔΔCT^ method. Methods 25, 402–408 (2001). 11846609 10.1006/meth.2001.1262

[71] E. J. Kerkhoven, K. R. Pomraning, S. E. Baker, J. Nielsen, Regulation of amino-acid metabolism controls flux to lipid accumulation in *Yarrowia lipolytica*. NPJ Syst. Biol. Appl. 2, 16005 (2016). 28725468 10.1038/npjsba.2016.5 PMC5516929

[72] N. E. Lewis, K. K. Hixson, T. M. Conrad, J. A. Lerman, P. Charusanti, A. D. Polpitiya, J. N. Adkins, G. Schramm, S. O. Purvine, D. Lopez-Ferrer, K. K. Weitz, R. Eils, R. König, R. D. Smith, B. Ø. Palsson, Omic data from evolved *E. coli* are consistent with computed optimal growth from genome-scale models. Mol. Syst. Biol. 6, 390 (2010). 20664636 10.1038/msb.2010.47 PMC2925526

