# Supplementary material for: Reprogramming Yarrowia lipolytica metabolism for efficient synthesis of itaconic acid from flask to semipilot scale
Source: Sci Adv. 2024 Aug 9;10(32):eadn0414. doi: 10.1126/sciadv.adn0414 (PMC11313960; doi:10.1126/sciadv.adn0414)
Supplement: Supplementary file 1 — Figs. S1 to S13 Tables S1 to S8 [file sciadv.adn0414_sm.pdf]

Science Advances
AAAS

Supplementary Materials for

Reprogramming *Yarrowia lipolytica* metabolism for efficient synthesis of itaconic acid from flask to semipilot scale

Jing Fu *et al.*

Corresponding author: Eduard J. Kerkhoven, eduardk@chalmers.se

Sci. Adv. **10**, eadn0414 (2024)
DOI: 10.1126/sciadv.adn0414

This PDF file includes:

Figs. S1 to S13
Tables S1 to S8

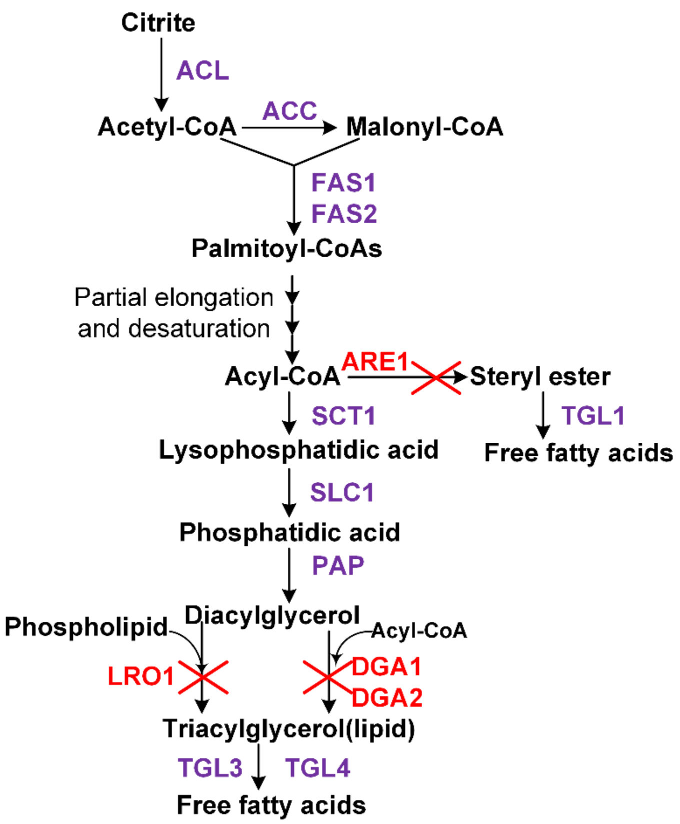

Fig. S1. Lipid synthesis pathways in *Y. lipolytica*. ACC1, acetyl-CoA carboxylase; ACL, ATP-citrate lyase; ARE1, Acyl-CoA:sterol O-acyltransferase; DGA1, Acyl-CoA diacylglycerol acyltransferase 1; DGA2, Acyl-CoA diacylglycerol acyltransferase 2; FAS1 and FAS2, fatty acid synthase complex; LRO1, phospholipid: diacylglycerol acyltransferase; RAP, phosphatidic acid phosphatase; SCT1, glycerol-3-phosphate acyltransferase; SLC1, lysophosphatidic acid acyltransferase; TGL1, Cholesterol esterase; TGL3 Triacylglycerol lipase 3; TGL4, Triacylglycerol lipase 4.

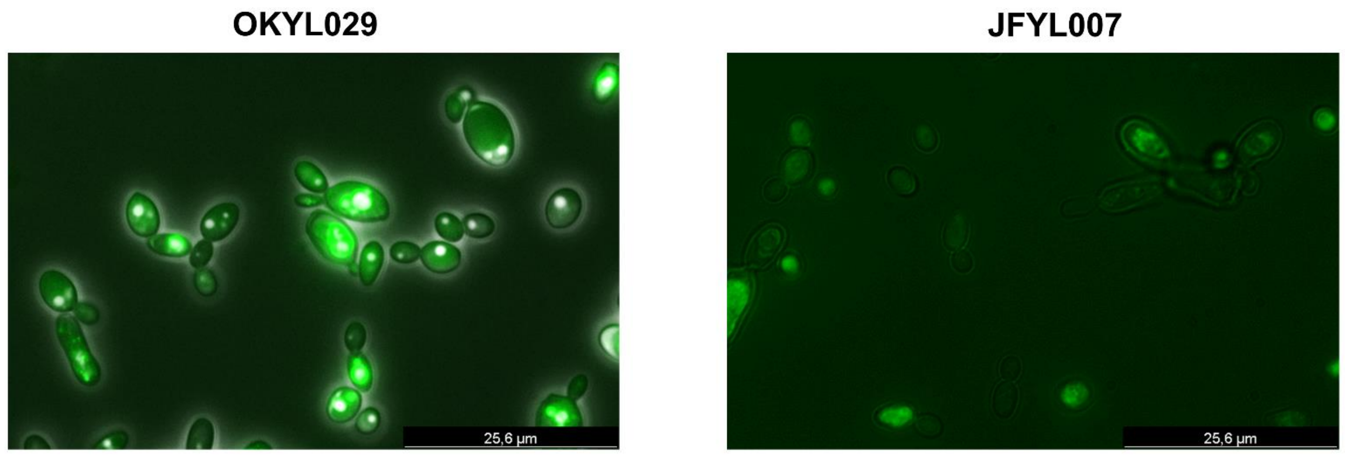

Fig. S2 Micrographs of *Y. lipolytica* cells stained with fluorescent dye Bodipy. Cells were harvested in Delft medium with NL condition.

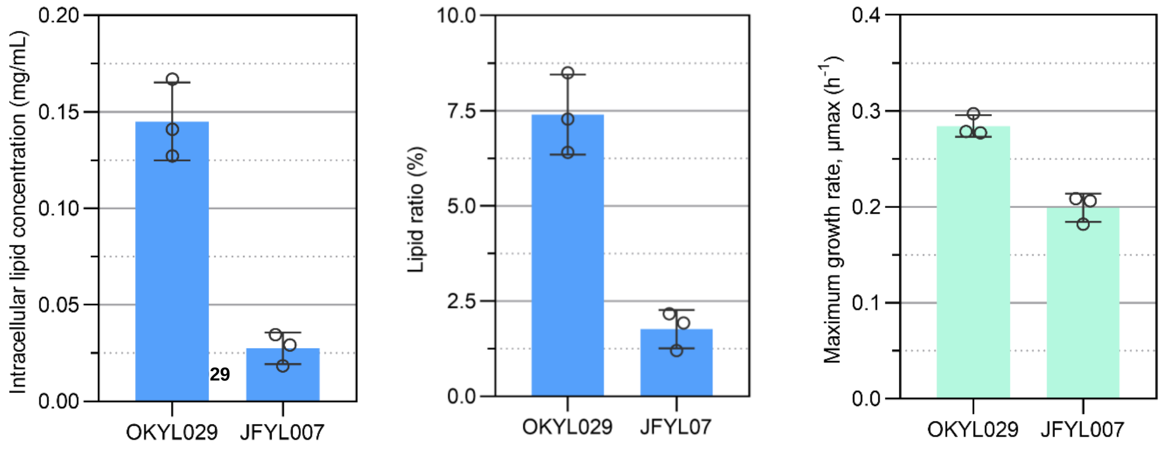

Fig. S3 Intracellular total lipid concentration, lipid ratio as percentage of biomass, and maximum specific growth rates of the starting strain OKYL029 and lipid blocked strain JFYL007. Cells were harvested in Delft medium with NL condition (C/N=116). All data represent the mean of n = 3 biologically independent samples and error bars show standard deviation.

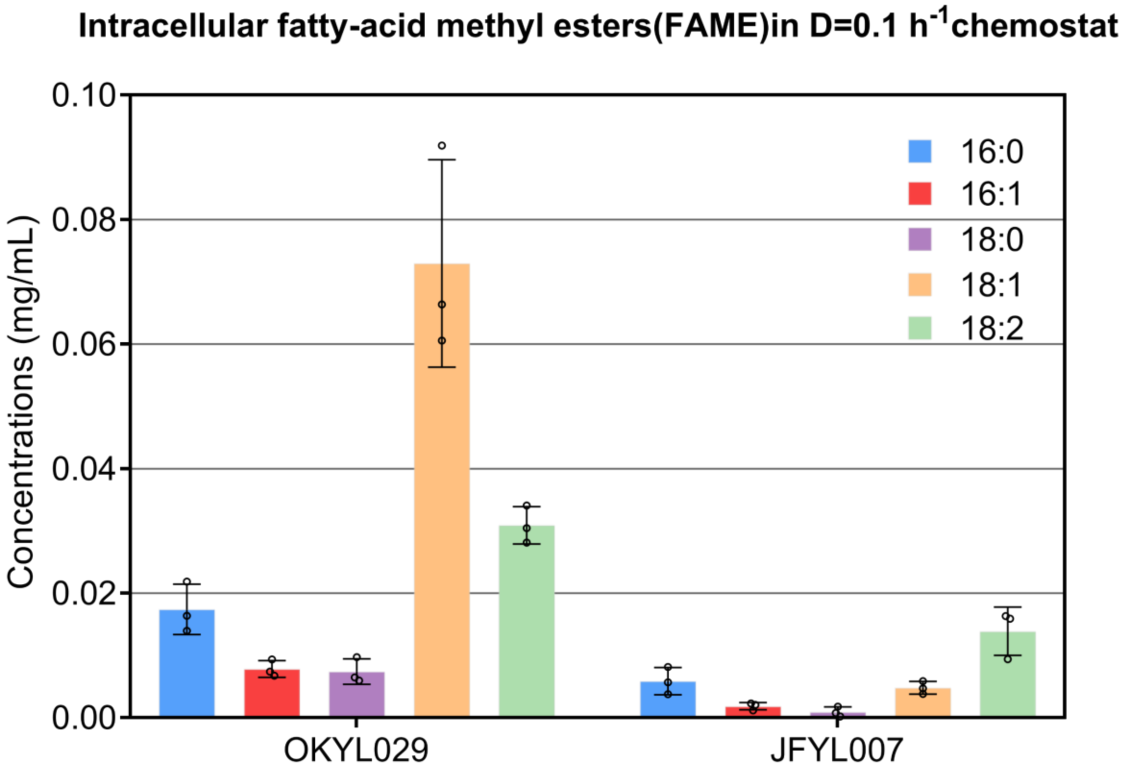

Fig. S4 Fatty acid methyl esters test for OKYL 029 and JFYL007. Cells were harvested in Delft medium with NL condition (C/N=116). All data represent the mean of n = 3 biologically independent samples and error bars show standard deviation.

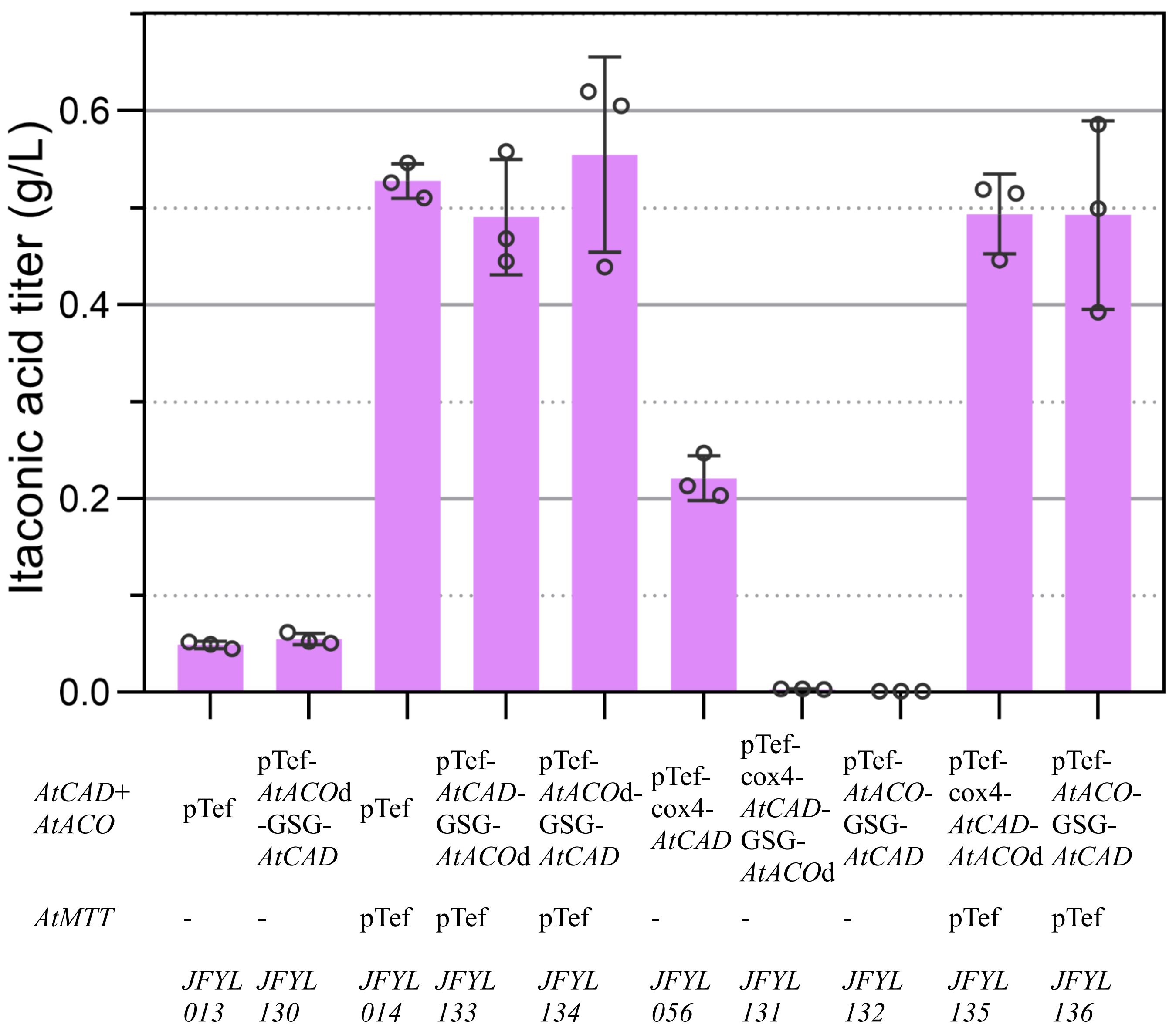

Fig. S5 IA production with *AtACO* and *AtCAD* fusion proteins with flexible linker GSG. Strains was cultivated in NR condition for 4 days. Data represent the mean of n = 3 biologically independent samples and error bars show standard deviation.

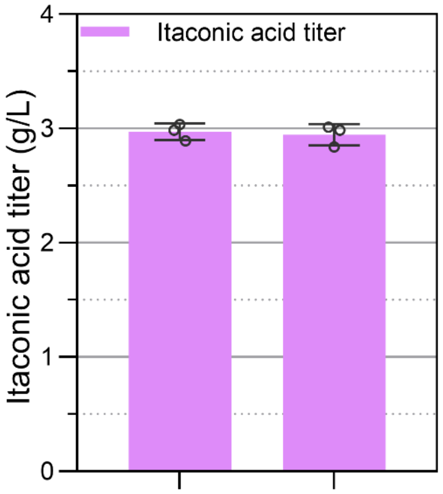

AtCAD	pTef	pTef
AtMTT	p*GPD*	p*GPD*
Copy number	6	6
*AtACO*d	-	pTef
	JFYL128	JFYL129

Fig. S6 IA production in JFYL128 and JFYL129. Strains was cultivated in NR condition for 4 days. Data represent the mean of $n = 3$ biologically independent samples and error bars show standard deviation.

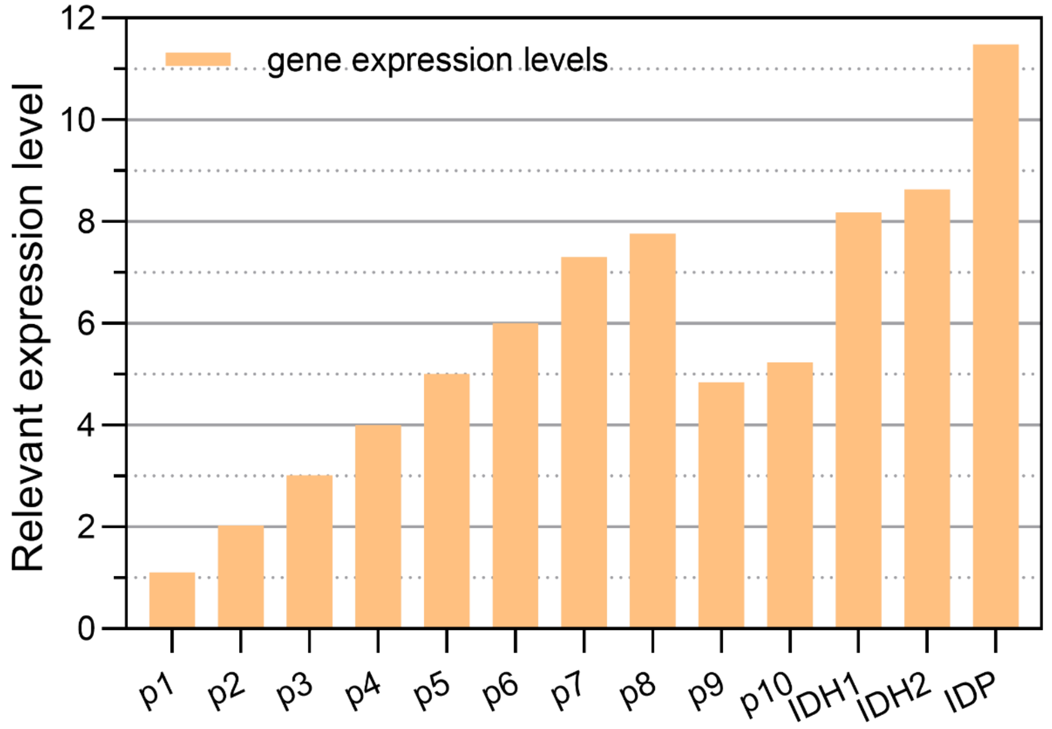

Gene	Gene	Mean WTN	Note
p1	YALI0A00550g	1.1	Stable expression
p2	YALI0D10967g	2.03	
p3	YALI0B16192g	3.01	
p4	YALI0F20680g	4.01	
p5	YALI0E13596g	5.01	
p6	YALI0B07667g	6	
p7	YALI0D05731g	7.30	Decreased expression at high C/N ratio
p8	YALI0C16885g	7.76	
p9	YALI0F03344g	4.84	
p10	YALI0A14388g	5.23	
IDH1	YALI0D06303g	8.18	
IDH2	YALI0E05137g	8.63	
IDP	YALI0F04095g	11.48	

Fig. S7 Gene expression level of candidates for weaker promoter changing. 1000 bp upstream sequences of the corresponding gene for promoters p1 to p10 were selected as the promoter sequences.

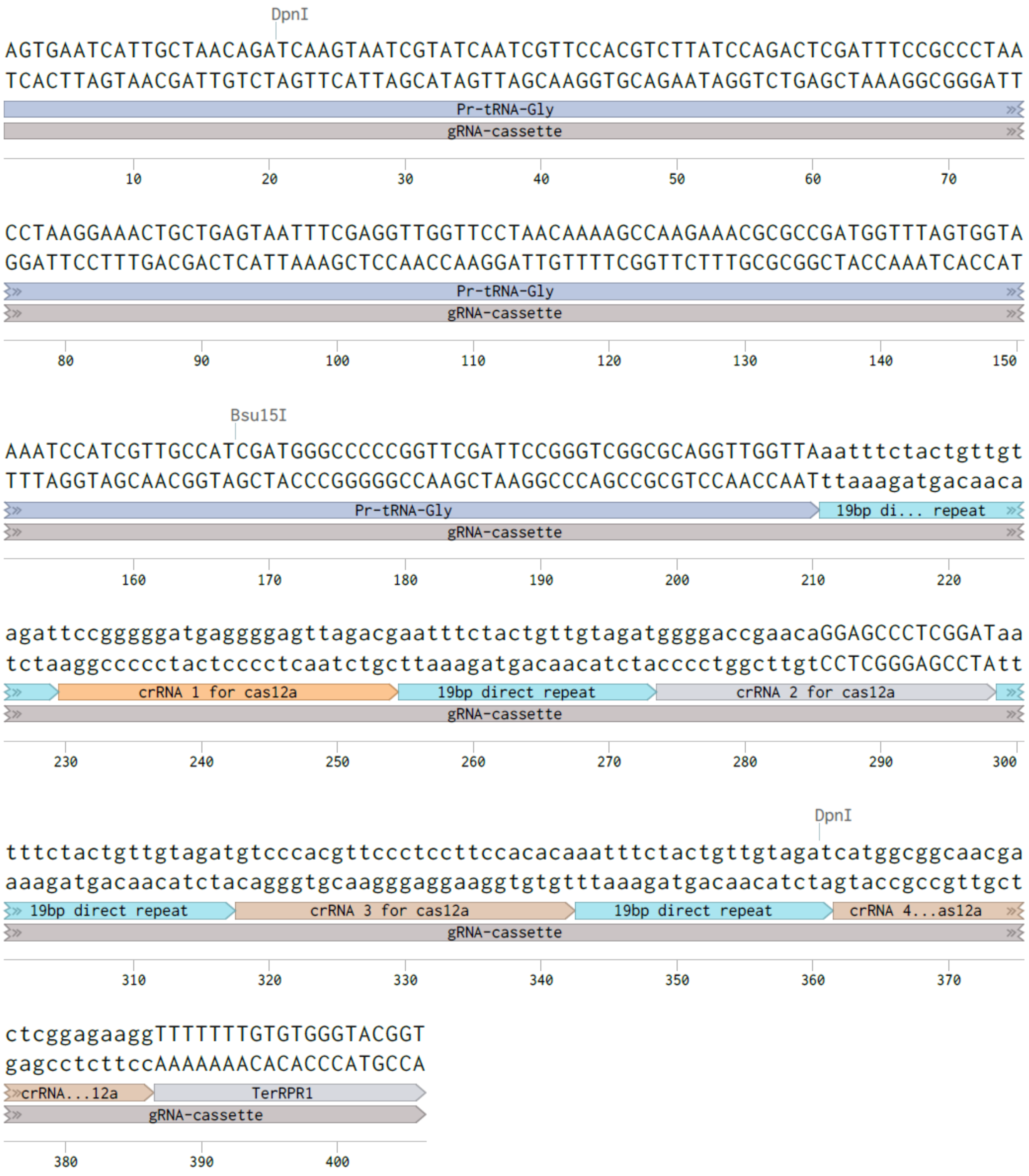

Fig. S8 Four site-specific crRNA sequences targeting to *IDH1* promote and gene.

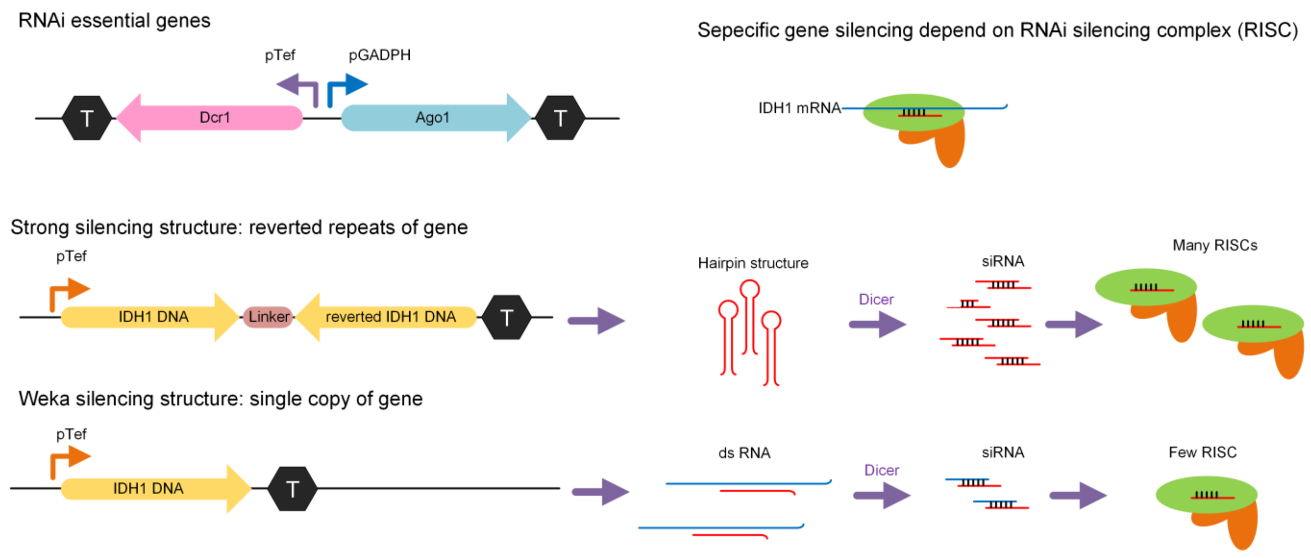

Fig. S9 RNAi was employed and established in this study based on previous report (59).

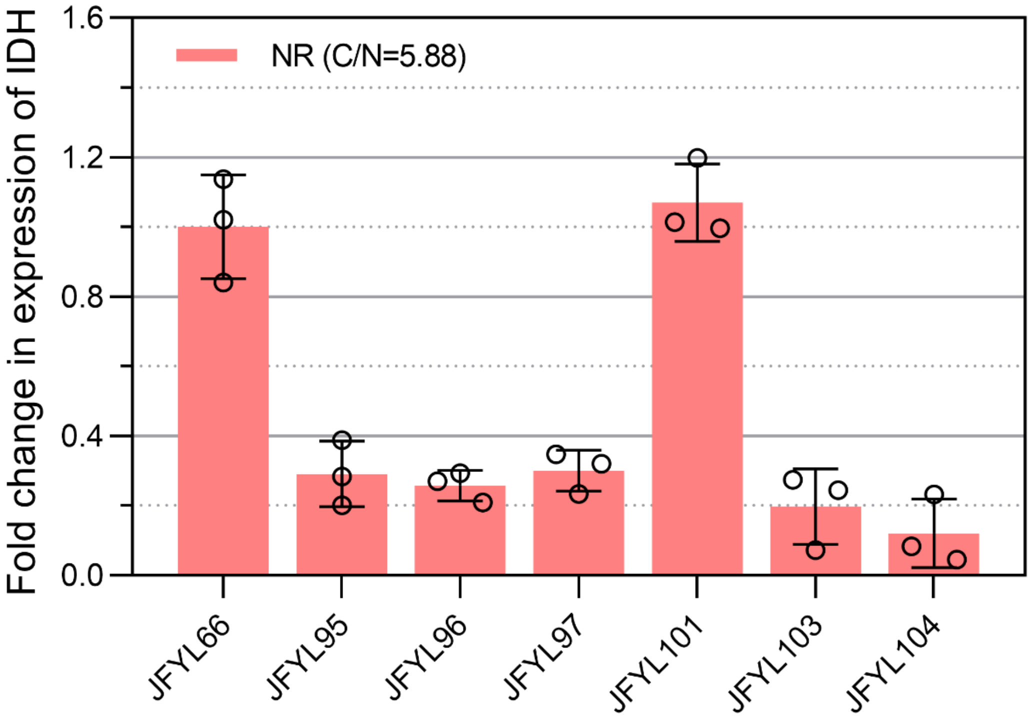

Fig. S10 The gene expression level of IDH. Strains was cultivated in flasks in NR condition and harvested when the OD reached around 1.5. Data represent the mean of n = 3 biologically independent samples and error bars show standard deviation.

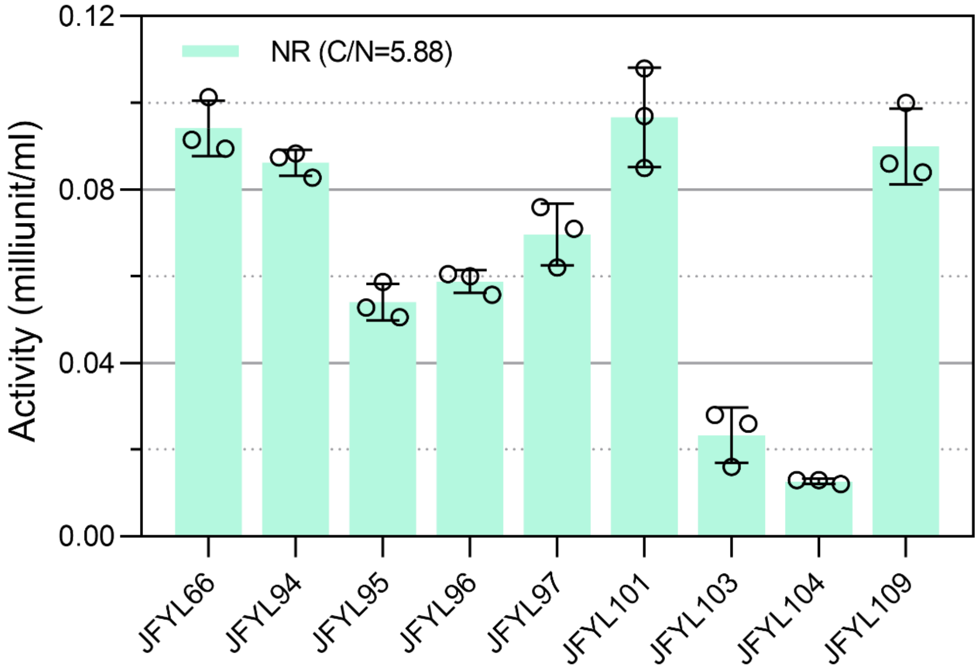

Fig. S11 The IDH assay. Strains was cultivated in flasks in NR condition and harvested after 2 days. Data represent the mean of n = 3 biologically independent samples and error bars show standard deviation.

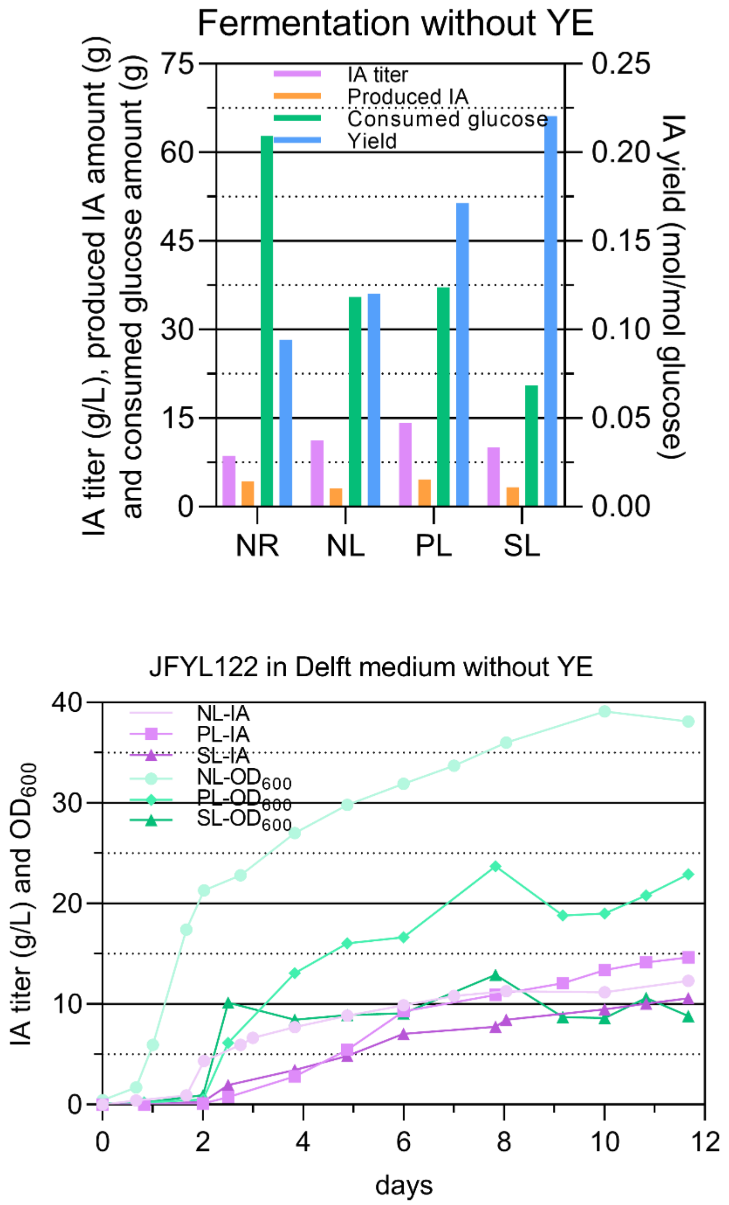

Fig. S12 JFYL122 was cultivated in 1L bioreactors without yeast extract. Top, itaconic acid production under nitrogen replete (C/N=22, $C_{100}N_{10}$), NL (C/N=88, $C_{100}N_{2.5}$), PL and SL conditions with pH at 5.5; Bottom, time course of itaconic acid titer and OD_{600}. Cultivations were carried out in two replicates, and the presented cultivations depict a single representative cultivation.

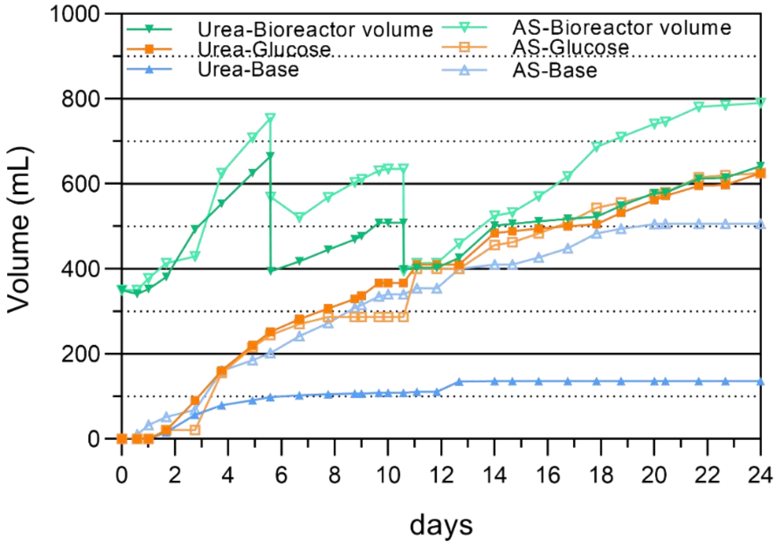

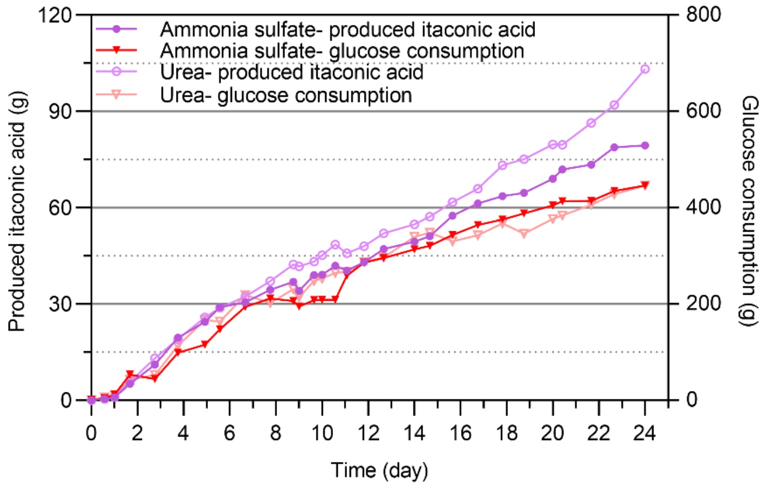

Fig. S13 produced IA and consumed glucose amount of JFYL122 when cultivated in 1L bioreactors with AS or urea. Top, volume of bioreactor, fed glucose and base; Bottom, Amount of produced itaconic acid and consumed glucose. Cultivations were carried out in two replicates, and the presented cultivations depict a single representative cultivation.

Table S1 IA production in different microorganisms.

Strain	Strategy for strain engineering	Carbon source	Fermentation type and tips	Titer (g/L)	Yield (mol/mol)	Productivity (g/L/h)	Produced IA amount (g)	Ref
***A. terreus* DSM 23081**	Wild type	Glucose	Fed-batch in 1.5 L bioreactor, pH at 3.4 in production phase	160	0.78	0.56	NA	(*16*)
***A. terreus* DSM 23081**	Wild type	Glucose	Fed-batch in 15 L bioreactor,	150	0.64	0.64		(*16*)
***U. maydis* MB215**	Overexpression of native rai1 and *mttA* from *A. tereus*; deletion of cyp3 and fuz7 coding itaconate oxidase	Glucose	Fed-batch in 5 L bioreactor; in situ product crystallization with $CaCO_3$.	220	0.33	0.46	NA	(*24*)
***A. niger* AB 1.13**	Overexpression of *acl*2, *citB*, *cadA*, *mttA* and *mfsA*	Glucose	Fed-batch in 10L bioreactor	42.7	0.26	0.18	NA	(*31*)
***Saccharomyces cerevisiae* XYY286**	Overexpression of *cadA* from *A. tereus* and *acc2*; Deletion of *hxk2*	Glucose	Flask	0.535	-	-	NA	(29)
***E. coli* ita36A**	Overexpression of *cadA* from *A. tereus*; native *icd* under temperature control; deletion of *aceA*, *pta*, *pykF pykA*	Glucose	Fed-batch in 1L bioreactor; temperature controlled two-stage process	47	0.62	0.39	NA	(26)
Corneybacterium glutamicum	Heterologous expression of *cadA*, from *A. terreus*; mutated icd variant for reduced isocitrate dehydrogenase activity	Acetate	Fed-batch in 42L bioreactor; pH and DO-coupled feeding strategy.	29.2		0.63	NA	(30)
Yarrowia lipolytica	Cytosolic coexpression of CAD and ACOnoMLS	Glucose	Fed-batch	4.6	0.058	0.027	NA	(*33*)
Yarrowia lipolytica	Heterologous expression of *cadA*, *acoA*, *mttA* and *mfsA* from *A. terreus*	Glucose	Fed-batch	22	0.056	0.05	NA	(*34*)
Yarrowia lipolytica	Heterologous expression of CAD-ePTS1 and POT1, and deletion of ICL	Cooking oil	Batch in 5L bioreactor	54.55	NA	0.568	NA	(*35*)
***Yarrowia lipolytica* JFYL122**		Glucose	Fed-batch in 1 L bioreactor	130.2	0.320	0.226	103.3	This study
***Yarrowia lipolytica* JFYL122**		Glucose	Fed-batch in 50 L bioreactor	94.1	0.360	0.246	6161.88	This study

Table S2 Technical comparison of *A. Terreus* with the highest titer and JFYL122.

**Comparison**	***Aspergillus terreus***	***Yarrowia lipolytica***
Strain Construction	Wild type	Engineered Strain, derived from W29
Strain safety status	Fungal pathogen	GRAS
Morphological control	Pellet diameter should be approximately 80 μm	Not required, yeast form due to the deletion of *mhy1*
Carbon source	180 g/L Glucose initially	110 g/L Glucose initially
Nitrogen source	3g/L NH_4NO_3 initially, no nitrogen limitation	4.5 g/L Urea initially, no nitrogen limitation
Phosphate source	phosphate limitation, 0.1 g/L KH_2PO_4	19.1 g/L KH_2PO_4, 10.4 g/L K_2HPO4, initially, no phosphate limitation
Sulfur source	1 g/L $MgSO_4$ initially, no sulfur limitation	0.5 g/L $MgSO_4$ initially, no sulfur limitation
Control of manganese concentration	Itaconic acid production is negatively influenced by manganese concentrations higher than 3 μg/L for *Aspergillus terreus*. Glucose was purified by an ion exchanger.	Not required
Main by-product	α-ketoglutaric acid and malic acid	Citric acid
pH control	The pH value was manually adjusted upwards at 3.0 by adding ammonia solution.	The pH value was automatically adjusted at 5.5 by adding 6M KOH.
Bioreactor volume	1.5L and 15L	1L and 50L
Titer	160 g/L (1.5L bioreactor)<br>150 g/L (15L bioreactor)	130.1 g/L (1L bioreactor)<br>94.1 g/L (50L bioreactor)
Yield	0.78 mol/mol (1.5L bioreactor)<br>0.64 mol/mol (15L bioreactor)	0.320 mol/mol (1L bioreactor)<br>0.360 mol/mol (50L bioreactor)
Productivity	0.56 g/L/h (1.5L bioreactor)<br>0.64 g/L/h (15L bioreactor)	0.226 g/L/h (1.5L bioreactor)<br>0.246 g/L/h (15L bioreactor)
Reference	16	This study

Table S3 Strains used in this study.

**Strains ID**	**Genotype**	**Reference**
WT	W29	(*48*)
ST6512	W29Δku70::cas9	(*48*)
OKYL029	W29Δku70::cas9Δmhy1	(*39*)
JFYL007	W29Δku70::cas9Δmhy1Δ*ARE1*Δ*LRO1*Δ*DGA1*Δ*DGA2*	This work
JFYL008	JFYL07, lntE3:p*BR30*-*AtCAD*-tlip2	This work
JFYL009	JFYL07, lntE3:p*ICL1*-*AtCAD*-tlip2	This work
JFYL010	JFYL07, lntE3:p4UASTef-*AtCAD*-tlip2	This work
JFYL013	JFYL07, lntE3:pTef-*AtCAD*-tlip2	This work
JFYL014	JFYL07, lntE1:pTef-*AtMTT*-tlip2, lntE3:pTef-*AtCAD*-tlip2	This work
JFYL016	JFYL07Δ*ICL1*	This work
JFYL017	JFYL07Δ*ICL2*	This work
JFYL018	JFYL07Δ*ICL1*Δ*ICL2*	This work
JFYL021	JFYL07Δ*IDP*	This work
JFYL023	OKYL29, lntE3:pTef-*AtCAD*-tlip2	This work
JFYL025	OKYL29, lntE1:pTef-*AtMTT*-tlip2, lntE3:pTef-*AtCAD*-tlip2	This work
JFYL028	JFYL07Δ*ICL1*Δ*ICL2*Δ*IDP*	This work
JFYL029	JFYL07Δ*ICL1*, lntE3:pTef-*AtCAD*-tlip2	This work
JFYL030	JFYL07Δ*ICL2*, lntE3:pTef-*AtCAD*-tlip2	This work
JFYL031	JFYL07Δ*ICL1*Δ*ICL2*, lntE3:pTef-*AtCAD*-tlip2	This work
JFYL032	JFYL07Δ*IDP*, lntE3:pTef-*AtCAD*-tlip2	This work
JFYL033	JFYL07Δ*ICL1*Δ*ICL2*Δ*IDP*, lntE3:pTef-*AtCAD*-tlip2	This work
JFYL035	JFYL07, lntE3:pTefin-*AtCAD*-tlip2	This work
JFYL036	JFYL07, lntE1:pTefin-*AtMTT*-tlip2, lntE3:pTef-*AtCAD*-tlip2	This work
JFYL039	JFYL07, lntE1:pTef-*AtMTT*-tlip2, lntC3:pTef-*AtCAD*-tlip2-p*GPD*-*AtMTT*-tpex20	This work
JFYL040	JFYL07, lntE3:pTef-*AtCAD*-tlip2, lntC3:pTef-*AtCAD*-tlip2-p*GPD*-*AtMTT*-tpex20	This work
JFYL041	JFYL07, lntE1:pTef-*AtMTT*-tlip2, lntE3:pTef-*AtCAD*-tlip2, lntC3:pTef-*AtCAD*-tlip2-p*GPD*-*AtMTT*-tpex20	This work
JFYL051	JFYL07Δ*ICL1*Δ*ICL2*, lntC3:pTef-*AtCAD*-tlip2-p*GPD*-*AtMTT*-tpex20	This work
JFYL053	JFYL07Δ*ICL1*Δ*ICL2*Δ*IDP*, lntC3:pTef-*AtCAD*-tlip2-p*GPD*-*AtMTT*-tpex20	This work
JFYL054	JFYL07, lntE1:pTef-*AtMTT*-tlip2, lntE3:pTef-*AtCAD*-tlip2,lntC3:pTef-*AtACO*d-tlip2	This work
JFYL055	JFYL07, lntE1:pTef-*AtMTT*-tlip2, lntE3:pTef-*AtCAD*-tlip2, lntC3:pTef-*AtACO*-tlip2	This work
JFYL056	JFYL07, lntD1:pTef-Ylcox4-*AtCAD*-tlip2	This work
JFYL061	JFYL07, lntE1:pTef-Akctp-tlip2, lntE3:pTef-*AtCAD*-tlip2	This work
JFYL062	JFYL07, lntE1:pTefin-Akctp-tlip2, lntE3:pTef-*AtCAD*-tlip2	This work
JFYL063	JFYL07, lntE1:pTef-Ummtt1-tlip2, lntE3:pTef-*AtCAD*-tlip2	This work
JFYL064	JFYL07, lntE1:pTefin-Ummtt1-tlip2, lntE3:pTef-*AtCAD*-tlip2	This work
JFYL065	JFYL07, lntC2:pTef-*AtCAD*-tlip2-p*GPD*-*AtMTT*-tpex20	This work
JFYL066	JFYL07, lntC3:pTef-*AtCAD*-tlip2-p*GPD*-*AtMTT*-tpex20	This work
JFYL067	JFYL07, lntD1:pTef-*AtCAD*-tlip2-p*GPD*-*AtMTT*-tpex20	This work
JFYL068	JFYL07, lntE1:pTef-*AtCAD*-tlip2-p*GPD*-*AtMTT*-tpex20	This work
JFYL069	JFYL07, lntE2:pTef-*AtCAD*-tlip2-p*GPD*-*AtMTT*-tpex20	This work
JFYL070	JFYL07, lntE3:pTef-*AtCAD*-tlip2-p*GPD*-*AtMTT*-tpex20	This work
JFYL071	JFYL07, lntC2:pTef-*AtCAD*-tlip2-p*GPD*-*AtMTT*-tpex20, lntC3:pTef-*AtCAD*-tlip2-p*GPD*-*AtMTT*-tpex20	This work
JFYL074	JFYL07, lntC2:pTef-*AtCAD*-tlip2-p*GPD*-*AtMTT*-tpex20, lntC3:pTef-*AtCAD*-tlip2-p*GPD*-*AtMTT*-tpex20, lntD1:pTef-*AtCAD*-tlip2-p*GPD*-*AtMTT*-tpex20	This work
JFYL075	JFYL07, lntC2:pTef-*AtCAD*-tlip2-p*GPD*-*AtMTT*-tpex20, lntC3:pTef-*AtCAD*-tlip2-p*GPD*-*AtMTT*-tpex20, lntD1:pTef-*AtCAD*-tlip2-p*GPD*-*AtMTT*-tpex20, lntE2:pTef-*AtCAD*-tlip2-p*GPD*-*AtMTT*-tpex20	This work

JFYL076	JFYL07, lntC2:pTef-*AtCAD*-tlip2-p*GPD-AtMTT*-tpex20, lntC3:pTef-*AtCAD*-tlip2-p*GPD-AtMTT*-tpex20, lntD1:pTef-*AtCAD*-tlip2-p*GPD-AtMTT*-tpex20, lntE1:pTef-*AtCAD*-tlip2-p*GPD-AtMTT*-tpex20, lntE1:pTef-*AtCAD*-tlip2-p*GPD-AtMTT*-tpex20	This work
JFYL077	JFYL07, lntC2:pTef-*AtCAD*-tlip2-p*GPD-AtMTT*-tpex20, lntC3:pTef-*AtCAD*-tlip2-p*GPD-AtMTT*-tpex20, lntD1:pTef-*AtCAD*-tlip2-p*GPD-AtMTT*-tpex20, lntE1:pTef-*AtCAD*-tlip2-p*GPD-AtMTT*-tpex20, lntE2:pTef-*AtCAD*-tlip2-p*GPD-AtMTT*-tpex20, lntE3:pTef-*AtCAD*-tlip2-p*GPD-AtMTT*-tpex20	This work
JFYL083	JFYL07, lntD1:pTef-Ylcox4-*AtCAD*-tlip2, lntE1:pTef-SceDIC-tlip2	This work
JFYL084	JFYL07, lntD1:pTef-Ylcox4-*AtCAD*-tlip2, lntE1:pTefin-SceDIC-tlip2	This work
JFYL085	JFYL07, lntD1:pTef-Ylcox4-*AtCAD*-tlip2, lntE1:pTef-MumSlc-tlip2	This work
JFYL086	JFYL07, lntD1:pTef-Ylcox4-*AtCAD*-tlip2, lntE1:pTefin-MumSlc-tlip2	This work
JFYL087	JFYL07, lntD1:pTef-Ylcox4-*AtCAD*-tlip2, lntE1:pTef-AthDtc-tlip2	This work
JFYL088	JFYL07, lntD1:pTef-Ylcox4-*AtCAD*-tlip2, lntE1:pTefin-AthDtc-tlip2	This work
JFYL089	JFYL07, lntD1:pTef-Ylcox4-*AtCAD*-tlip2, lntE3:pTef-*AtCAD*-tlip2,	This work
JFYL090	JFYL07, lntE3:pTef-UmTad1-tlip2	This work
JFYL091	JFYL07, lntE1:pTef-*AtMTT*-tlip2, lntE3:pTef-UmTad1-tlip2	This work
JFYL092	JFYL07, lntE1:pTef-UmTad1-tlip2, lntE3:pTef-UmTad1-tlip2	This work
JFYL093	JFYL07, lntD1:pTef-*AtMTT*-tlip2, lntE1:pTef-UmTad1-tlip2, lntE3:pTef-UmTad1-tlip2	This work
JFYL094	JFYL07, lntC3:pTef-*AtCAD*-tlip2-p*GPD-AtMTT*-tpex20, lntD1:pTef-YlAmpd-tlip2,	This work
JFYL095	JFYL07, lntC3:pTef-*AtCAD*-tlip2-p*GPD-AtMTT*-tpex20, p*IDH1*::p9	This work
JFYL096	JFYL07, lntC3:pTef-*AtCAD*-tlip2-p*GPD-AtMTT*-tpex20, p*IDH1*::p10	This work
JFYL097	JFYL07, lntD1:CRISPRi-*IDH1*	This work
JFYL100	JFYL07, lntC3:lip2t-*AtCAD*-Tefp-*GPD*p-*AtMTT*-pex20t, lntE1-pex2t-Dcr1-Tefp-*GPD*p-Ago1-lip2t	This work
JFYL101	JFYL07, lntC3:lip2t-*AtCAD*-Tefp-*GPD*p-*AtMTT*-pex20t, lntE1-pex2t-Dcr1-Tefp-*GPD*p-Ago1-lip2t, lntE3::Tefp-Ri1-pex20t	This work
JFYL102	JFYL07, lntC3:lip2t-*AtCAD*-Tefp-*GPD*p-*AtMTT*-pex20t, lntE1-pex2t-Dcr1-Tefp-*GPD*p-Ago1-lip2t, lntE3::Tefp-Ri2-pex20t	This work
JFYL103	JFYL07, lntC3:lip2t-*AtCAD*-Tefp-*GPD*p-*AtMTT*-pex20t, lntE1-pex2t-Dcr1-Tefp-*GPD*p-Ago1-lip2t, lntE3::Tefp-Ri3-pex20t	This work
JFYL104	JFYL07, lntC3:lip2t-*AtCAD*-Tefp-*GPD*p-*AtMTT*-pex20t, lntE1-pex2t-Dcr1-Tefp-*GPD*p-Ago1-lip2t, lntE3::Tefp-Ri4-pex20t	This work
JFYL105	JFYL07, lntC3:lip2t-*AtCAD*-Tefp-*GPD*p-*AtMTT*-pex20t, lntE1-pex2t-Dcr1-Tefp-*GPD*p-Ago1-lip2t, lntE3::Tefp-Ri5-pex20t	This work
JFYL106	JFYL07, lntC3:lip2t-*AtCAD*-Tefp-*GPD*p-*AtMTT*-pex20t, lntE1-pex2t-Dcr1-Tefp-*GPD*p-Ago1-lip2t, lntE3::Tefp-Ri6-pex20t	This work
JFYL107	JFYL07, lntC3:lip2t-*AtCAD*-Tefp-*GPD*p-*AtMTT*-pex20t, lntE1-pex2t-Dcr1-Tefp-*GPD*p-Ago1-lip2t, lntE3::Tefp-Ri7-pex20t	This work
JFYL108	JFYL07, lntC3:lip2t-*AtCAD*-Tefp-*GPD*p-*AtMTT*-pex20t, lntE1-pex2t-Dcr1-Tefp-*GPD*p-Ago1-lip2t, lntE3::Tefp-Ri8-pex20t	This work
JFYL109	JFYL07, lntC3:lip2t-*AtCAD*-Tefp-*GPD*p-*AtMTT*-pex20t, lntE1-pex2t-Dcr1-Tefp-*GPD*p-Ago1-lip2t, lntE3::Tefp-Ri9-pex20t	This work
JFYL110	JFYL07, lntC3:lip2t-*AtCAD*-Tefp-*GPD*p-*AtMTT*-pex20t, lntE1-pex2t-Dcr1-Tefp-*GPD*p-Ago1-lip2t, lntE3::Tefp-Ri10-pex20t	This work
JFYL114	JFYL07, lntE1:p4UASTef-*AtCAD*, lntE3:p4UASTef-*AtMTT*	This work
JFYL115	JFYL07, lntC3:pTef-*AtCAD*-tlip2-p*GPD-AtMTT*-tpex20, lntE1:p4UASTef-*AtCAD*	This work
JFYL116	JFYL07, lntC3:pTef-*AtCAD*-tlip2-p*GPD-AtMTT*-tpex20, lntE3:p4UASTef-*AtMTT*	This work
JFYL117	JFYL07, lntC2:pTef-*AtCAD*-tlip2-p*GPD-AtMTT*-tpex20, lntC3:pTef-*AtCAD*-tlip2-p*GPD-AtMTT*-tpex20, lntD1:pTef-*AtCAD*-tlip2-p*GPD-AtMTT*-tpex20, lntE2:pTef-*AtCAD*-tlip2-p*GPD-AtMTT*-tpex20, lntE1-pex2t-Dcr1-Tefp-*GPD*p-Ago1-lip2t, lntE3::Tefp-Ri3-pex20t	This work
JFYL118	JFYL07, lntC2:pTef-*AtCAD*-tlip2-p*GPD-AtMTT*-tpex20, lntC3:pTef-*AtCAD*-tlip2-p*GPD-AtMTT*-tpex20, lntD1:pTef-*AtCAD*-tlip2-p*GPD-AtMTT*-tpex20, lntE2:pTef-*AtCAD*-tlip2-p*GPD-AtMTT*-tpex20, lntE1-pex2t-Dcr1-Tefp-*GPD*p-Ago1-lip2t, lntE3::Tefp-Ri4-pex20t	This work

JFYL119	JFYL07, lntC2:pTef-*AtCAD*-tlip2-p*GPD*-*AtMTT*-tpex20, lntC3:pTef-*AtCAD*-tlip2-p*GPD*-*AtMTT*-tpex20, lntD1:pTef-*AtCAD*-tlip2-p*GPD*-*AtMTT*-tpex20, lntE2:pTef-*AtCAD*-tlip2-p*GPD*-*AtMTT*-tpex20, p*IDH1*::p9	This work
JFYL120	JFYL07, lntC2:pTef-*AtCAD*-tlip2-p*GPD*-*AtMTT*-tpex20, lntC3:pTef-*AtCAD*-tlip2-p*GPD*-*AtMTT*-tpex20, lntD1:pTef-*AtCAD*-tlip2-p*GPD*-*AtMTT*-tpex20, lntE2:pTef-*AtCAD*-tlip2-p*GPD*-*AtMTT*-tpex20, p*IDH1*::p10	This work
JFYL121	JFYL07, lntC2:pTef-*AtCAD*-tlip2-p*GPD*-*AtMTT*-tpex20, lntC3:pTef-*AtCAD*-tlip2-p*GPD*-*AtMTT*-tpex20, lntD1:pTef-*AtCAD*-tlip2-p*GPD*-*AtMTT*-tpex20, lntE2:pTef-*AtCAD*-tlip2-p*GPD*-*AtMTT*-tpex20, lntE1-p4UASTef-*AtMTT*-pex2t	This work
JFYL122	JFYL07, lntC2:pTef-*AtCAD*-tlip2-p*GPD*-*AtMTT*-tpex20, lntC3:pTef-*AtCAD*-tlip2-p*GPD*-*AtMTT*-tpex20, lntD1:pTef-*AtCAD*-tlip2-p*GPD*-*AtMTT*-tpex20, lntE1:pTef-*AtCAD*-tlip2-p*GPD*-*AtMTT*-tpex20, lntE2:pTef-*AtCAD*-tlip2-p*GPD*-*AtMTT*-tpex20, lntE3:pTef-*AtCAD*-tlip2-p*GPD*-*AtMTT*-tpex20, p*IDH1*::p9	This work
JFYL123	JFYL07, lntC2:pTef-*AtCAD*-tlip2-p*GPD*-*AtMTT*-tpex20, lntC3:pTef-*AtCAD*-tlip2-p*GPD*-*AtMTT*-tpex20, lntD1:pTef-*AtCAD*-tlip2-p*GPD*-*AtMTT*-tpex20, lntE1:pTef-*AtCAD*-tlip2-p*GPD*-*AtMTT*-tpex20, lntE2:pTef-*AtCAD*-tlip2-p*GPD*-*AtMTT*-tpex20, lntE3:pTef-*AtCAD*-tlip2-p*GPD*-*AtMTT*-tpex20, p*IDH1*::p10	This work
JFYL124	JFYL07, lntE1-pex2t-Dcr1-Tefp-*GPD*p-Ago1-lip2t, lntE3::Tefp-Ri3-pex20t	This work
JFYL125	JFYL07, lntE1-pex2t-Dcr1-Tefp-*GPD*p-Ago1-lip2t, lntE3::Tefp-Ri4-pex20t	This work
JFYL128	JFYL07, lntE1:3*pTef-*AtCAD*-GSG-*AtACO*d-tlip2, lntD1:3*pTef-*AtCAD*-GSG-*AtACO*d-tlip2	This work
JFYL129	JFYL07, lntE1:3*pTef-*AtCAD*-GSG-*AtACO*d-tlip2, lntD1:3*pTef-*AtCAD*-GSG-*AtACO*d-tlip2, b lntC3:pTef-*AtACO*d-tlip2	This work
JFYL130	JFYL07, lntD1:pTef-*AtACO*d-GSG-*AtCAD*-tlip2	This work
JFYL131	JFYL07, lntD1:pTef-cox4-*AtCAD*-GSG-*AtACO*d-tlip2	This work
JFYL132	JFYL07, lntD1:pTef-*AtACO*-GSG-*AtCAD*-tlip2	This work
JFYL133	JFYL07, lntE1:pTef-*AtMTT*, lntD1:pTef-*AtCAD*-GSG-*AtACO*d-tlip2	This work
JFYL134	JFYL07, lntE1:pTef-*AtMTT*, lntD1:pTef-*AtACO*d-GSG-*AtCAD*-tlip2	This work
JFYL135	JFYL07, lntE1:pTef-*AtMTT*, lntD1:pTef-cox4-*AtCAD*-GSG-*AtACO*d-tlip2	This work
JFYL136	JFYL07, lntE1:pTef-*AtMTT*, lntD1:pTef-*AtACO*-GSG-*AtCAD*-tlip2	This work

Table S4 Plasmids used in this study.

**Plasmid ID**	**Full name**	**Purpose/comments**	**Reference**
OKEC003	pgRNA-*ARE1*	*ARE1* deletion	(*66*)
JFEC001	pgRNA-*LRO1*	*LRO1* deletion	This study
JFEC002	pgRNA-*DGA1*	*DGA1* deletion	This study
JFEC003	pgRNA-*DGA2*	*DGA2* deletion	This study
JFEC004	pgRNA-*ICL1*	*ICL1* deletion	This study
JFEC005	pgRNA-*ICL2*	*ICL2* deletion	This study
JFEC006	pgRNA-*IDH1*	*IDH1* deletion	This study
JFEC007	pgRNA-*IDH2*	*IDH2* deletion	This study
JFEC008	pgRNA-*IDP*	*IDP* deletion	This study
AKEC08	pgRNA-*IDH1*-promoter	*IDH1* promoter changing	This study
JFEC011	pE3-p*BR30*-*AtCAD*	Overexpression of *AtCAD* under *BR30* promoter	This study
JFEC012	pE3-p*ICL2*-*AtCAD*	Overexpression of *AtCAD* under *ICL2* promoter	This study
JFEC013	pE3-pTef-*AtCAD*	Overexpression of *AtCAD* under Tef promoter	This study
JFEC014	pE3-p4UASTef-*AtCAD*	Overexpression of *AtCAD* under 4UASTef promoter	This study
JFEC015	pE3-pTefin-*AtCAD*	Overexpression of *AtCAD* under *BR30* promoter	This study
JFEC017	pD1-pTef-cox4-*AtCAD*	Overexpression of *AtCAD* with cox4 mitochondrial leading sequence under Tef promoter	This study
JFEC021	pE1-pTef-*AtMTT*	Overexpression of *AtMTT* under Tef promoter	This study
JFEC022	pE1-p4UASTef-*AtMTT*	Overexpression of *AtMTT* under 4UASTef promoter	This study
JFEC023	pE1-pTefin-*AtMTT*	Overexpression of *AtMTT* under Tef intron promoter	This study
JFEC024	pC2-pTef-*AtCAD*-tlip2-p*GPD*-*AtMTT*-tpex20	Overexpression of *AtCAD* under Tef promoter and *AtMTT* under *GPD* promoter at lntC2 locus	This study
JFEC025	pC3-pTef-*AtCAD*-tlip2-p*GPD*-*AtMTT*-tpex20	Overexpression of *AtCAD* under Tef promoter and *AtMTT* under *GPD* promoter at lntC3 locus	This study
JFEC026	pD1-pTef-*AtCAD*-tlip2-p*GPD*-*AtMTT*-tpex20	Overexpression of *AtCAD* under Tef promoter and *AtMTT* under *GPD* promoter at lntD1 locus	This study
JFEC027	pE1-pTef-*AtCAD*-tlip2-p*GPD*-*AtMTT*-tpex20	Overexpression of *AtCAD* under Tef promoter and *AtMTT* under *GPD* promoter at lntE1 locus	This study
JFEC028	pE2-pTef-*AtCAD*-tlip2-p*GPD*-*AtMTT*-tpex20	Overexpression of *AtCAD* under Tef promoter and *AtMTT* under *GPD* promoter at lntE2 locus	This study
JFEC029	pE3-pTef-*AtCAD*-tlip2-p*GPD*-*AtMTT*-tpex20	Overexpression of *AtCAD* under Tef promoter and *AtMTT* under *GPD* promoter at lntE3 locus	This study
JFEC031	pC3-pTef-MuSlc	Overexpression of *MuSlc* under Tef promoter	This study
JFEC032	pC3-pTefin-MuSlc	Overexpression of *MuSlc* under Tef intron promoter	This study
JFEC033	pC3-pTef-AthDlc	Overexpression of *AthDlc* under Tef promoter	This study
JFEC034	pC3-pTefin-AthDlc	Overexpression of *AthDlc* under Tef intron promoter	This study
JFEC035	pC3-pTef-SceDlc	Overexpression of *SceDlc* under Tef promoter	This study
JFEC036	pC3-pTefin-SceDlc	Overexpression of *SceDlc* under Tef intron promoter	This study
JFEC037	pE1-pTef-Ummtt1	Overexpression of *Ummtt1* under Tef promoter	This study
JFEC038	pE1-pTefin-Ummtt1	Overexpression of *Ummtt1* under Tef intron promoter	This study
JFEC039	pE1-pTef-AkCtp	Overexpression of *MuSlc* under Tef promoter	This study
JFEC040	pE1-pTefin-AkCtp	Overexpression of *MuSlc* under Tef intron promoter	This study
JFEC041	pC3-pTef-*AtACO*d	Overexpression of *AtACO*d under Tef promoter	This study
JFEC042	pC3-pTef-*AtACO*	Overexpression of *AtACO* under Tef promoter	This study
JFEC043	pD1-pTef-*AtACO*d-GSG-*AtCAD*	Overexpression of *AtACO*d-*AtCAD* fused protein with a GSG linker under Tef promoter	This study
JFEC044	pD1-pTef-*AtCAD*-GSG-*AtACO*d	Overexpression of *AtCAD*-*AtACO*d fused protein with a GSG linker under Tef promoter	This study
JFEC045	pD1-pTef-cox4-GSG-*AtACO*d	Overexpression of *AtCAD*-*AtACO*d fused protein with a GSG linker and with cox4 mitochondrial leading sequence under Tef promoter	This study

JFEC046	pD1-pTef-*AtACO*-GSG-*AtCAD*	Overexpression of *AtACO*-*AtCAD* fused protein with a GSG linker under Tef promoter	This study
JFEC100	plntE1-Pex20t-Dcr1-Tefp-*GPD*p-Ago1-lip2t	For RNAi establishment, overexpression of Dcr1 under Tef promoter and Ago1 under *GPD* promoter	This study
JFEC101	plntE3-Tefp-*IDH1*-inverted repeats1	Overexpression of *IDH1* inverted repeats 1, which is 170bp	This study
JFEC102	plntE3-Tefp-*IDH1*-inverted repeats2	Overexpression of *IDH1* inverted repeats 2, which is 500bp	This study
JFEC103	plntE3-Tefp-*IDH1*-inverted repeats3	Overexpression of *IDH1* inverted repeats 3, which is 1k	This study
JFEC104	plntE3-Tefp-*IDH1*-inverted repeats4	Overexpression of *IDH1* inverted repeats 4, which is 1742bp (full length)	This study
JFEC105	plntE3-Tefp-*IDH1*-inverted repeats5	Overexpression of *IDH1* inverted repeats 5, which is 170bp	This study
JFEC106	plntE3-Tefp-*IDH1*-inverted repeats6	Overexpression of *IDH1* inverted repeats 6, which is 170bp	This study
JFEC107	plntE3-Tefp-*IDH1*-inverted repeats7	Overexpression of *IDH1* inverted repeats 7, which is	This study
JFEC108	plntE3-Tefp-*IDH1*-single1	Overexpression of *IDH1* single repeats 1, which is 170bp from N terminal	This study
JFEC109	plntE3-Tefp-*IDH1*-single2	Overexpression of *IDH1* single repeats 2, which is 1742bp full length of cDNA	This study
JFEC110	plntE3-Tefp-*IDH1*-single3	Overexpression of *IDH1* single repeats 3, which is 170bp from C terminal	This study
JFEC113	plntE1-Tefp-Adi1-lip2t	Overexpression of Adi1 under Tef promoter	This study
JFEC114	plntE3-Tefp-Tad1-lip2t	Overexpression of Tad1 under Tef promoter	This study
JFEC115	plntE1-4UASTefp-*AtMTT*-lip2t	Overexpression of *AtMTT* under 4UASTefp promoter	This study
JFEC116	plntE3-4UASTefp-*AtCAD*-lip2t	Overexpression of *AtCAD* under 4UASTefp promoter	This study
JFEC117	pD1-pTef-AMPD	Overexpression of native AMPD under Tef promoter	This study
JFEC127	pD1-3*(Pex20t-*AtCAD*-Tefp-*GPD*p-*AtMTT*-lip2t)	Overexpression of 3 cassettes at lntD1 locus, and each cassette consists *AtCAD* under Tef promoter and *AtMTT* under *GPD* promoter	This study
JFEC158	pCRISPRi ddcas12a	For CRISPRi establishment, overexpression of ddcas12a and 4 gRNAs for *IDH1* b	This study
JFEC159	pE1-Pex20t-*AtCAD*-Tefp-*GPD*p-*AtMTT*-lip2t*3	Overexpression of 3 cassettes at lntE1 locus, and each cassette consists *AtCAD* under Tef promoter and *AtMTT* under *GPD* promoter	This study

Table S5 Primers used in this study.

**Primer ID**	**Oligos**	**Comments**
F001=*LRO1*-Del-cr-F	ACTCAACGCCAAGTACCCGGgttttagagct	gRNA expression cassette for *DGA1* deletion for *LRO1* deletion with 20 bp site-specific gRNA BioBrick
F002=*LRO1*-Del-cr-R	CCGGGTACTTGGCGTTGAGTtaaccaacct	For *LRO1* gRNA plasmids with 20 bp site-specific gRNA BioBrick
F007=*LRO1*-Del-ck-F	CTGAATTTCCCGATTTATTC	check primer for *LRO1* deletion
F008=*LRO1*-Del-ck-R	GAAACGCGCATATGATAGTG	check primer for *LRO1* deletion
F009=*DGA1*-Del-ck-F	CGAGCGAATCGCACACAAAC	check primer for *DGA1* deletion
F010=*DGA1*-Del-ck-R	CCATGTATGACATTCGAGCC	check primer for *DGA1* deletion
F011=*DGA2*-Del-ck-F	CAAACGAGTATACTTGTAGC	check primer for *DGA2* deletion
F012=*DGA2*-Del-ck-R	CACAGTCACGAAAACCATAC	check primer for *DGA2* deletion
F013=gRNA-cass1-F	cgtgcgaUagtgaatcattgctaacagatc	PR-10607 in EasyClone
F014=gRNA-cass1-R	cacgcgaUaccgtacccacacaaaaaaagcaccaccgactc	PR-10604 in EasyClone
F017=gRNA-cass2-F	AGTGCAGGUagtgaatcattgctaacagatc	PR-15790 in EasyClone
F018=gRNA-cass2-R	ACCTGCACUaccgtacccacacaaaaaaagcac	PR-15791 in EasyClone
F027=*LRO1*-RM-F	TAAAAAAAGTGTAATCGGCTTTTTCCGGTTGATCACAACCATCAAGAGTCCGTTTTGTAGAGTAATATGTTTTGTATATCACACTGATG	Repair fragments for *LRO1* deletion
F028=*LRO1*-RM-R	CATCAGTGTGATATACAAAACATATTACTCTACAAAACGGACTCTTGATGGTTGTGATCAACCGGAAAAAGCCGATTACACTTTTTTA	Repair fragments for *LRO1* deletion
F029=*DGA1*-RM-F	CAAAAAAATACTCATTAGCTATTTGCCTAACCCAGGCAGTTTTCCAGCTTTTGTTTTGTGTGACTTGTCTGTTGCCTGTTGTTAGAAGAA	Repair fragments for *DGA1* deletion
F030=*DGA1*-RM-R	TTCTTCTAACAACAGGCAACAGACAAGTCACACAAAACAAAAGCTGGAAAACTGCCTGGGTTAGGCAAATAGCTAATGAGTATTTTTTTG	Repair fragments for *DGA1* deletion
F031=*DGA2*-RM-F	TGTCAATATTATTTATCACTGTAAAGGCTACTGATGAGTGTTATGTTTGCGGGCGGTACGGGTACAGCGACTTTGGGTGTAGCTATGGTG	Repair fragments for *DGA2* deletion
F032=*DGA2*-RM-R	CACCATAGCTACACCCAAAGTCGCTGTACCCGTACCGCCCGCAAACATAACACTCATCAGTAGCCTTTACAGTGATAAATAATATTGACA	Repair fragments for *DGA2* deletion
F033=gRNA-cass-ck-F	TTGGAGGCGACGTGGCAG	Check primer for gRNA plasmids
F034=gRNA-cass-ck-R	AAATGCGGCCGCGAATGC	Check primer for gRNA plasmids
F035=*DGA1*-Del-cr2-F	GGCGTCTTCAACTACGATGTgttttagagct	gRNA expression cassette for *DGA1* deletion
F036=*DGA1*-Del-cr2-R	ACATCGTAGTTGAAGACGCCtaaccaacct	gRNA expression cassette for *DGA1* deletion
F037=*DGA1*-Del-cr3-F	AGCATAGCATGAAAATTGTGgttttagagct	gRNA expression cassette for *DGA1* deletion
F038=*DGA1*-Del-cr3-R	CACAATTTTCATGCTATGCTtaaccaacct	gRNA expression cassette for *DGA1* deletion
F039=*DGA2*-Del-cr2-F	GAGCCAGACCATCATAGACAgttttagagct	gRNA expression cassette for *DGA2* deletion
F040=*DGA2*-Del-cr2-R	TGTCTATGATGGTCTGGCTCtaaccaacct	gRNA expression cassette for *DGA2* deletion
F041=*DGA2*-Del-cr3-F	GAGCCAGACCATCATAGACAgttttagagct	gRNA expression cassette for *DGA2* deletion
F042=*DGA2*-Del-cr3-R	TGTCTATGATGGTCTGGCTCtaaccaacct	gRNA expression cassette for *DGA2* deletion
F049=*IDH1*-RM-F	TTTTTTTCTTGTCGCCAACTTCGTGGAACCCCCAAAGAAATCACAACGATATAACGATAATGATAATGATTTGATGTAATGTTGGAAGCG	Repair fragments
F050=*IDH1*-RM-R	CGCTTCCAACATTACATCAAATCATTATCATTATCGTTATATCGTTGTGATTTCTTTGGGGGTTCCACGAAGTTGGCGACAAGAAAAAAA	Repair fragments
F051=*IDH2*-RM-F	TCAATAATTCGTTCAATGTCATAATATCATCTAAATCATACATAATGTGGATGTGGATATGTTTTCGATTAGACGACAAGATGTTGCCAA	Repair fragments

F052=*IDH2*-RM-R	TTGGCAACATCTTGTCGTCTAATCGAAAACATATCCACATCCACATTATGTATGATTTAGATGATATTATGACATTGAACGAATTATTGA	Repair fragments
F053=*IDP*-RM-F	ATCGTGCGGTTGTGTCGCATAAATCATTAATATAAACATTTTCCCGGCTGGCAAGGGAGGGTCTGTGGGGGTTCAACGTGGGGTTGCATG	Repair fragments
F054=*IDP*-RM-R	CATGCAACCCCACGTTGAACCCCCACAGACCCTCCCTTGCCAGCCGGGAAAATGTTTATATTAATGATTTATGCGACACAACCGCACGAT	Repair fragments
F055=*ICL1*-RM-F	GCCTCCTTCACCTACGGTGCCCTCGACCCCGTCCAGGTGACCCAGGCAGTTTGTTTAGCAAAATATATTTAACGAGTTTGATAGAGGCGC	Repair fragments
F056=*ICL1*-RM-R	GCGCCTCTATCAAACTCGTTAAATATATTTTGCTAAACAAACTGCCTGGGTCACCTGGACGGGGTCGAGGGCACCGTAGGTGAAGGAGGC	Repair fragments
F057=*ICL2*-RM-F	AACACGAGCGGTTGCTGATTCGGTCACTAAAAAACAAGTGCAAAAGCATGGTACGATGTGTAACTAGCTAACTGGATGAAAGAACGGCAA	Repair fragments
F058=*ICL2*-RM-R	TTGCCGTTCTTTCATCCAGTTAGCTAGTTACACATCGTACCATGCTTTTGCACTTGTTTTTTAGTGACCGAATCAGCAACCGCTCGTGTT	Repair fragments
F059=*IDH1*-Del-cr1-F	GGGTCGCACAGCTCGAAGGGgttttagagct	gRNA expression cassette for deletion
F060=*IDH1*-Del-cr1-R	CCCTTCGAGCTGTGCGACCCtaaccaacct	gRNA expression cassette for deletion
F061=*IDH1*-Del-cr2-F	GTGTCGCAGCATCATCACGGgttttagagct	gRNA expression cassette for deletion
F062=*IDH1*-Del-cr2-R	CCGTGATGATGCTGCGACACtaaccaacct	gRNA expression cassette for deletion
F063=*IDH2*-Del-cr1-F	ACTCACCTTAGCCAGGTAGGgttttagagct	gRNA expression cassette for deletion
F064=*IDH2*-Del-cr1-R	CCTACCTGGCTAAGGTGAGTtaaccaacct	gRNA expression cassette for deletion
F065=*IDH2*-Del-cr2-F	GAGGTGTCGCAGAAGCATGGgttttagagct	gRNA expression cassette for deletion
F066=*IDH2*-Del-cr2-R	CCATGCTTCTGCGACACCTCtaaccaacct	gRNA expression cassette for deletion
F067=*IDP*-Del-cr1-F	TGGTGGACATGGTTTTAGAAgttttagagct	gRNA expression cassette for deletion
F068=*IDP*-Del-cr1-R	TTCTAAAACCATGTCCACCAtaaccaacct	gRNA expression cassette for deletion
F069=*IDP*-Del-cr2-F	ATCCTCGTATGTGCTGACCGgttttagagct	gRNA expression cassette for deletion
F070=*IDP*-Del-cr2-R	CGGTCAGCACATACGAGGATtaaccaacct	gRNA expression cassette for deletion
F071=*ICL1*-Del-cr1-F	CGAGGCATGAAGGCTTACGGgttttagagct	gRNA expression cassette for deletion
F072=*ICL1*-Del-cr1-R	CCGTAAGCCTTCATGCCTCGtaaccaacct	gRNA expression cassette for deletion
F073=*ICL1*-Del-cr2-F	GCTCCCGGTACCAAGAAGTGgttttagagct	gRNA expression cassette for deletion
F074=*ICL1*-Del-cr2-R	CACTTCTTGGTACCGGGAGCtaaccaacct	gRNA expression cassette for deletion
F075=*ICL2*-Del-cr1-F	CGAGATGATGACCTACGACGgttttagagct	gRNA expression cassette for deletion
F076=*ICL2*-Del-cr1-R	CGTCGTAGGTCATCATCTCGtaaccaacct	gRNA expression cassette for deletion
F077=*ICL2*-Del-cr2-F	GTGGATGTCTCTGTCCCACGgttttagagct	gRNA expression cassette for deletion
F078=*ICL2*-Del-cr2-R	CGTGGGACAGAGACATCCACtaaccaacct	gRNA expression cassette for deletion
F079=*IDH1*-Del-ck-F	AGGGGAGTTAGACGGACGTGGG	gRNA expression cassette for deletion
F080=*IDH1*-Del-ck-R	CTTCCGCACATCCTTTCCACCC	gRNA expression cassette for deletion
F081=*IDH2*-Del-ck-F	TACCCAACTCCATCACACCCAG	gRNA expression cassette for deletion
F082=*IDH2*-Del-ck-R	GGTCGGGTACCCGGACGGATGT	gRNA expression cassette for deletion
F083=*IDP*-Del-ck-F	GAGGTGGACTTCCCTGTGCGAG	gRNA expression cassette for deletion
F084=*IDP*-Del-ck-R	GCATAATCTGTGCTGCTTCCCC	gRNA expression cassette for deletion
F085=*ICL1*-Del-ck-F	GGATGAGGTGTTGTGTGGTGGG	gRNA expression cassette for deletion
F086=*ICL1*-Del-ck-R	CACCTCACCTCCCTCACCCCTT	gRNA expression cassette for deletion
F087=*ICL2*-Del-ck-F	GGAGCAAACTGCAGGACGGATG	gRNA expression cassette for deletion
F088=*ICL2*-Del-ck-R	CGTACGATCACGATGTGTGGGT	gRNA expression cassette for deletion
F097=ACOdL1CAD-C3tef-PF	TTCAACGGAATGCGTGCGATAGAGACCGGGTTGGCGGCGC	*AtACO* and *AtCAD* fused protein
F098=ACOdL1CAD-C3tef-G1R	TGCTTGGTCATACCAGAACCGTTGGAGGCGGCCTTTCGAG	*AtACO* and *AtCAD* fused protein
F099=ACOdL1CAD-C3tef-G2F	CTCGAAAGGCCGCCTCCAACGGTTCTGGTATGACCAAGCAGTCTGCCGAC	*AtACO* and *AtCAD* fused protein

F100=CADL1CAOd-D1tef-PF	TTCAACGGAATGCGTGCGATCGCTTGAGGATCCAGAGACCGGGTTGGCGGCGC	*AtACO* and *AtCAD* fused protein
F101=CADL1ACOd-D1tef-G1R	GCCACGGTAGCACCAGAACCCACCAGGGGAGACTTCACGG	*AtACO* and *AtCAD* fused protein
F102=CADL1ACOd-D1tef-G2F	CCGTGAAGTCTCCCCTGGTGGGTTCTGGTGCTACCGTGGCCGACTCGCC	*AtACO* and *AtCAD* fused protein
F106=*AtCAD*-USER-PF	CGTGCGAUAGAGACCGGGTTGGCGGCGC	Promoter amplification
F107=*AtCAD*-USER-PR	ATGACAGAUTTTGAATGATTCTTATACTC	Promoter amplification
F108=*AtCAD*-USER-GF	ATCTGTCAUGCCACAATGACCAAGCAGTCTGCCGACTC	Gene amplification
F109=*AtCAD*-USER-GR	CACGCGAUTTACACCAGGGGAGACTTCACG	Gene amplification
F115=Tefin-PR	CTGCGGTTAGTACTGCAAAAAGTGCTGGTC	Tef intron promoter amplification
F120=PR-14617-ckF	tatccctgtgttgaatc	Check primer for gene integration
F121=PR-14619-ckR	tatcgacccagttagc	Check primer for gene integration
F128=*AtCAD*-g2-tef-cox4-PR	TTGAAGGCGAGCATTGTGGCTTTGAATGATTCTTATACTC	Cox4-*AtCAD* promoter amplification
F129=*AtCAD*-g2-tef-cox4-GF	GCCACAATGCTCGCCTTCAAGTCTCTCCGACCCTCTGCTGTCTCCCGACTGGCAACCTCCACCCGAGCTGCCCACGTCATCTCCACCAAGCAGTCTGCCGACTC	Cox4-*AtCAD* gene amplification
F148=INT-C2-ck-F	AGACGCGAAGGACGACATCC	Check primer for intergration
F149=INT-C2-ck-R	TCGGCATCTTCATTCAGCTG	Check primer for intergration
F150=INT-C3-ck-F	CTCTCCACATTTCCAGATAG	Check primer for intergration
F151=INT-C3-ck-R	CTATCTAATTTCGTGGCCGC	Check primer for intergration
F152=INT-D1-ck-F	GTCGGAAGATGAACGCAATC	Check primer for intergration
F153=INT-D1-ck-R	ATGGCACATGTGCTAGAATG	Check primer for intergration
F154=INT-E1-ck-F	TACACAGGCTCTTCACTCAC	Check primer for intergration
F155=INT-E1-ck-R	CTGGAGATTCTCTCCTAGCT	Check primer for intergration
F156=INT-E2-ck-F	CAACTCCGTCTGGTGTCTCC	Check primer for intergration
F157=INT-E2-ck-R	CGGTCACCATCGCGGTTCTC	Check primer for intergration
F158=INT-E3-ck-F	ATGGGCCACATTGTGACACC	Check primer for intergration
F159=INT-E3-ck-R	CAATCTGGGGAACTTCGCGT	Check primer for intergration
F162=Del-*IDH1*-RM-UF	ttgcggcacaatccggatat	1000 bp repair fragment of deletion
F163=Del-*IDH1*-RM-UR	CATTATCATTATCGTTATATCGTtgtgatttctttgggggttc	1000 bp repair fragment of deletion
F164=Del-*IDH1*-RM-LF	GAACCCCCAAAGAAATCACAacgatataacgataatgataatg	1000 bp repair fragment of deletion
F165=Del-*IDH1*-RM-LR	ttagcgtcaactggaaggcg	1000 bp repair fragment of deletion
F166=Del-*IDH1*-RM-FsnF	ggcactgtgttctatttctg	1000 bp repair fragment of deletion
F167=Del-*IDH1*-RM-FsnR	caacaagttagttgggtggg	1000 bp repair fragment of deletion
F168=Del-*IDH2*-RM-UF	ttgggtgactgatgtgtgtg	1000 bp repair fragment of deletion
F169=Del-*IDH2*-RM-UR	CGAAAACATATCCACATCCACAttatgtatgatttagatgatattatgac	1000 bp repair fragment of deletion
F170=Del-*IDH2*-RM-LF	GTCATAATATCATCTAAATCATACATAAtgtggatgtggatatgttttcg	1000 bp repair fragment of deletion
F171=Del-*IDH2*-RM-LR	ttctgccctgatatcgcgag	1000 bp repair fragment of deletion
F172=Del-*IDH2*-RM-FsnF	aaagggtatcacgtgtcgag	1000 bp repair fragment of deletion
F173=Del-*IDH2*-RM-FsnR	ggattctgtgtgcgcgttcg	1000 bp repair fragment of deletion
F196=*IDH1*-RM-F1	CTCGTCGACATCACTTTTTTTTCTTGTCGCCAACTTCGTGGAACCCCCAAAGAAATCACAACGATATAACGATAATGATAATGATTTGATGTAATGTTGGAAGCGATATGTGTGATCATC	Repair fragment for deletion

F197=*IDH1*-RM-R1	GATGATCACACATATCGCTTCCAACATTACATCAAATCATTATCATTATCGTTATATCGTTGTGATTTCTTTGGGGGTTCCACGAAGTTGGCGACAAGAAAAAAAAAGTGATGTCGACGAG	Repair fragment for deletion
F198=*IDH2*-RM-F1	CACACCCACTACTCATCAATAATTCGTTCAATGTCATAATATCATCTAAATCATACATAATGTGGATGTGGATATGTTTTCGATTAGACGACAAGATGTTGCCAAAAGGTGTCCTAGTTA	Repair fragment for deletion
F199=*IDH2*-RM-R1	TAACTAGGACACCTTTTGGCAACATCTTGTCGTCTAATCGAAAACATATCCACATCCACATTATGTATGATTTAGATGATATTATGACATTGAACGAATTATTGATGAGTAGTGGGTGTG	Repair fragment for deletion
F200=*ICL1*-RM-F1	CAAAGAAGTCGGTCTCACCAATGCAAGTGTCACATCAAACATCTGTCCCGTACTAACCCAGCAGTTTGTTTAGCAAAATATATTTAACGAGTTTGATAGAGGCGCTGGACTACATAATTA	Repair fragment for deletion
F201=*ICL1*-RM-R1	TAATTATGTAGTCCAGCGCCTCTATCAAACTCGTTAAATATATTTTGCTAAACAAACTGCTGGGTTAGTACGGGACAGATGTTTGATGTGACACTTGCATTGGTGAGACCGACTTCTTTG	Repair fragment for deletion
F259=User-p*BR30*-*AtCAD*-PF	CGTGCGAUcgaactgtgtaggaatgaaac	Promoter amplification
F260=User-p*BR30*-*AtCAD*-PR	ATTGTGGCggUTGTGTGTGGGTGTGTCGTTCG	Promoter amplification
F261=User-p*BR30*-*AtCAD*-GF	accGCCACAAUGACCAAGCAGTCTGCCGACTC	Gene amplification
F262=User-*ICL1*-*AtCAD*-PF	CGTGCGAUgtagcgttggtctgtcctgtcg	Promoter amplification
F263=User-*ICL1*-*AtCAD*-PR	ATTGTGGCUTGGGTTAGTACGGGACAGATG	Promoter amplification
F264=User-*ICL1*-*AtCAD*-GF	aGCCACAAUGACCAAGCAGTCTGCCGACTC	Gene amplification
F287=IDH1-promoter-cr1F	GAGCCGTGCGTGCAATTAGAgttttagagct	For construction of AKEC8 gRNA plasmids for IDH1 promoter changing
F288=IDH1-promoter-cr1R	TCTAATTGCACGCACGGCTCtaaccaacct	For construction of AKEC8 gRNA plasmids for IDH1 promoter changing
F289=IDH1-promoter-cr2F	GCGATTGGACGGGCTTTACAgttttagagct	For construction of AKEC8 gRNA plasmids for IDH1 promoter changing
F290=IDH1-promoter-cr2R	TGTAAAGCCCGTCCAATCGCtaaccaacct	For construction of AKEC8 gRNA plasmids for IDH1 promoter changing
F322=U1-4UASTef-PF	CGTGCGAUCGATACGCGTatcgatacgc	Promoter amplification
F323=U1-4UASTef-PR	ATTGTGGCUgtggatccttcgggtgtgag	Promoter amplification
F324=U1-4UAS/Tef-*AtMTT*-GF	aGCCACAAUGGACAGCAAGATTCAGAC	Gene amplification
F325=U1-4UAS/Tef-*AtMTT*-GR	CACGCGAUTTAGTTGGGCTGTGTCAGGAAC	Gene amplification
F326=U1-4UAS/Tef-UmMtt1-GF	aGCCACAAUGCCTCCTAGCGGCCGAAAGG	Gene amplification
F327=U1-4UAS/Tef-UmMtt1-GR	CACGCGAUTCAGCTCTCGGGACCAGCGAGC	Gene amplification
F328=U1-4UAS/Tef-AkCtp1-GF	aGCCACAAUGGCTACTTCCGAAAACGACAAG	Gene amplification
F329=U1-4UAS/Tef-AkCtp1-GR	CACGCGAUTCAAATGTATCGTCGCTCGGGG	Gene amplification
F344=U1-Tefin-PR	AGTACUGCAAAAAGTGCTGGTCGGATG	Promoter amplification
F346=U1-Tefin-*AtCAD*-GF	AGTACUAACCGCAGACCAAGCAGTCTGCCGACTC	Gene amplification
F347=U1-Tefin-*AtMTT*-GF	AGTACUAACCGCAGGACAGCAAGATTCAGACTAAC	Gene amplification
F348=U1-Tefin-Ummtt1-GF	AGTACUAACCGCAGCCTCCTAGCGGCCGAAAGGTG	Gene amplification

F349=U1-Tefin-AkCtp1-GF	AGTACUAACCGCAGGCTACTTCCGAAAACGACAAG	Gene amplification
F350=U1-Tefin-*AtACO*d-GF	AGTACUAACCGCAGGCTACCGTGGCCGACTCGCCC	Gene amplification
F351=U1-Tef-cox4/CAD-GSG-ACO/d-1R	AGGGGAGACUTTACACCAGGGGAGACTTCACG(wrongreorder)->	*AtACO* and *AtCAD* fused protein
F352=U1-Tef-cox4/CAD-GSG-ACO/d-2F	AGTCTCCCCUGGTGGGTTCTGGTGCTACCGTGGC CGACTCGCC	*AtACO* and *AtCAD* fused protein
F353=U1-Tef-ACO/d-GSG-CAD-1R	AGAACCGTUGGAGGCGGCCTTTCGAGCCATG	*AtACO* and *AtCAD* fused protein
F354=U1-Tef-ACO/d-GSG-CAD-2R	AACGGTTCUGGTATGACCAAGCAGTCTGCCGA	*AtACO* and *AtCAD* fused protein
F367=U1-4UAS/Tef-SceDIC1-GF	aGCCACAAUGAGCACAAATGCCAAGGAAAG	Gene overexpression
F368=U1-4UAS/Tef-SceDIC1-GR	CACGCGAUTCACTTGTCCTCCTTAGGCATG	Gene overexpression
F369=U1-4UAS/Tef-MmuSlc-GF	aGCCACAAUGGCTGAAGCCCGCACGTCGCG	Gene overexpression
F370=U1-4UAS/Tef-MmuSlc-GR	CACGCGAUTCAGGTCGTGGGAACCTTGATG	Gene overexpression
F371=U1-4UAS/Tef-AtDIC-GF	aGCCACAAUGGCTGAAGAGAAGAAAGCTC	Gene overexpression
F372=U1-4UAS/Tef-AtDIC-GR	CACGCGAUTCACATTCCAATCTTCTTCTG	Gene overexpression
F373=U1-Tefin-SceDIC1-GF	AGTACUAACCGCAGAGCACAAATGCCAAGGAAAGC	Gene overexpression
F374=U1-Tefin-MmuSlc-GF	AGTACUAACCGCAGGCTGAAGCCCGCACGTCGCG	Gene overexpression
F375=U1-Tefin-AthDIC-GF	AGTACUAACCGCAGGCTGAAGAGAAGAAAGCTCC	Gene overexpression
F400=p*IDH1*-UF	ttgctctgcagaagaagaac	*IDH1* deletion
F401=p*IDH1*-UR	cggctcggttttatatccgg	*IDH1* deletion
F402=p*IDH1*-LF	atgctcaaccttagaaccgc	*IDH1* deletion
F403=p*IDH1*-LR	ttgaaggttcgtcgcagagtc	*IDH1* deletion
F404=p*IDH1*-FsnF	gagtctatggctgggatctc	*IDH1* deletion
F405=p*IDH1*-FsnR	gacccttgagggcaaccttg	*IDH1* deletion
F426=*IDH1*-check-FF	tttagggaaaagccggtgggg	*IDH1* deletion
F427=*IDH1*-check-RR	gaccctgtactttgacagctc	*IDH1* deletion
F428=*IDH2*-check-FF	ctgttctcggttgttcaattg	*IDH1* deletion
F429=*IDH2*-check-RR	tcaggatcacgattttgatag	*IDH1* deletion
F430=4UASTef-seam-PF	CACGCGAUCGATACGCGTatcgatacgcg	4UASTef promoter
F431=4UASTef-seam-PF	ACCTGCACUtgtggatccttcgggtgtgag	4UASTef promoter
F432=4UASTef-seam-*AtMTT*-GF	AGTGCAGGUGCCACAATGGACAGCAAGATTCAGAC	*AtMTT* under 4UASTef promoter
F433=4UASTef-seam-*AtMTT*-GR	CGTGCGAUTTAGTTGGGCTGTGTCAGGAAC	*AtMTT* under 4UASTef promoter
F436=p*IDH1*-1A00683-ck1	TCTCAGAATCGCAATTTGCAGA	Check primer for*IDH1* promoter change
F437=p*IDH1*-1D13716-ck2	gctacttgtagtgggagaggg	Check primer for*IDH1* promoter change
F438=p*IDH1*-1B21134-ck3	ggtatgtatctctcctacccgg	Check primer for*IDH1* promoter change

F439=p*IDH1*-1F27529-ck4	cccgaagcgctgacatacttca	Check primer for*IDH1* promoter change
F440=p*IDH1*-1E16597-ck5	gatggactcccaggtgtacac	Check primer for*IDH1* promoter change
F441=p*IDH1*-1B10130-ck6	cctattctgcgagtgtcgtgg	Check primer for*IDH1* promoter change
F442=p*IDH1*-1D07348-ck7	catgcccacaacatgaacaag	Check primer for*IDH1* promoter change
F443=p*IDH1*-1C24124-ck8	ggcttgttccttgcaaccgag	Check primer for*IDH1* promoter change
F444=p*IDH1*-1F04643-ck9	tgtctagtcacgtgtaggtgg	Check primer for*IDH1* promoter change
F445=p*IDH1*-1A14199-ck10	ggttcttcgacaaagccacac	Check primer for*IDH1* promoter change
F446=p*IDH1*-1D13716-ampF	gtggtcattgaggtagacattg	Check primer for*IDH1* promoter change
F447=p*IDH1*-1D13716-ampR	cgtttacgatcttgagcgaagc	Check primer for*IDH1* promoter change
F509=U-pTef(20bp)-F	CGTGCGAUAGAGACCGGGTTGGCGGCGC	Tef promoter
F510=U-pTef(20bp)-R	ATTTTGAAUGATTCTTATACTCAGAAGG	Tef promoter
F535=U-UmAdi1-F	ATTCAAAAUGCTCCACCCCATCGATACTAC	Gene overexpression
F536=U-UmAdi1-R	CACGCGAUTCACGACAAGCTTCGGTCAG	Gene overexpression
F537=U-UmTad1-F	ATTCAAAAUGGCTCCCGCCCTTAACGCCAAC	Gene overexpression
F538=U-UmTad1-R	CACGCGAUTCAGGCAGAAGACGGGCGGCTAAG	Gene overexpression
F546=4UASTef-noseam-PF	CGTGCGAUCGATACGCGTatcgatacgcg	4UASTef promoter
F547=4UASTef-noseam-PR	ATtgtggaUccttcgggtgtgagttgac	4UASTef promoter
F548=4UASTefnosm*AtMTT*-GF	atccacaAUGGACAGCAAGATTCAGACTAAC	*AtMTT* under 4UASTef promoter
F549=4UASTefnosm*AtMTT*-GR	CACGCGAUTTAGTTGGGCTGTGTCAGGAAC	*AtMTT* under 4UASTef promoter
F550=4UASTefnosm*AtCAD*-GF	atccacaAUGACCAAGCAGTCTGCCGACTC	*AtCAD* under 4UASTef promoter
F551=4UASTefnosm*AtCAD*-GR	CACGCGAUTTACACCAGGGGAGACTTCAC	*AtCAD* under 4UASTef promoter
F552=Ri-*IDH1*F1U0	CGTGCGAUAGAGACCGGGTTGGCGGCGC	RNAi repeat
F553=Ri-*IDH1*F1D1	AAGCATGGUCACGGGAAAGGAGCCCTCCACCCTCACGTtcccgctcct tctccgagtcg	RNAi repeat
F554=Ri-*IDH1*F1D2	AAGCATGGUCACGGGAAAGGAGCCCTCCACCCTCACGTccaattattc cgctgcgtctc	RNAi repeat
F555=Ri-*IDH1*F1D3	AAGCATGGUCACGGGAAAGGAGCCCTCCACCCTCACGTttgaaggttc gtcgcagagtc	RNAi repeat
F556=Ri-*IDH1*F1D4	AAGCATGGUCACGGGAAAGGAGCCCTCCACCCTCACGTcttgagtcg cttgataatctg	RNAi repeat
F557=Ri-*IDH1*F2U1	ACCATGCTUCCGGGAGACGGTGTGGGGCCTGAGCTGATGtcccgctc cttctccgagtc	RNAi repeat
F558=Ri-*IDH1*F2U2	ACCATGCTUCCGGGAGACGGTGTGGGGCCTGAGCTGATGccaattatt ccgctgcgtctc	RNAi repeat
F559=Ri-*IDH1*F2U3	ACCATGCTUCCGGGAGACGGTGTGGGGCCTGAGCTGATGttgaaggt tcgtcgcagagtc	RNAi repeat
F560=Ri-*IDH1*F2U4	ACCATGCTUCCGGGAGACGGTGTGGGGCCTGAGCTGATGcttgagtc gcttgataatctg	RNAi repeat
F561=Ri-*IDH1*F2D0	CACGCGAUatgctcaaccttagaaccgc	RNAi repeat
F562=Ri-*IDH1*F1D0	ATTTGAATGAUTCTTATACTCAGAAGGAAATG	RNAi repeat
F563=Ri-*IDH1*F2U5	ATCATTCAAAUttcttgtcgccaacttcgtg	RNAi repeat
F564=Ri-*IDH1*F2D5	AAGCATGGUCACGGGAAAGGAGCCCTCCACCCTCACGTcgcaatccg catttaaaaag	RNAi repeat
F565=Ri-*IDH1*F3U5	ACCATGCTUCCGGGAGACGGTGTGGGGCCTGAGCTGATGcgcaatc cgcatttaaaaag	RNAi repeat

F566=Ri-*IDH1*F3D5	CACGCGAUtttcttgtcgccaacttcgtg	RNAi repeat
F567=Ri-*IDH1*F2U6	ATCATTCAAAUcaaccttagaaccgcccttc	RNAi repeat
F568=Ri-*IDH1*F2D6	AAGCATGGUCACGGGAAAGGAGCCCTCCACCCTCACGTattgtcccg ctccttctccg	RNAi repeat
F569=Ri-*IDH1*F3U6	ACCATGCTUCCGGGAGACGGTGTGGGGCCTGAGCTGATGattgtccc gctccttctccg	RNAi repeat
F570=Ri-*IDH1*F3D6	CACGCGAUtcaaccttagaaccgcccttc	RNAi repeat
F571=Ri-*IDH1*F2U7	ATCATTCAAAUgagtatctcggagcccggg	RNAi repeat
F572=Ri-*IDH1*F2D7	AAGCATGGUCACGGGAAAGGAGCCCTCCACCCTCACGTggtctctcat caccctcagttc	RNAi repeat
F573=Ri-*IDH1*F3U7	ACCATGCTUCCGGGAGACGGTGTGGGGCCTGAGCTGATggtctctcat caccctcagttc	RNAi repeat
F574=Ri-*IDH1*F3D7	CACGCGAUtgagtatctcggagcccggg	RNAi repeat
F575=Ri-*IDH1*F2U8	ATCATTCAAAUcgcaatccgcatttaaaaag	RNAi repeat
F576=Ri-*IDH1*F2D8	CACGCGAUtttcttgtcgccaacttcgtg	RNAi repeat
F577=Ri-*IDH1*F2U9	ATCATTCAAAUctacttgagtcgcttgataatc	RNAi repeat
F578=Ri-*IDH1*F2D9	CACGCGAUatgctcaaccttagaaccgc	RNAi repeat
F579=Ri-*IDH1*F2U10	ATCATTCAAAUgaaggttcgtcgcagagtcag	RNAi repeat
F580=Ri-*IDH1*F2D10	CACGCGAUtcgactgggaggttgtcgacg	RNAi repeat
F613=P1F-3*C&M	CGTGCGAUTTACACCAGGGGAGACTTCACG	JFEC127 and JFEC159
F614=P1R-3*C&M	ACCTGACACUCAGATGCATTCTTGGGCGGTC	JFEC127 and JFEC159
F615=P2F-3*C&M	AGTGTCAGGUACGCAACTAACATGAATGAATACG	JFEC127 and JFEC159
F616=P2R-3*C&M	ACAGGACTGUCAGATGCATTCTTGGGCGGTC	JFEC127 and JFEC159
F617=P3F-3*C&M	ACAGTCCTGUACGCAACTAACATGAATGAATACG	JFEC127 and JFEC159
F618=P3R-3*C&M	CACGCGAUTTAGTTGGGCTGTGTCAGGAAC	JFEC127 and JFEC159
F637=ampd-UF	CGTGCGAUTATACACAAGAACCAGCATG	Ampd amplification
F638=ampd-UR	AGTCGAGGCUATTGTATATGAGTATCAAGCTC	Ampd amplification
F639=AMPDTEF-PF	AGCCTCGACUAGAGACCGGGTTGGCGGCGC	Ampd amplification
F640=AMPDTEF-PR	ATTGCTTGCTGCGGCAUTTTGAATGATTCTTATACTC	Ampd amplification
F641=ampd-DF	ATGCCGCAGCAAGCAAUGGATATCAAGGGCAAGGCC	Ampd amplification
F642=ampd-DR	CACGCGAUGATAGACACAGGTAGGATCTC	Ampd amplification
F669=JFEC127-c1ckR	TTAGTTGCGTACCTGACACTC	Check primer for JFEC127 and JFEC159
F670=JFEC127-c2ckF	ATGCATCTGAGTGTCAGGTAC	Check primer for JFEC127 and JFEC159
F671=JFEC127-c2ckR	GTTAGTTGCGTACAGGACTGT	Check primer for JFEC127 and JFEC159
F672=JFEC127-c3ckF	GAATGCATCTGACAGTCCTGT	Check primer for JFEC127 and JFEC159
F729=AMPD-cr3F	AAGAGGGGCTAATAACCTGGgttttagagct	gRNA expression cassette
F730=AMPD-cr3R	CCAGGTTATTAGCCCCTCTTtaaccaacct	gRNA expression cassette
F731=AMPD-cr4F	TTGGAGATAGAGCCGTTGGAgttttagagct	gRNA expression cassette
F732=AMPD-cr4R	TCCAACGGCTCTATCTCCAAtaaccaacct	gRNA expression cassette
F781=q*IDH1*-F1	ttagaaccgcccttcgagctgtg	qPCR
F782=q*IDH1*-R1	ggttccgataccgtctccctcaatc	qPCR
F783=q*GAPDH*-F1	ggtattaacggattcggacgaatcgg	qPCR
F784= q*GAPDH* -R1	atgtaagcagcgtactcggtgtcg	qPCR

Table S6 The codon optimized genes used in this study

Genes	Coding sequences
AtCAD	ATGACCAAGCAGTCTGCCGACTCTAACGCCAAGTCTGGCGTGACCTCTGA<br>GATCTGCCACTGGGCCTCTAACCTGGCCACCGACGACATTCCCTCTGACG<br>TGCTCGAGCGAGCCAAGTACCTGATCCTGGACGGAATCGCCTGCGCCTGG<br>GTGGGCGCTCGAGTGCCCTGGTCTGAGAAGTACGTGCAGGCCACCATGTC<br>TTTCGAGCCACCTGGCGCCTGCCGAGTGATCGGCTACGGCCAAAAGCTGG<br>GCCCCGTGGCCGCTGCCATGACCAACTCTGCCTTCATCCAGGCCACCGAG<br>CTGGACGACTACCACTCTGAGGCCCCTCTGCACTCTGCCTCTATCGTGCTG<br>CCCGCCGTGTTCGCCGCCTCTGAGGTGCTGGCCGAGCAGGGCAAGACCAT<br>CTCTGGCATCGACGTGATCCTGGCCGCCATCGTGGGCTTCGAGTCTGGAC<br>CCCGAATCGGCAAGGCCATCTACGGCTCTGACCTGCTGAACAACGGCTGG<br>CACTGTGGCGCCGTGTACGGCGCTCCCGCTGGCGCCCTGGCTACCGGCAA<br>GCTGCTGGGACTGACCCCTGACTCTATGGAAGATGCCCTGGGAATCGCTT<br>GCACCCAGGCCTGCGGCCTGATGTCTGCCCAGTACGGCGGCATGGTGAA<br>GCGAGTGCAGCACGGCTTCGCCGCTCGAAACGGCCTGCTCGGAGGCCTG<br>CTGGCCCACGGCGGCTACGAGGCCATGAAGGGCGTCCTCGAGCGATCTT<br>ACGGCGGCTTCCTGAAGATGTTCACCAAGGGCAACGGCCGAGAGCCTCC<br>TTACAAGGAAGAGGAAGTCGTCGCCGGCCTGGGCTCTTTCTGGCACACCT<br>TCACCATCCGAATCAAGCTGTACGCCTGCTGTGGCCTGGTGCACGGCCCC<br>GTCGAGGCCATCGAGAACCTGCAGGGACGATACCCCGAGCTGCTGAACC<br>GAGCCAACCTGTCTAACATCCGACACGTCCACGTGCAGCTGTCTACCGCC<br>TCTAACTCTCACTGCGGCTGGATCCCCGAGGAACGACCCATCTCTTCTAT<br>CGCCGGACAGATGTCTGTGGCCTACATTCTGGCTGTGCAGCTGGTGGACC<br>AGCAGTGCCTGCTGTCTCAGTTCTCTGAGTTCGACGACAACCTTGAGCGA<br>CCCGAGGTGTGGGACCTCGCTCGAAAGGTGACCTCTTCGCAGTCTGAGGA<br>ATTCGACCAGGACGGCAACTGCCTGTCTGCCGGCCGAGTGCGAATCGAGT<br>TCAACGACGGATCTTCTATCACCGAGTCTGTCGAGAAGCCCCTGGGCGTG<br>AAGGAACCCATGCCTAACGAGCGAATCCTGCACAAGTACCGAACTCTGG<br>CCGGCTCTGTGACCGACGAGTCTCGAGTGAAGGAAATCGAGGACCTGGT<br>CCTGGGCCTCGACCGACTGACCGACATCTCGCCCCTGCTCGAGCTGCTCA<br>ACTGCCCCGTGAAGTCTCCCCTGGTGTAA
AtMTT	ATGGACAGCAAGATTCAGACTAACGTCCCTCTGCCAAAAGCACCCCTTAC<br>CCAGAAAGCCCGAGGCAAGCGAACCAAGGGCATTCCCGCTCTTGTTGCG<br>GGAGCGTGTGCTGGTGCCGTCGAGATTTCGATCACCTACCCCTTTGAGTC<br>CGCCAAAACCCGAGCCCAGCTTAAACGGCGTAACCACGATGTGGCGGCT<br>ATTCGACCGGGCATCAGAGGATGGTACGCTGGATATGGTGCAACACTCGT<br>TGGAACCACACTGAAGGCTTCTGTCCAGTTCGCCTCGTTCAACATCTATC<br>GGAGTGCATTGGCTGGACCGAATGGCGAGATCTCCACCGGTGCTTCGATG<br>CTGGCTGGTTTCGGAGCCGGAGTGACTGAAGCCGTTCTGGCTGTGACTCC<br>TGCCGAAGCCATCAAGACCAAGATCATCGACGCCCGAAAGGTTGGTAAC<br>GCAGAGCTGTCCACGACCTTTGGAGCCATTGCTGGCATTCTCCGAGACAG<br>AGGTCCATTGGGCTTCTTCTCTGCGGTTGGACCCACTATTCTCCGACAGTC<br>TAGCAATGCCGCCGTGAAGTTCACGGTGTACAACGAGCTCATTGGCCTGG<br>CCAGAAAGTACTCACACAACGGCGAAGATGTGCATCCTCTGGCCTCTACG<br>CTCGTTGGCTCTGTCACTGGTGTCTGTTGCGCATGGAGTACCCAACCTCTG<br>GACGTGATCAAGACACGTATGCAGTCTCTGCAAGCTCGACAGTTGTACGG<br>CAACACCTTCAACTGCGTCAAGACCCTCCTGCGGTCAGAGGGTATCGGCG<br>TCTTTTGGTCCGGAGTGTGGTTTCGCACTGGCCGACTTTCCCTGACTTCGG<br>CTATCATGTTTCCCGTGTACGAGAAGGTGTACAAGTTCCTGACACAGCCC<br>AACTAA
AkCtp	ATGGCTACTTCCGAAAACGACAAGAGATCGAAGCCATCGTCTTTGAGATC<br>TATCATCGCCGGTTCTACAGCAGGAGCGGTGGAGATTGCCATTACGTACC<br>CTGCCGAGTTCGCCAAAACTCGCTCACAACTCAACCGACGACTGCCCGAT

	GCCAAGAAGCTGCCCTGGCCCCCATTCGGTTCCCAGTGGTACGCCGGATG<br>TACCACTCTCATCATCGGCAATAGTCTCAAAGCCGGCATCCGGTTTGTGG<br>CCTTTGACTGGCTGAAATCTCTGCTGCAGGACGAGAACGGCCAGATTTCC<br>GGACCCAAGACCGTTATTGCGGGGTTTGGAGCTGGCTTTACCGAGTCTTT<br>GCTTGCGGTCACTCCTTTCGAGTCGATCAAGACACAGCTGATTGATGATC<br>GAAAGTCACAGAACCCCCGAATGCGAGGTTTTCTGCACGGTAGCCGAGT<br>GATATTCCAGGAGCGAGGTGTGCGTGGATTCTTCCAAGGGTTTGTCCCCA<br>CCACTGCTAGACAGGCTGCCAACTCTGCCACGAGGTTCTCCTCGTACACC<br>ATGCTAAAGCAGATGGCACAGGGCTATGTTGCTCCGGGAGAAAAGCTCG<br>GCACCGCTTCCACCTTTGCTCTGGGAGGCATCGCAGGCTTGATCACAGTC<br>TATGTAACGCAACCCCTTGACACCGTCAAGACCCGGATGCAGAGCCTGG<br>AGGCTTCCAAGAACTACAAGAATAGTTTTGTGTGCGCCGCACGCATTTTC<br>AAGGACGAAGGTATCTTCACCTTCTGGTCCGGCGCTGTTCCTCGACTCGC<br>CCGGCTAATCATGTCGGGAGGAATTGTGTTCACCATGTACGAGAAAACCA<br>TGGACGCTCTTGATGGTCTGGACCCCGAGCGACGATACATTTGA
Ummtt1	ATGCCTCCTAGCGGCCGAAAGGTGAGCCCGTCTGTATCTGTGGTGGCAGG<br>TGCTACTGCCGGCGCAGTGGAAGGTGTAGCCACGTTCCCCATCGAGTATT<br>TAAAAACAGTGTCACAATTTGCCCCTCGGGATGTGCACGGCAACCAGCA<br>GCGTTTGTCTCCCATTGAGGTAGTTAGGTCGACGCTGCAGAAGGAGGGCC<br>CCAAGGGTCTGTTCCGGGGCTGCACCGCCATGGTGGTCGGAAATGCTGGC<br>AAGGCTGGAGTTCGATTCTTCGCTTTTGAGAACTTCCGATCCATGCTGAA<br>GAACAAGTCCACCGGCAAACTCTCCAATTCTTCCAACTACCTCGCGGGCA<br>TGGGCGCTGGTACTTTGGAAGCGATCTTTGCCGTTACACCTTCCGAGACT<br>ATCAAGACCAAGCTGATTGATGACTCGAAGCGAGCCAAGCCCCGATACG<br>AACAAGGACTTGTGCGTGGCACTGCCTCTATTGTTCGTCAGGAGGGTCTG<br>GCCGGAATCTACCAGGGAGTCGTGCCGGTTGTCATGAGACAGGGGAGCG<br>CCTCAGCCATTAGACTTGGTACGTACTCGGCACTTCGCGACTGGTTGCCA<br>AAGGCGCATGGATCTGGATCATCGCTAATCAACTGGCTCGCTACATTTTC<br>GATAGGTGCTGCGAGTGGTGTCGTTGCTGTCTATGGAACCATGCCCTTCG<br>ACGTGCTCAAGACTCGAATGCAGGCCATCGACGCCGCAAGATACCGATC<br>TACCTGGCACTGTCTGACCAACACCCTGAAAACTGAGGGAGCAGCCGCTC<br>TGTGGCGAGGAAGTGTCTCGCGGTCTATGCGACTCATTGTCTCCGGAGGC<br>GTCATTTTTTCCGTCTACGAGCAGGTTGTGTGGCTGCTCGCTGGTCCCGAG<br>AGCTGA
SceDIC	ATGAGCACAAATGCCAAGGAAAGCGCTGGCAAGAACATCAAGTACCCCT<br>GGTGGTACGGAGGCGCAGCCGGTATCTTTGCTACCATGGTTACTCATCCT<br>CTGGACCTGGCCAAGGTGCGGCTGCAAGCTGCTCCCATGCCTAAACCCAC<br>CTTGTTCCGAATGCTGGAATCGATTTTAGCCAACGAGGGTGTGGTTGGCC<br>TTTACTCGGGCCTGTCTGCGGCCGTGCTGCGACAGTGCACCTACACTACT<br>GTTCGGTTTGGAGCCTATGATCTTCTTAAGGAGAACGTCATCCCCCGAGA<br>GCAGCTCACCAATATGGCCTACCTGCTCCCGTGCTCCATGTTCTCCGGAG<br>CAATTGGAGGACTAGCGGGGAACTTCGCCGACGTTGTCAACATTCGTATG<br>CAGAACGACTCTGCTCTGGAGGCCGCCAAACGTCGAAACTATAAAAACG<br>CCATAGATGGAGTCTACAAGATCTACAGATACGAGGGCGGTCTGAAAAC<br>TCTGTTCACCGGCTGGAAGCCCAACATGGTGAGAGGTATTCTCATGACCG<br>CATCTCAGGTGGTGACATACGACGTGTTCAAGAATTATCTCGTCACCAAG<br>CTCGACTTTGACGCCTCCAAGAACTACACCCATCTGACTGCCTCTCTTTTG<br>GCTGGACTTGTGGCTACGACGGTATGTTCGCCTGCAGATGTCATGAAGAC<br>CCGGATCATGAACGGCTCAGGCGACCACCAGCCCGCGTTGAAGATTCTG<br>GCTGATGCTGTTCGAAAAGAAGGCCCATCTTTCATGTTTCGAGGATGGCT<br>GCCTAGTTTTACGCGCTTGGGTCCATTTACAATGCTCATCTTCTTCGCCAT<br>TGAGCAACTCAAGAAGCACAGAGTCGGCATGCCTAAGGAGGACAAGTGA
MumSlc	ATGGCTGAAGCCCGCACGTCGCGATGGTACTTTGGAGGCCTGGCGAGCTG<br>TGGAGCTGCCTGCTGTACTCATCCTTTGGATTTACTCAAGGTCCACCTGCA<br>GACCCAGCAGGAGGTCAAGCTGAGGATGACTGGTCTGGCTCTTCAGGTG

	GTTCGTACAGATGGATTTCTTGCCCTGTATAATGGTCTATCTGCATCGCTC<br>TGCCGACAGATGACCTATTCACTCACACGGTTTGCCATCTACGAGACTAT<br>GCGAGACTACATGACAAAGGACTCTCAAGGCCCCCTGCCCTTCTACAATA<br>AGGTGCTACTGGGCGGTATTTCCGGCCTTACTGGTGGTTTTGTGGGTACG<br>CCTGCAGATTTGGTCAACGTGCGAATGCAGAACGACATGAAGCTTCCTCC<br>TTCACAAAGACGGAACTACTCCCATGCTCTGGACGGACTCTACCGAGTTG<br>CACGAGAGGAGAGCCTGAGAAAACTCTTCTCCGGAGCCACCATGGCGTC<br>CAGTCGCGGCGCTCTCGTAACAGTGGGCCAGCTCTCGTGCTACGACCAGG<br>CCAAGCAGCTGGTTTTGTCTACTGGATATCTTTCTGACAACATCTTCACCC<br>ATTTTGTGAGTTCTTTCATCGCTGGCGGGTGTGCTACCTTTCTGTGCCAGC<br>CCCTCGATGTGCTTAAGACCAGACTCATGAACTCCAAAGGAGAATACCA<br>GGGAGTCTTCCACTGTGCCATGGAGACTGCCAAACTCGGTCCGCAAGCCT<br>TCTTCAAGGGCCTGTTCCCAGCTGGAATTCGACTGATTCCCCACACCGTC<br>CTGACTTTCATGTTCCTGGAGCAGCTCCGGAAGCACTTTGGCATCAAGGT<br>TCCCACGACCTGA
AthDTC	ATGGCTGAAGAGAAGAAAGCTCCAATTTCTGTGTGGACCACCGTCAAGC<br>CCTTTGTCAACGGCGGTGCATCGGGCATGCTTGCTACGTGTGTCATCCAG<br>CCCATTGACATGATCAAGGTGCGAATCCAGCTCGGCCAGGGTTCTGCTGC<br>CTCCATTACAACCAACATGCTCAAGAACGAGGGCGTTGGAGCATTCTACA<br>AGGGACTTAGCGCCGGTCTTCTGCGGCAGGCCACTTATACGACTGCTCGT<br>CTGGGATCGTTCAAGCTCCTGACTGCCAAGGCCATTGAGTCCAACGACGG<br>CAAACCGCTCCCTCTGTACCAGAAAGCTCTGTGCGGTCTGACCGCAGGCG<br>CAATCGGTGCCTGTGTTGGCTCTCCTGCTGATCTGGCACTCATCCGGATGC<br>AGGCTGACAACACTCTGCCTCTAGCGCAGCGACGAAACTACACAAATGC<br>CTTCCACGCCTTGACGAGAATCTCCGCGGACGAGGGAGTGCTTGCCCTGT<br>GGAAGGGTTGTGGCCCCACAGTGGTTCGCGCTATGGCCCTCAACATGGGC<br>ATGCTGGCTTCCTACGACCAGAGCGCTGAGTACATGCGAGATAATCTGGG<br>GTTTGGAGAAATGTCCACCGTTGTCGGAGCCAGTGCCGTATCTGGATTCT<br>GCGCAGCCGCCTGCTCGTTGCCGTTTGACTTTGTGAAAACACAGATTCAA<br>AAGATGCAACCAGATGCGCAAGGCAAGTACCCCTACACCGGATCTCTGG<br>ATTGTGCCATGAAGACTCTCAAGGAGGGTGGTCCTCTCAAGTTTTATTCA<br>GGCTTCCCCGTCTACTGCGTGAGAATTGCCCCCCATGTCATGATGACCTG<br>GATCTTCTTGAACCAGATCACCAAGTTCCAGAAGAAGATTGGAATGTGA
UmTad1	ATGGCTCCCGCCCTTAACGCCAACCCCACCACCAAGAGGGACGAGCTCA<br>GTGCCCCTTCTGCTTCGCATAAGCTCGGGATGTCGTCCATGGCATCTCGA<br>GCTGCTGGAGGAGGCCTCAAGCTCACCGGACTCCCCGACCTGTCCGACTC<br>AGCCGGTACTCTGTCTGACATTTTCGGTACACCTCAGATGCGAGAAATTT<br>GGTCCGATCAGAACCGTGTTGCCTGCTACCTCGAGATCGAGGCAGCCCTT<br>GCCATTGTGCAGGCCGATCTGGGTATCATTCCCAAGAACGCAGCACATGA<br>GATAGTCGAGCACTGTCGGGTCCAAGAGATTGACTGGGCCCTATACAAG<br>CAGAAGACCGAACTCATTGGATACCCGGTGCTCGGGATCGTCCAGCAGCT<br>GGTGGCCAACTGCAAGGACGGACTGGGTGAGTACTGCCACTGGGGCGCC<br>ACAACCCAGGACATCACTGACACAGCTACTGTAATGCAGATTCGACAATC<br>CCTGACCCTTGTCAAGCAGCGACTCGATTCGATCGTTTCGTCTCTGGAAC<br>ATCTGGCTGAGCAGCATCGAAACGTCCCCATGGCCGCCAGATCTAATCTG<br>AAACAGGCCGTGCCTATCACCTTTGGCTTCAAGATGGCGCGGTTCCTCGC<br>AACGTTTCGAAGACACCAGCAACGGCTGGTCGAGCTCGAAAAGCGAGTG<br>TACACCCTGGAGTTTGGAGGAGCTGCAGGAAACTTGTCCTCACTCGGCGA<br>CCAGGGGATTGCCACACACGACGCTCTAGCCAAGATGCTGGATTTAGCTC<br>CTGCTGAAATTGCGTGGCACACTGAGCATGACCGTTTCGCCGAAGTTGGC<br>ACGTTCCTGGGCCTTCTTACGGGTACGCTCGCGAAATTGGCGACTGATAT<br>CAAGCTCATGTCTCAAACTGAAGTTGGAGAGGTGGGCGAGCCCTTCATCT<br>CCAACCGCGGCTCGAGCTCTACCATGCCCCAGAAAAATAATCCCATTTCC<br>TGTGTGTATATTCACGCCTGTGCTGCCAATGTGCGACAGGGCGCTGCGGC<br>TCTGTTGGACGCCATGCAAAGCGATCATGAGCGTGGAACTGGTCCCTGGG

	AGATAATCTGGGTCCAACTACCACTGATGATGAACTGGACCAGTGCTGCC TTGAACAACGCTGACTTTGTGCTTCGAGGCCTGCAGGTGTTCCCAGACGC TATGCAGCACAACCTCGATCTTTCGAAGGGTCTGATCGTCTCAGAGGCCG TCATGATGGGACTCGGTAACACCCTGGGCAGACAGTACGCCCACGATGC GGTATATGAGTGCTGTCGGACTGCTTTTGTTCAGGATCGACCTCTATTGG ATGTGTTGCTGGAAAACCACGAGATTGCTTCAAAACTCGACCGAACCGA GCTTGAGAAGCTGTGTGACCCTGCCAACTACCTGGGCCAGTGCTCGCAAT GGATCGACAGAGTTCTTAGCCGCCCGTCTTCTGCCTGA
UmAdi1	ATGCTCCACCCCATCGATACTACCATCTACCGAGCTGGAACGTCGAGGGG TCTGTACTTTCTGGCAAGTGATCTGCCAGCCGAGCCCAGCGAACGTGACG CCGCTTTGATTTCCATCATGGGCTCCGGCCATCCTCTGCAGATTGACGGTA TGGGAGGCGGAAACTCCCTCACCTCCAAGGTCGCCATTGTCTCTGCGTCC ACACAAAGATCTGAATTCGACGTGGACTACCTGTTCTGCCAGGTGGGTAT CACCGAGCGGTTTGTCGATACTGCTCCCAACTGTGGCAATCTCATGTCCG GAGTGGCCGCCTTTGCCATCGAGCGAGGTCTTGTCCAGCCCCATCCCAGT GATACAACCTGTCTGGTACGAATCTTCAACCTCAACTCTCGTCAGGCCTC TGAGCTGGTTATCCCCGTCTACAACGGCCGAGTCCACTACGACGACATTG ATGACATGCACATGCAGCGACCTTCTGCAAGAGTTGGTCTGCGGTTCCTG GACACTGTTGGGAGTTGCACCGGTAAGCTCCTACCCACAGGCAACGCGTC AGACTGGATCGACGGCCTCAAGGTGTCGATCATCGACTCCGCAGTCCCTG TGGTTTTCATCCGACAGCATGACGTTGGTATAACGGGGTCAGAGGCCCCG GCCACACTCAATGCCAACACCGCTCTGCTTGACCGGTTGGAGCGCGTTCG ACTCGAAGCAGGAAGACGAATGGGTTTGGGAGACGTGTCTGGATCTGTG GTGCCCAAACTGTCACTGATTGGCCCTGGAACTGAGACTACCACATTCAC TGCGCGGTATTTCACCCCCAAGGCCTGCCACAACGCACATGCCGTCACTG GCGCTATTTGTACTGCCGGTGCGGCCTACATTGATGGATCGGTAGTCTGC GAGATTCTCTCGAGCCGGGCTTCGGCGTGCTCTGCCTCTCAACGACGAAT TTCCATTGAGCACCCTTCGGGCGTTTTGGAGGTCGGACTAGTCCCACCCG AGAACGCTGCCCAGTCGCTCGTGGATGTGGCTGTTGTTGAGCGTAGCATT GCCCTTATTGCCCATGCTCGAGTGTACTACACCACACCGGACAGACGCCG TTCCTACGATTCCCCGCTGACTAGTCCCTCCACCCCTGCTGACACCCACAA TCTGTTTGATGCAGCTTATCGCCCTGTCATCCAGCCATCGGATACCGATGT GGAGGCTCCTCATATGCTGGCCCTTGAAAACAAGGAACAGTGTGTGTCTC GATGTGACACGGCACTTCACCACATCGTGGCGAGCTATGGCGCCAGCGA CGCTCACGCCTCTGACCGAAGCTTGTCGTGA
ScDcr1	ATGAACCGAGAGAAGTCTGCCGACCTGTCTAAGACCTCTGACGCTCCCTA CAACGAGCTGGACGCCAAGAACCTGCGAAACTTCTACAAGGTGCAGAAC GCCTGCGCTCAGCTGCGAGAGTCTATCAAGGTGATCTACGAGAACGGCCT GTCCTCTGACCAGCTGAACACCATGGCCAAGCACGGCAACGACCTCGAG AAGTCTATCGCCAACTCTCCCGCCATGTCTGTGGCCTCTTGCCTGAACCA GGTGCGACCCACTCTGGACATCAAGAACATCTTCGACCACTACAAGTTCG AGAACAACTCTTCTCCTGTGGACCCCTACGTGCACTACCCCGTGTGTTCTG ACCAGAACCTGGAAAACCTGGCCTTCATCCACCGATCTCTGCCCAACATG AACGTGAAGCTGACCGAGCTGCAAAAGACCGTGATGTCTAACGAGCGAC TCGAGTTCCTGGGCGACTCTTGGCTGGGCGCCCTGGTGGCCTACATCATC TACAAGAAGTACCCCTACGCCAACGAGGGCGCTCTGTCCAAGATGAAGG AAGCCATCGTTAACAACAACAACCTTGAGAAGATCTGCGAGAAGCTGGG CTTCAAGGAACGACTGAAGGAAAACATTCCCCGATCCTCTATGAAGATTA AGGACCGACTGACCAAGAACTACGCCGACTGCGTCGAGGCCTACATTGG AGCCCTGGTGATCGACCGATTCTCTACCGAGTTCAACGACGTGGCCCTGT GGCTCGAGGAACTGTCTGAGGAACACTTCATCGAGCTGGGCCCCATGATG GTGAAGGAACCCCTGAACAAGAACGCCAAGGGCGAGCTGGGAGCCTTCC TGCAGTTCAACAACATCGGCGCCAAGATCTCTTACAAGCGACTGAACGAC AAGTCGCCCTTCAAGGTCGAGGTGCGACTGGGCAACAACCTGCTCGGCAT CGGCGACGGCTCTAACGTGCGAGAGGCCGAGCAGCGAGCCGCCATGGAA

	GCCCTGGCTCAGCCCAAGCTGATCCAGAAGTACTCTCTGCACGACATCGA<br>GCTTGAGCGAGGCATGATCGAAGAGGCCGACAACGTGCCCCAGCTGCTG<br>AAGTCCGCCGAGATTCCCAACCAGACTCCTCAGTCTCACCACTCTATCAC<br>CGAGGAAATCGACGAGGAAGAGGGCCTGACCTTTCCTCACTCTAACCCAT<br>TTCTGAAGGGCACCCCTCCTGCCGTGCTGCCCGAGATCGTGACTAACCAG<br>ATTCCACAGGCCTCCGCCGACCACCAAGAGCAGAACGTGCCCGACACCG<br>ACAAGATCGTGAACGACGTGATGGAACGAATGTCTAAGATCCTGTCTGTG<br>ATGGTGTCTGAGGCCGTGTCTAACGCCCTGGGACGACCCGCTCCTAAGAA<br>CGCTCCCATTCCTATTCCAGTGACTACTCCCGTGTCTCCCCTGGCCACCTC<br>TCCTGTGTCCGTGTCTGTGCCCGCTCCTGCTCCTGCTGTGGCCGTGACTCC<br>CTCTGTGGTGAACAACCCTCCTCAGCCTAAGATGGTGACCCAGCCTGTGT<br>CTACCAACCTCAACAAGACCCCTGAGGTGGCCCTGCTGAACTCTGTGTAC<br>GACAAGGAAGCCTCTGGCCGACTGTACGCCCTGCTGGGCAAGTACAAGC<br>TGTACCCCGAGTACAACACCGAGCAGCTCGGCATGACCGACTTCTACACC<br>GTGTGCTTCATCAAGGGCGTGGGCGTCGAGATCGGCAAGGGCCACGGCC<br>GATCTAAGAAGATCTCTCAGCACAAGTCTGCTGAGGACGCCCTGAACGG<br>CAAGGCCCTGAAGGAATACCTGCGACACTGTAACAACCTGTAA
ScAgo1	ATGTCCTCTAACTCTGAAGAGAACTCTCAGGTGCCTCCTCTGGACGCCAC<br>CGCCGCTGCCACCAAGCCTAAGAAGGCCAAGAAGCCCAAGGTTAAGAAG<br>CCTAAGGACTCTGCCGAGGCCTCTTCTTCGCCCGCTGCCGAGGGCACCGC<br>CGAGGCTAAGCCCAAGAAGGCTAAGAAGTCTAAGACCAAGAAGTCGAAG<br>GAATCCGCCGAGGTGTCTCCCGCTCCTGCCGACGAGACTACCTCTGCCGG<br>CGTGGACGCAAAGCCAAAGAAGGCAAAGAAGAGTAAGGTCAAGAAGCC<br>AAAGGATTCCACCGAGTCCTCGCCTGCTCCTTCTAACGAGCCTCCTGCCG<br>CTGAGGTGGCCGCCGAGGACGCCAAGTCCAAGAAGGTGAAGAAGCCGAA<br>GGCAAAGAAGCCGAAGGAATCTACTGAGTCGAGCCCCGCTCCAGGCCAA<br>GAGGCTGCCGCCACCGAGGGCGCTACCGAGGATAAGCCTAAGAAGGTGA<br>AGAAGAGTAAGGCTAAGAAGGCCAAGGAATCTGTCGAGTCGTCCCCTGC<br>TGCTACCGAGTCTGTGTCTGAAAAGACCGCAAAGAAGTCGAAGAAGCCC<br>AAGGCCAAGAAGTCTACCTCTCCTGAGACTACCGAGGAAATCACCGAAG<br>AGTCTACCGAATCTAAGGAAAAGAAGACCAAGACAAAGAAGCCCAAGG<br>AAAAGAAGAGTTCTCCCTCTACCGCCACCTCCACCGCTGCCTCTAAGCCC<br>GTGACCTCTATCGCCGGCGTGACCATTCCTGGCAAGACCTTCGACCTGAC<br>CGACGTGCCTCCTTCGAAGCCCGCTCCTAAGATGGTGCCCGAGGCCTACA<br>AGCTGCAGACCCGAGTGGACTACGGCACCAAGGGCACTAAGGTGGACGT<br>GCTGACCAACCACATCCTGCTGTCTGTGGGCGACGACGTGCCCCAGGACG<br>AGCGAGCCTCTCAGCTGGACCCCTGGTGGAAGTCTGCCTTCGTGTACACC<br>TACAACATCACCTTCGCTGTGCCCCAGTCTAACTCTCCCCGAAAGGGCCC<br>TGCTCCTGCTCTGTCGAAGCCGAAGAAGTACGAGCTGGTCGAGTCTCTGT<br>TCCACGAGGACGAGACTCTGTTCAAGTACAAGGACCGAATCTCTTTCAAC<br>GGCGAGGACACCCTGTACTCGCACGTGCCCCTGGAAGAGTTTACCCTGTT<br>CGACGGCTGCTGGGACGTGTCTAACAAGCAGAAGAAGCGACGACAAGAG<br>GTCGTGGGCCTGAACTCTCGAGCCAAGGAAATCAACGACCTGGCCGCTC<br>AGGTGACCCTGAAGTTCGCCGACAAGGTGCCTCTGGGCGACATCTACAA<br>GGCCACCACCTCTAAGGACCCCGAGGAACAAGAGAACAAGATGGCCAAC<br>GCTGACAAGGTGGCCCTGCTGTCCCTGATGGGCGTGAAGTTCCTGAACAC<br>CAAGGAACAGATCTTCCAGCTGAACGGCAACAAGTTCTTCATCTTCAACG<br>AGCACGCCATTGCTACCCCTTTCCAGATCGGCGGCTTCCTGATGCACGGC<br>TTCACCGTGTCTCTGCGATACGCCTACGGCTCTGTGCTGCTGAACACCGT<br>GAACGTGTGTCTGCCCTTCGTCAAGTGGACCAAGTACCTGCCTGGCGACG<br>CTAAGTTCAAGGAAAACGAAAAGACCCAGTACTCTCTGCTGGACTGGAT<br>CATCGAGTGTATGCACCAGGCCTCTGCTCAGCGAGGCCAGAAGCTGCGA<br>GGACCTCCTTCGGCCAAGGACATTAACTTTTTCATCGACAAGAACCGAGA<br>CATCAAGGACCTGCTGAAGGGCCTGAAGTGCTACCGACCTTACATCAACT<br>ACTCTGTGAACCCCGACGGCACCCCTAAGCCTCCAAAGAAGATGCAGGC

	CAAGGGCATCGTGGGCTTCGTGCGAGAGACTCCCGACTCTATGAAGTTTC<br>GAACCCTGCCTTCTAACATGGAAAAGAACGGCGTGCCCAAGCCTGGCGA<br>GAAGGAAATTATGGTCACCACCACCGCCTACTTCGCCAAGAAGTATGAC<br>ATCAAGCTGAAGTACCCCGACGTGAAGATGGTGTCTCTCGGCGGCTCTAA<br>CGTGGTGCCCGCCGAGTGCCTGACCATCGTGCCCGGCCAAAAGCTGAAG<br>GGACTCGTGTACGACGAGAAGGCCGTGATCGACTTCACCGCTCTGCGACC<br>CTCTGAGAAGTTCCGAGCCATCACCAACCTGGCTCTGCCCGCCATCAAGC<br>GAGCCCTGTCCACCGAGGAAGAGAACGCCAAGGCTCCCCACGACTCTGG<br>CTACACCTTTATGAAGGTGCCCTCTCGAGTGATCGACGCTCCCGTGGTGC<br>AGTTCAAGAACACCACCGTGACCTACGTGGACAAGCCCTTTGGCACCAA<br>GAACGGCAAGAACAACCACGAGGAAACTAAGGGCAACTGGAACCTGAA<br>GGACCACAAGTTCATCACCGTGCCTAAGGAACCCATGCATCTGCGAGCCA<br>TCTTCATCAACGACTCCGACAAGTCTCCTCCTGTGTCCGTGATGGACGAG<br>CTGAAGGCCTCTCTGTCTAAGTTCGCTGAGGACGTGGCCGACGTGGGAGT<br>GAACTTCGACGTGTCCATGGCTCCCATCCTGATCAACAACTTCAACGCTC<br>CCATCAAGAAGGTCACCGGCGGTTTCGGAGGCCGAGGCGGCCGAGGTGG<br>AAGAGGCGGAAGAGGTGGTCGAGGCGGCAGAGGCGGCCGAGGCGGATT<br>CGGCGGAGGACGAGGCGAAACCACCTACGAGCTGACTCCCGGCGAGGAA<br>AAGCTGCGACATCTGCTGGCCAACGTGCCCGAAAAGACTTACGTGCTGTT<br>CGTGCTCGGACGAGGTGACGACTCTGCCATCTACAACCGACTCAAGTACC<br>TCGCTGACCTGACCTACGGCGTGATTAACAACTGCGTGATCTGGAACAAG<br>TTCCGAAAGTGCTCTACCCAGTACAACGTGAACGTCGTCATGAAGATGAA<br>CCTCAAGCTCGAGGGCGCCAACCACTCTCTGTGTGCCGAGGACATCAACC<br>TGCTCAAGGACGAGAAGTCTGGACTGCCCTTCATGATCCTGGGAGCCGAC<br>GTGACTCACTACCCCGAGAAGGACCAGAACTCTATCTCTGCCCTGGTGGG<br>CTCTTTTGACGACAAGTTCGCTCAGTTCCCCGGCTCTTACATGCTGCAGTC<br>TGGACCCGGCGAAGAGATCATTGCCGGCATCGGCAACATGGTGCTGCAG<br>CGACTGAAGCTGTACCAGAAGCACAACAACGGCAAGCTGCCACCTAAGA<br>TCCTGTTCTACCGAGATGGCGTGTCTGAGTCTCAGTTCTCTCAGATCGTGC<br>AGATCGAGGTCAAGGGACTGAAGCAGGCCCTGAAGAAGTTCGGCTCTGA<br>GCTGAACAAGGGCGTGAACTACAACCCCTCTGTGACCACCATCTGCGTGG<br>TGAAGCGAAACCAGATCCGATTCATGCCCCTCGAGCAGAACGCCATTAA<br>CGAGAAGGGCGAAGTCGCCGCCGTGCAGTCTTTCGAGAACGTGATGCCC<br>GGCACCGTGGTGGACCGAGGAATTACCTCTTCTGCCCACTTCGATTTCTTT<br>CTGCAATCTCAACAGCCCCTGAAGGGCACCGGTGTGCCCTGCCACTACTG<br>GTGCATCTACGATGAGAACCAGTTCAACTCTGACTACCTGCAGCAGGTCA<br>CCCACGCTCTGTGTTACCTGTTCGGCCGATCTTCTACCTCTATCAAGGTGG<br>CTTCTCCCGTGTACTACGCCGACCTGCTGTGCGAGCGAGGCGCCGCTTTC<br>TTCAAGGCCAACTTCGAGCTGGCCCAGTACGAGTTCTCTAAGGAACGAAA<br>GAACCGGGACGACGTGATCCCCACCGGAAAGCTGCTGCAGCCCGTGCAC<br>AAGAACGTGACCGACATCATGTACTACATCTAG
dCpf1<br>(Fncas12a^{D917A})	ATGTCCATCTACCAAGAGTTCGTGAACAAGTACTCTCTGTCTAAGACCCT<br>GCGATTCGAGCTGATTCCCCAGGGCAAGACCCTGGAAAACATCAAGGCC<br>CGAGGCCTGATCCTGGACGACGAGAAGCGAGCCAAGGACTACAAGAAGG<br>CCAAGCAGATCATCGACAAGTACCACCAGTTCTTCATCGAAGAGATCCTG<br>TCCTCTGTGTGCATCTCTGAGGACCTGCTGCAGAACTACTCTGACGTGTA<br>CTTCAAGCTGAAGAAGTCTGACGACGACAACCTGCAGAAGGACTTCAAG<br>TCTGCCAAGGACACCATCAAGAAGCAGATCTCTGAGTACATCAAGGACT<br>CTGAGAAGTTCAAGAACCTGTTCAACCAGAACCTGATCGACGCCAAGAA<br>GGGACAAGAGTCTGACCTGATTCTGTGGCTGAAGCAGTCTAAGGACAAC<br>GGCATCGAGCTGTTCAAGGCCAACTCTGACATCACCGACATCGACGAGG<br>CCCTTGAGATCATCAAGTCTTTCAAGGGCTGGACCACCTACTTCAAGGGA<br>TTCCACGAGAACCGAAAGAACGTGTACTCTTCTAACGACATCCCCACCTC<br>TATCATCTACCGAATCGTGGACGATAACCTGCCTAAGTTCCTTGAGAACA<br>AGGCCAAGTACGAGTCTCTGAAGGACAAGGCTCCCGAGGCCATCAACTA

CGAGCAGATCAAGAAGGACCTGGCCGAGGAACTGACCTTCGACATCGAT
TACAAGACCTCTGAGGTGAACCAGCGAGTGTTCTCTCTGGACGAGGTGTT
CGAGATCGCCAACTTCAACAACTACCTGAACCAGTCTGGCATCACCAAGT
TCAACACCATCATCGGCGGCAAGTTCGTCAACGGCGAGAACACCAAGCG
AAAGGGCATCAACGAGTACATTAACCTGTACTCTCAGCAGATTAACGAC
AAGACCCTCAAGAAGTACAAGATGTCCGTCCTGTTTAAGCAGATTCTGTC
TGACACCGAGTCTAAGTCTTTCGTGATCGACAAGCTTGAGGACGACTCCG
ACGTGGTGACCACCATGCAGTCTTTCTACGAACAGATCGCCGCCTTCAAG
ACCGTGGAAGAGAAGTCTATCAAGGAAACCCTGTCTCTGCTGTTCGACGA
CCTGAAGGCCCAGAAGCTGGACCTGTCCAAGATCTACTTTAAGAACGAC
AAGTCTCTGACCGACCTGTCTCAGCAGGTTTTCGACGACTACTCTGTGAT
CGGCACCGCCGTGCTTGAGTATATCACCCAGCAGATCGCCCCTAAGAACC
TGGACAACCCCTCTAAGAAGGAACAAGAACTGATCGCCAAGAAGACCGA
GAAGGCTAAGTACCTGTCGCTGGAAACCATTAAGCTGGCCCTGGAAGAG
TTCAACAAGCACCGAGACATTGACAAGCAGTGCCGATTCGAGGAAATCC
TGGCCAACTTCGCCGCTATTCCCATGATCTTCGACGAGATTGCCCAGAAC
AAGGACAACCTGGCTCAGATCTCTATCAAGTACCAGAACCAGGGAAAGA
AGGATCTGCTCCAGGCCTCTGCCGAGGACGACGTCAAGGCCATTAAGGA
CTTGCTGGACCAGACCAACAACCTGCTCCACAAGCTCAAGATCTTCCACA
TCTCTCAGTCTGAGGATAAGGCCAACATCCTGGACAAGGACGAGCACTTC
TACCTGGTGTTCGAAGAGTGCTACTTCGAGCTGGCCAACATTGTGCCCCT
GTACAACAAGATCCGAAACTACATCACCCAGAAGCCTTACTCGGACGAG
AAGTTTAAGCTGAACTTCGAGAACTCTACCCTGGCTAACGGCTGGGACAA
GAACAAGGAACCCGACAACACCGCCATCCTGTTTATCAAGGACGATAAG
TACTACCTGGGCGTGATGAACAAGAAGAACAACAAGATTTTTGACGACA
AGGCAATCAAGGAAAACAAGGGCGAGGGCTACAAGAAGATCGTCTACA
AGCTGCTGCCCGGTGCCAACAAGATGCTGCCTAAGGTGTTCTTCTCGGCC
AAGTCCATCAAGTTCTACAACCCTTCTGAGGACATCCTGCGAATCCGAAA
CCACTCTACCCACACCAAGAACGGCTCTCCCCAGAAGGGCTACGAAAAG
TTCGAGTTTAACATCGAGGACTGCCGAAAGTTCATCGACTTCTACAAGCA
GTCCATCTCTAAGCACCCCGAGTGGAAGGACTTTGGCTTCCGATTCTCTG
ACACCCAGCGATACAACTCTATCGACGAGTTCTACCGAGAGGTGGAAAA
CCAGGGATACAAGCTCACCTTTGAGAACATCTCCGAGTCTTACATCGACT
CTGTGGTCAACCAGGGTAAGCTGTACCTGTTTCAGATCTACAACAAGGAC
TTCTCTGCCTACTCTAAGGGACGACCCAACCTGCACACCCTGTACTGGAA
GGCCCTGTTTGACGAGCGAAACCTCCAGGACGTGGTGTACAAGCTGAAC
GGCGAGGCTGAGCTGTTTTACCGAAAGCAGTCGATTCCCAAGAAGATTAC
TCACCCCGCCAAGGAAGCTATCGCCAACAAGAACAAGGATAACCCCAAG
AAGGAATCTGTTTTCGAGTACGACCTGATCAAGGACAAGCGGTTTACCGA
GGACAAGTTCTTCTTCCACTGTCCTATCACTATCAACTTTAAGTCCTCTGG
CGCTAACAAGTTTAACGACGAGATCAACCTGCTGCTTAAGGAAAAGGCT
AACGACGTCCACATCCTGTCTATCGCCCGAGGCGAGCGACACCTGGCCTA
CTACACCCTGGTGGACGGCAAGGGCAACATCATCAAGCAGGACACCTTC
AACATCATTGGCAACGACCGAATGAAGACCAACTACCACGACAAGCTGG
CCGCCATCGAGAAGGACCGAGACTCTGCCCGAAAGGACTGGAAGAAGAT
CAACAACATCAAGGAAATGAAGGAAGGCTACCTCTCTCAGGTGGTGCAC
GAGATCGCTAAGCTGGTGATCGAGTACAACGCCATCGTGGTGTTCGagGA
CCTCAACTTCGGCTTCAAGCGAGGCCGATTCAAGGTCGAGAAGCAGGTCT
ACCAAAAGCTGGAAAAGATGCTGATCGAGAAGCTCAACTACCTCGTGTTT
AAGGATAACGAGTTCGACAAGACTGGCGGCGTGCTGCGAGCCTACCAGC
TGACCGCTCCTTTCGAGACTTTCAAGAAGATGGGCAAGCAGACCGGCATC
ATCTACTACGTGCCCGCTGGCTTTACCTCTAAGATCTGCCCCGTGACCGG
CTTCGTCAACCAGCTGTACCCTAAGTACGAATCTGTGTCTAAGTCGCAAG
AGTTCTTTTCGAAGTTTGACAAGATCTGCTACAACCTCGACAAGGGATAC
TTCGAGTTCTCGTTCGACTATAAGAACTTCGGCGACAAGGCCGCCAAGGG

	CAAGTGGACCATTGCTTCTTTCGGCTCCCGACTGATCAACTTCCGAAACTCCGACAAGAACCACAACTGGGACACCCGAGAGGTTTACCCCACTAAGGAACTTGAGAAGCTGCTGAAGGACTACTCCATCGAGTACGGCCACGGCGAGTGCATCAAGGCTGCCATCTGCGGCGAGTCTGACAAGAAGTTCTTCGCCAAGCTGACCTCTGTGCTGAACACCATCCTGCAGATGCGAAACTCTAAGACCGGCACCGAGCTGGACTACCTGATCTCTCCCGTGGCCGACGTGAACGGCAACTTCTTCGACTCTCGACAGGCTCCCAAGAACATGCCCCAGGACGCCGACGCCAACGGCGCCTACCACATCGGCCTGAAGGGCCTGATGCTGCTGGGCCGAATCAAGAACAACCAGGAAGGCAAGAAGCTGAACCTCGTCATCAAGAACGAAGAGTACTTTGAGTTCGTGCAGAACAGAAACAAC*tctcgagccgac(linker)*cccaagaagaagcgaaaggtg(SV40 NLS) *ggatctggtggaggctcctctaagctgggcggctctggcggctctatggagcgagtgcgaatgatcaacgtgcagcgactgctggaggccgccgagttcctggagcgacgagagcgagagtgcgagcacggctacgcctcttctttcccctctatgccctctccccgaggctag (Mix1)*

Table S7 The establishment of RNAi.

Items	Coding sequences
Ri1	AtgctcaaccttagaaccgcccttcgagctgtgcgacccgtcactctggtgagtatctcggagcccgggacggctaccaacacacaagcaagatgcaacagaaaccggactttttaaatgcggattgcggaaaatttgcatggcggcaacgactcggagaaggagcgggaACGTGAGGGTGGAGGGCTCCTTTCCCGTGACCATGCTTCCGGGAGACGGTGTGGGGCCTGAGCTGATG(linker)tcccgctccttctccgagtcgttgccgccatgcaaattttccgcaatccgcatttaaaaagtccggtttctgttgcatcttgcttgtgtgttggtagccgtcccgggctccgagatactcaccagagtgacgggtcgcacagctcgaagggcggttctaaggttgagcat
Ri2	atgctcaaccttagaaccgcccttcgagctgtgcgacccgtcactctggtgagtatctcggagcccgggacggctaccaacacacaagcaagatgcaacagaaaccggactttttaaatgcggattgcggaaaatttgcatggcggcaacgactcggagaaggagcgggacaattgcaatggcaggatgccattgacgaactgagggtgatgagagaccgggcctccgatgacgtggtggtgacgacagcccggctggtgttgccgggactgtctctgaaaagcaatttctctatctccggtctcaacagactccccttctctagctcaattggcattgtcttcagaaggtgtcttagtggtatccccattgttatcttcttttccccaatgtcaatgtcaatgtcaatggctccgacctctttcacattaacacggcgcaaacacagataccacggaaccgactcaaacaaatccaaagagacgcagcggaataattggACGTGAGGGTGGAGGGCTCCTTTCCCGTGACCATGCTTCCGGGAGACGGTGTGGGGCCTGAGCTGATG(linker)ccaattattccgctgcgtctctttggatttgtttgagtcggttccgtggtatctgtgtttgcgccgtgttaatgtgaaagaggtcggagccattgacattgacattgacattggggaaaagaagataacaatggggataccactaagacaccttctgaagacaatgccaattgagctagagaaggggagtctgttgagaccggagatagagaaattgcttttcagagacagtcccggcaacaccagccgggctgtcgtcaccaccacgtcatcggaggcccggtctctcatcaccctcagttcgtcaatggcatcctgccattgcaattgtcccgctccttctccgagtcgttgccgccatgcaaattttccgcaatccgcatttaaaaagtccggtttctgttgcatcttgcttgtgtgttggtagccgtcccgggctccgagatactcaccagagtgacgggtcgcacagctcgaagggcggttctaaggttgagcat
Ri3	atgctcaaccttagaaccgcccttcgagctgtgcgacccgtcactctggtgagtatctcggagcccgggacggctaccaacacacaagcaagatgcaacagaaaccggactttttaaatgcggattgcggaaaatttgcatggcggcaacgactcggagaaggagcgggacaattgcaatggcaggatgccattgacgaactgagggtgatgagagaccgggcctccgatgacgtggtggtgacgacagcccggctggtgttgccgggactgtctctgaaaagcaatttctctatctccggtctcaacagactccccttctctagctcaattggcattgtcttcagaaggtgtcttagtggtatccccattgttatcttcttttccccaatgtcaatgtcaatgtcaatggctccgacctctttcacattaacacggcgcaaacacagataccacggaaccgactcaaacaaatccaaagagacgcagcggaataattggcatcaacgaacgatttgggatactctggcgagaatgccgaaatatttcgcttgtcttgttgtttctcttgagtgagttgtttgtgaagtcgtttggaagaaggttcccaatgtcacaaaccataccaactcgttacagccagcttgtaatcccccacctcttcaatacatactaacgcagacccgatcctacgccacttccgtggcctctttcaccggccagaagaactccaacggcaagtacactgtgtctctgattgagggagacggtatcggaaccgagatctccaaggctgtcaaggacatctaccatgccgccaaggtccccatcgactgggaggttgtcgacgtcacccccactctggtcaacggcaagaccaccatccccgacagcgccattgagtccatcaaccgaaacaaggttgccctcaagggtcccctcgccacccccatcggtaagggccacgtttccatgaacctgactctgcgacgaaccttcaaACGTGAGGGTGGAGGGCTCCTTTCCCGTGACCATGCTTCCGGGAGACGGTGTGGGGCCTGAGCTGATG(linker)ttgaaggttcgtcgcagagtcaggttcatggaaacgtggcccttaccgatgggggtggcgaggggacccttgagggcaaccttgtttcggttgatggactcaatggcgctgtcggggatggtggtcttgccgttgaccagagtgggggtgacgtcgacaacctcccagtcgatggggaccttggcggcatggtagatgtccttgacagccttggagatctcggttccgataccgtctccctcaatcagagacacagtgtacttgccgttggagttcttctggccggtgaaagaggccacggaagtggcgtaggatcgggtctgcgttagtatgtattgaagaggtgggggattacaagctggctgtaacgagttggtatggtttgtgacattgggaaccttcttccaaacgacttcacaaacaactcactcaagagaaacaacaagacaagcgaaatatttcggcattctcgccagagtatcccaaatcgttcgttgatgccaattattccgctgcgtctctttggatttgtttgagtcggttccgtggtatctgtgtttgcgccgtgttaatgtgaaagaggtcggagccattgacattgacattgacattggggaaaagaagataacaatggggataccactaagacaccttctgaagacaatgccaattgagctagagaaggggagtctgttgagaccggagatagagaaattgcttttcagagacagtcccggcaacaccagccgggctgtcgtcaccaccacgtcatcggaggcccggtctctcatcaccctcagttcgtcaatggcatcctgccattgcaattgtcccgctccttctccgagtcgttgccgccatgcaaattttccgcaatccgcatttaaaaagtccggtttctgttgcatcttgcttgtgtgttggtagccgtcccgggctccgagatactcaccagagtgacgggtcgcacagctcgaagggcggttctaaggttgagcat
Ri4	atgctcaaccttagaaccgcccttcgagctgtgcgacccgtcactctggtgagtatctcggagcccgggacggctaccaacacacaagcaagatgcaacagaaaccggactttttaaatgcggattgcggaaaatttgcatggcggcaacgactcggagaaggagcgggacaattgcaatggcaggatgccattgacgaactgagggtgatgagagaccgggcctccgatgacgtggtggtgacgacagcccggctggtgttgccgggactgtctctgaaaagcaatttctctatctccggtctcaacagactccccttctctagctcaattggcattgtcttcagaaggtgtcttagtggtatccccattgttatcttcttttccccaatgtcaatgtcaatgtcaatggctccgacctctttcacattaacacggcgcaaacacagataccacggaaccgactcaaacaaatccaaagagacgcagcggaataattggcatcaacgaacgatttgggatactctggcgagaatgccgaaatatttcgcttgtcttgttgtttctcttgagtgagttgtttgtgaagtcgtttggaagaa

	ggttcccaatgtcacaaaccataccaactcgttacagccagcttgtaatcccccacctcttcaatacatactaacgcagacccgatc<br>ctacgccacttccgtggcctctttcaccggccagaagaactccaacggcaagtacactgtgtctctgattgagggagacggtatc<br>ggaaccgagatctccaaggctgtcaaggacatctaccatgccgccaaggtccccatcgactgggaggttgtcgacgtcacccc<br>cactctggtcaacggcaagaccaccatccccgacagcgccattgagtccatcaaccgaaacaaggttgccctcaagggtcccc<br>tcgccacccccatcggtaagggccacgtttccatgaacctgactctgcgacgaaccttcaacctgttcgccaacgtccgaccttg<br>caagtccgtcgtgggctacaagaccccttacgagaacgtcgacaccctgctcatccgagagaacactgagggtgagtactccg<br>gtatcgagcacaccgtcgtccccggtgtcgttcagtccatcaagctgatcacccgagaggcttccgagcgagtcatccggtacg<br>cttacgagtacgccctgtcccgaggcatgaagaaggtccttgttgtccacaaggcctctattatgaaggtctccgatggtcttttcct<br>tgaggttgctcgagagctcgccaaggagtacccctccattgacctttccgtcgagctgatcgacaacacctgtctgcgaatggtc<br>caggaccccgctctctaccgagatgtcgtcatggtcatgcccaacctttacggtgacattctgtccgatcttgcctccggtcttatcg<br>gtggtcttggtctgacccccctccggtaacatgggtgacgaggtctccatcttcgaggccgtccacggatccgctcccgacattgct<br>ggcaagggtcttgctaaccccactgctctgctgctctcctccgtgatgatgctgcgacacatgggtctcaacgacaacgccacca<br>acatcgagcaggccgtctttggcaccattgcttccggccccgagaaccgaaccaaggatcttaagggtaccgccaccacttctc<br>actttgctgagcagattatcaagcgactcaagtagACGTGAGGGTGGAGGGCTCCTTTCCCGTGAC<br>CATGCTTCCGGGAGACGGTGTGGGGCCTGAGCTGATG(linker)ctacttgagtcgcttgata<br>atctgctcagcaaagtgagaagtggtggcggtacccttaagatccttggttcggttctcggggccggaagcaatggtgccaaag<br>acggcctgctcgatgttggtggcgttgtcgttgagacccatgtgtcgcagcatcatcacggaggagagcagcagagcagtggg<br>gttagcaagacccttgccagcaatgtcgggagcggatccgtggacggcctcgaagatggagacctcgtcacccatgttaccgg<br>agggggtcagaccaagaccaccgataagaccggaggcaagatcggacagaatgtcaccgtaaaggttgggcatgaccatga<br>cgacatctcggtagagagcggggtcctggaccattcgcagacaggtgttgtcgatcagctcgacggaaaggtcaatggaggg<br>gtactccttggcgagctctcgagcaacctcaaggaaaagaccatcggagaccttcataatagaggccttgtggacaacaaggac<br>cttcttcatgcctcgggacagggcgtactcgtaagcgtaccggatgactcgctcggaagcctctcgggtgatcagcttgatggac<br>tgaacgacaccggggacgacggtgtgctcgataccggagtactcaccctcagtgttctctcggatgagcagggtgtcgacgttc<br>tcgtaaggggtcttgtagcccacgacggacttgcaaggtcggacgttggcgaacaggttgaaggttcgtcgcagagtcaggttc<br>atggaaacgtggcccttaccgatgggggtggcgaggggacccttgagggcaaccttgtttcggttgatggactcaatggcgctg<br>tcggggatggtggtcttgccgttgaccagagtgggggtgacgtcgacaacctcccagtcgatggggaccttggcggcatggta<br>gatgtccttgacagccttggagatctcggttccgataccgtctccctcaatcagagacacagtgtacttgccgttggagttcttctgg<br>ccggtgaaagaggccacggaagtggcgtaggatcgggtctgcgttagtatgtattgaagaggtgggggattacaagctggctgt<br>aacgagttggtatggtttgtgacattgggaaccttcttccaaacgacttcacaaacaactcactcaagagaaacaacaagacaag<br>cgaaatatttcggcattctcgccagagtatcccaaatcgttcgttgatgccaattattccgctgcgtctctttggatttgtttgagtcggt<br>tccgtggtatctgtgtttgcgccgtgttaatgtgaaagaggtcggagccattgacattgacattgacattggggaaaagaagataa<br>caatggggataccactaagacaccttctgaagacaatgccaattgagctagagaaggggagtctgttgagaccggagatagag<br>aaattgcttttcagagacagtcccggcaacaccagccgggctgtcgtcaccaccacgtcatcggaggcccggtctctcatcacc<br>ctcagttcgtcaatggcatcctgccattgcaattgtcccgctccttctccgagtcgttgccgccatgcaaattttccgcaatccgcatt<br>taaaaagtccggtttctgttgcatcttgcttgtgtgttggtagccgtcccgggctccgagatactcaccagagtgacgggtcgcaca<br>gctcgaagggcggttctaaggttgagcat
Ri5	tttcttgtcgccaacttcgtggaacccccaaagaaatcacaatgctcaaccttagaaccgcccttcgagctgtgcgacccgtcact<br>ctggtgagtatctcggagcccgggacggctaccaacacacaagcaagatgcaacagaaaccggactttttaaatgcggattgc<br>gACGTGAGGGTGGAGGGCTCCTTTCCCGTGACCATGCTTCCGGGAGACGGTGT<br>GGGGCCTGAGCTGATG(linker)cgcaatccgcatttaaaaagtccggtttctgttgcatcttgcttgtgtgttggta<br>gccgtcccgggctccgagatactcaccagagtgacgggtcgcacagctcgaagggcggttctaaggttgagcattgtgatttctt<br>tgggggttccacgaagttggcgacaagaaa
Ri6	tcaaccttagaaccgcccttcgagctgtgcgacccgtcactctggtgagtatctcggagcccgggacggctaccaacacacaag<br>caagatgcaacagaaaccggactttttaaatgcggattgcggaaaatttgcatggcggcaacgactcggagaaggagcggga<br>ACGTGAGGGTGGAGGGCTCCTTTCCCGTGACCATGCTTCCGGGAGACGGTGT<br>GGGGCCTGAGCTGATG(linker)tcccgctccttctccgagtcgttgccgccatgcaaattttccgcaatccgcatt<br>taaaaagtccggtttctgttgcatcttgcttgtgtgttggtagccgtcccgggctccgagatactcaccagagtgacgggtcgcaca<br>gctcgaagggcggttctaaggttga
Ri7	tgagtatctcggagcccgggacggctaccaacacacaagcaagatgcaacagaaaccggactttttaaatgcggattgcggaa<br>aatttgcatggcggcaacgactcggagaaggagcgggaACGTGAGGGTGGAGGGCTCCTTTCCCGT<br>GACCATGCTTCCGGGAGACGGTGTGGGGCCTGAGCTGATG(linker)tcccgctccttctc<br>cgagtcgttgccgccatgcaaattttccgcaatccgcatttaaaaagtccggtttctgttgcatcttgcttgtgtgttggtagccgtcc<br>cgggctccgagatactca
Ri8	cgcaatccgcatttaaaaagtccggtttctgttgcatcttgcttgtgtgttggtagccgtcccgggctccgagatactcaccagagtg<br>acgggtcgcacagctcgaagggcggttctaaggttgagcattgtgatttctttgggggttccacgaagttggcgacaagaaa

Ri9	ctacttgagtcgcttgataatctgctcagcaaagtgagaagtggtggcggtacccttaagatccttggttcggttctcggggccggaagcaatggtgccaaagacggcctgctcgatgttggtggcgttgtcgttgagacccatgtgtcgcagcatcatcacggaggagagcagcagagcagtggggttagcaagacccttgccagcaatgtcgggagcggatccgtggacggcctcgaagatggagacctcgtcacccatgttaccggagggggtcagaccaagaccaccgataagaccggaggcaagatcggacagaatgtcaccgtaaaggttgggcatgaccatgacgacatctcggtagagagcggggtcctggaccattcgcagacaggtgttgtcgatcagctcgacggaaaggtcaatggaggggtactccttggcgagctctcgagcaacctcaaggaaaagaccatcggagaccttcataatagaggccttgtggacaacaaggaccttcttcatgcctcgggacagggcgtactcgtaagcgtaccggatgactcgctcggaagcctctcgggtgatcagcttgatggactgaacgacaccggggacgacggtgtgctcgataccggagtactcaccctcagtgttctctcggatgagcagggtgtcgacgttctcgtaaggggtcttgtagcccacgacggacttgcaaggtcggacgttggcgaacaggttgaaggttcgtcgcagagtcaggttcatggaaacgtggcccttaccgatgggggtggcgaggggacccttgagggcaaccttgtttcggttgatggactcaatggcgctgtcggggatggtggtcttgccgttgaccagagtgggggtgacgtcgacaacctcccagtcgatggggaccttggcggcatggtagatgtccttgacagccttggagatctcggttccgataccgtctccctcaatcagagacacagtgtacttgccgttggagttcttctggccggtgaaagaggccacggaagtggcgtaggatcgggtctgcgttagtatgtattgaagaggtgggggattacaagctggctgtaacgagttggtatggtttgtgacattgggaaccttcttccaaacgacttcacaaacaactcactcaagagaaacaacaagacaagcgaaatatttcggcattctcgccagagtatcccaaatcgttcgttgatgccaattattccgctgcgtctctttggatttgtttgagtcggttccgtggtatctgtgtttgcgccgtgttaatgtgaaagaggtcggagccattgacattgacattgacattggggaaaagaagataacaatggggataccactaagacaccttctgaagacaatgccaattgagctagagaaggggagtctgttgagaccggagatagagaaattgcttttcagagacagtcccggcaacaccagccgggctgtcgtcaccaccacgtcatcggaggcccggtctctcatcaccctcagttcgtcaatggcatcctgccattgcaattgtcccgctccttctccgagtcgttgccgccatgcaaattttccgcaatccgcatttaaaaagtccggtttctgttgcatcttgcttgtgtgttggtagccgtcccgggctccgagatactcaccagagtgacgggtcgcacagctcgaagggcggttctaaggttgagcat
Ri10	gaaggttcgtcgcagagtcaggttcatggaaacgtggcccttaccgatgggggtggcgaggggacccttgagggcaaccttgtttcggttgatggactcaatggcgctgtcggggatggtggtcttgccgttgaccagagtgggggtgacgtcgacaacctcccagtcga

Table S8 Media with varying Carbon / Nitrogen (C/N) ratios, PL, and SL conditions

Media	Comments	Note
10 g/L $(NH_4)_2SO_4$, 3 g/L KH_2PO_4, 0.5 g/L $MgSO_4$•$7H_2O$, 2 mL trace metals solution stock, and 1 mL of vitamin solution stock, 100 g/L initial glucose. 650 g/L glucose was fed when residual glucose is below 20 g/L.	C/N=22	Fig. 6a
10 g/L $(NH_4)_2SO_4$, 19.1 g/L KH_2PO_4, 10.4 g/L K_2HPO_4, 0.5 g/L $MgSO_4$•$7H_2O$, 39 g/L MES (0.2M), 2 mL trace metals solution stock, and 1 mL of vitamin solution stock, 100g/L glucose.	NR	Fig. 6b
2.5 g/L $(NH_4)_2SO_4$, 19.1 g/L KH_2PO_4, 10.4 g/L K_2HPO_4, 0.5 g/L $MgSO_4$•$7H_2O$, 39 g/L MES (0.2M), 2 mL trace metals solution stock, and 1 mL of vitamin solution stock, 100g/L glucose.	NL	Fig. 6b
10 g/L $(NH_4)_2SO_4$, 0.15 g/L KH_2PO_4, 0.5 g/L $MgSO_4$•$7H_2O$, 39 g/L MES (0.2M), 2 mL trace metals solution stock, and 1 mL of vitamin solution stock, 100g/L glucose.	PL	Fig. 6b
8 g/L $(NH_4)_2Cl$, 19.1 g/L KH_2PO_4, 10.4 g/L K_2HPO_4, 0.005 g/L $MgSO_4$•$7H_2O$, 0.17 g/L $MgCl_2$, 39 g/L MES (0.2M), 2 mL trace metals solution stock, and 1 mL of vitamin solution stock, 100g/L glucose.	SL	Fig. 6b
10 g/L $(NH_4)_2SO_4$, 3 g/L KH_2PO_4, 0.5 g/L $MgSO_4$•$7H_2O$, 2 mL trace metals solution stock, and 1 mL of vitamin solution stock, 100 g/L initial glucose. 650 g/L glucose was fed when residual glucose is below 20 g/L.	NR(C/N=22) ->NL	Fig. 6c
2.5 g/L $(NH_4)_2SO_4$, 3 g/L KH_2PO_4, 0.5 g/L $MgSO_4$•$7H_2O$, 2 mL trace metals solution stock, and 1 mL of vitamin solution stock, 100 g/L initial glucose. 650 g/L glucose was fed when residual glucose is below 20 g/L.	NL	Fig. 6c
10 g/L $(NH_4)_2SO_4$, 0.2 g/L KH_2PO_4, 0.5 g/L $MgSO_4$•$7H_2O$, 2 mL trace metals solution stock, and 1 mL of vitamin solution stock, 100 g/L initial glucose.	PL	Fig. 6c
10 g/L $(NH_4)_2SO_4$, 3 g/L KH_2PO_4, 0.1 g/L $MgSO_4$•$7H_2O$, 0.17 g/L $MgCl_2$, 2 mL trace metals solution stock, and 1 mL of vitamin solution stock, 100 g/L initial glucose.	SL	Fig. 6c
10 g/L $(NH_4)_2SO_4$, 3 g/L KH_2PO_4, 0.5 g/L $MgSO_4$•$7H_2O$, 2 mL trace metals solution stock, and 1 mL of vitamin solution stock, 100 g/L initial glucose. 650 g/L glucose was fed when residual glucose is below 20 g/L. 2.5, 1, 0.1, and 0 g/L yeast extract was added for different conditions.	Test the effect of yeast extract	Fig. 6d
10 g/L $(NH_4)_2SO_4$, 3 g/L KH_2PO_4, 0.5 g/L $MgSO_4$•$7H_2O$, 2 mL trace metals solution stock, and 1 mL of vitamin solution stock, 100 g/L initial glucose. 650 g/L glucose with 20 g/L yeast extract was fed continuously.	pH effect test	Fig. 6e
7.5 g/L $(NH_4)_2SO_4$, 19.1 g/L KH_2PO_4, 10.4 g/L K_2HPO_4, 0.5 g/L $MgSO_4$•$7H_2O$, 39 g/L MES (0.2M), 2 mL trace metals solution stock, and 1 mL of vitamin solution stock with various concentrations of IA (0, 5, 10, 20, 40 g/L) or 20 g/L NaCl as a negative control to test the IA tolerance. The initial pH was 6.5.	IA tolerance test	Fig. 6f
10 g/L $(NH_4)_2SO_4$, 3 g/L KH_2PO_4, 0.5 g/L $MgSO_4$•$7H_2O$, 2 mL trace metals solution stock, and 1 mL of vitamin solution stock, 100 g/L initial glucose. 650 g/L glucose with 20 g/L yeast extract was fed when residual glucose is below 20 g/L.	NR->NL	Fig. 6g
2.5 g/L $(NH_4)_2SO_4$, 3 g/L KH_2PO_4, 0.5 g/L $MgSO_4$•$7H_2O$, 2 mL trace metals solution stock, and 1 mL of vitamin solution stock, 100 g/L initial glucose. 650 g/L glucose with 20 g/L yeast extract was fed when residual glucose is below 20 g/L.	NL	Fig. 6g
10 g/L $(NH_4)_2SO_4$, 0.2 g/L KH_2PO_4, 0.5 g/L $MgSO_4$•$7H_2O$, 2 mL trace metals solution stock, and 1 mL of vitamin solution stock, 100 g/L initial glucose. 650 g/L glucose with 20 g/L yeast extract was fed when residual glucose is below 20 g/L.	PL	Fig. 6g
10 g/L $(NH_4)_2SO_4$, 3 g/L KH_2PO_4, 0.1 g/L $MgSO_4$•$7H_2O$, 0.17 g/L $MgCl_2$, 2 mL trace metals solution stock, and 1 mL of vitamin solution stock, 100 g/L initial glucose. 650 g/L glucose with 20 g/L yeast extract was fed when residual glucose is below 20 g/L.	SL	Fig. 6g
10 g/L $(NH_4)_2SO_4$, or 4.5 g/L urea, 3 g/L KH_2PO_4, 0.5 g/L $MgSO_4$•$7H_2O$, 2 mL trace metals solution stock, and 1 mL of vitamin solution stock, 100 g/L initial glucose. 650 g/L glucose with 20 g/L yeast extract was fed continuously. Nitrogen source was fed during fermentation.	1L fed-batch	Fig. 6h
9 g/L urea, 6 g/L KH_2PO_4, 1 g/L $MgSO_4$•$7H_2O$, 4 mL trace metals solution stock, and 2 mL of vitamin solution stock, 100 g/L initial glucose. 650 g/L glucose with 20 g/L yeast extract was fed continuously. 400 g/L urea was fed during fermentation.	50L fed-batch	Fig. 6i
